# Personalized neoantigen vaccine and pembrolizumab in advanced hepatocellular carcinoma: a phase 1/2 trial

**DOI:** 10.1038/s41591-024-02894-y

**Published:** 2024-04-07

**Authors:** Mark Yarchoan, Edward J. Gane, Thomas U. Marron, Renzo Perales-Linares, Jian Yan, Neil Cooch, Daniel H. Shu, Elana J. Fertig, Luciane T. Kagohara, Gabor Bartha, Josette Northcott, John Lyle, Sarah Rochestie, Joann Peters, Jason T. Connor, Elizabeth M. Jaffee, Ildiko Csiki, David B. Weiner, Alfredo Perales-Puchalt, Niranjan Y. Sardesai

**Affiliations:** 1 Department of Oncology, Sidney Kimmel Comprehensive Cancer Center, Johns Hopkins University School of Medicine, Baltimore, MD USA; 2 New Zealand Liver Transplant Unit, University of Auckland, Auckland, New Zealand; 3 Early Phase Trials Unit, Tisch Cancer Institute, Icahn School of Medicine at Mount Sinai, New York, NY USA; 4 Geneos Therapeutics, Philadelphia, PA USA; 5 Department of Biomedical Engineering, Johns Hopkins University School of Medicine, Baltimore, MD USA; 6 Department of Applied Mathematics and Statistics, Johns Hopkins University Whiting School of Engineering, Baltimore, MD USA; 7 Personalis, Inc., Fremont, CA USA; 8 ConfluenceStat, Cooper City, FL USA; 9 University of Central Florida College of Medicine, Orlando, FL USA; 10 Vaccine and Immunotherapy Center, The Wistar Institute, Philadelphia, PA USA

**Keywords:** Translational research, Liver cancer, Immunization, DNA vaccines, Antigen presentation

## Abstract

Programmed cell death protein 1 (PD-1) inhibitors have modest efficacy as a monotherapy in hepatocellular carcinoma (HCC). A personalized therapeutic cancer vaccine (PTCV) may enhance responses to PD-1 inhibitors through the induction of tumor-specific immunity. We present results from a single-arm, open-label, phase 1/2 study of a DNA plasmid PTCV (GNOS-PV02) encoding up to 40 neoantigens coadministered with plasmid-encoded interleukin-12 plus pembrolizumab in patients with advanced HCC previously treated with a multityrosine kinase inhibitor. Safety and immunogenicity were assessed as primary endpoints, and treatment efficacy and feasibility were evaluated as secondary endpoints. The most common treatment-related adverse events were injection-site reactions, observed in 15 of 36 (41.6%) patients. No dose-limiting toxicities or treatment-related grade ≥3 events were observed. The objective response rate (modified intention-to-treat) per Response Evaluation Criteria in Solid Tumors 1.1 was 30.6% (11 of 36 patients), with 8.3% (3 of 36) of patients achieving a complete response. Clinical responses were associated with the number of neoantigens encoded in the vaccine. Neoantigen-specific T cell responses were confirmed in 19 of 22 (86.4%) evaluable patients by enzyme-linked immunosorbent spot assays. Multiparametric cellular profiling revealed active, proliferative and cytolytic vaccine-specific CD4^+^ and CD8^+^ effector T cells. T cell receptor β-chain (TCRβ) bulk sequencing results demonstrated vaccination-enriched T cell clone expansion and tumor infiltration. Single-cell analysis revealed posttreatment T cell clonal expansion of cytotoxic T cell phenotypes. TCR complementarity-determining region cloning of expanded T cell clones in the tumors following vaccination confirmed reactivity against vaccine-encoded neoantigens. Our results support the PTCV’s mechanism of action based on the induction of antitumor T cells and show that a PTCV plus pembrolizumab has clinical activity in advanced HCC. ClinicalTrials.gov identifier: NCT04251117.

## Abstract (web summary)

Treatment of patients with advanced hepatocellular carcinoma with a personalized DNA vaccine in combination with anti-PD-1 therapy was safe and led to encouraging clinical efficacy, with immunological analyses confirming the induction of tumor antigen-specific T cell responses.

## Main

Hepatocellular carcinoma (HCC) is the most common form of primary liver cancer and is a leading cause of cancer-related death worldwide^1^. Despite recent advancements in systemic therapy for advanced HCC, the 5-year survival rate remains <10% (ref. ^2^). Advanced HCC is a relatively immune-resistant tumor type generally characterized by low T cell infiltration and a modest tumor mutational burden (TMB)^3^. Immune checkpoint inhibitors (ICIs) targeting programmed cell death protein 1 (PD-1) have response rates of approximately 12–18% as a monotherapy^4–10^.

Mutations within the tumor genome cause tumors to express abnormal proteins that are not found in any normal host cell, called mutation-associated neoantigens (MANAs)^11,12^. Advancements in next-generation sequencing facilitate the development of personalized immunotherapies targeting MANAs for an individual cancer patient^13^. Patients with preexisting immunity to tumor neoantigens often have robust responses to ICIs^14^, providing an initial rationale for combining ICIs with therapies that induce neoantigen-specific immunity. In preclinical studies, therapeutic cancer vaccines targeting MANAs induced tumor-specific T cell responses and impeded tumor growth^15–18^. Initial clinical trials of personalized therapeutic cancer vaccines (PTCVs) have demonstrated the induction of neoantigen-specific immune responses in patients^19–22^. Recently, data from the phase 2b randomized KEYNOTE-942 study provided initial evidence of clinical efficacy in a highly immune-sensitive tumor type in the absence of measurable disease^23^. However, whether vaccine-induced T cells traffic into established tumors and can induce tumor clearance in combination with anti-PD-1 therapy in less immunotherapy-responsive tumor types, such as HCC, has not been established.

We conducted a 36-patient, single-arm, open-label, multicenter phase 1/2 study of a PTCV in combination with pembrolizumab (a PD-1 inhibitor) in patients with advanced HCC previously treated with a multityrosine kinase inhibitor (mTKI). The PTCV consisted of a DNA plasmid encoding up to 40 neoantigens (GNOS-PV02) identified through sequencing of each patient’s tumor DNA and RNA, as well as their germline DNA, as described previously^24^. GNOS-PV02 is coformulated with a second DNA plasmid encoding the cytokine interleukin-12 (IL-12) as a vaccine adjuvant (pIL12) and administered by intradermal injection followed by in vivo electroporation. Intradermal injection of pIL12 results in only local and transient production of IL-12 at the injection site and facilitates the localized induction of cellular responses to the expressed antigens^25,26^. The primary study endpoints were safety and immunogenicity.

## Results

### Safety, feasibility and clinical responses

**Fig. 1:**
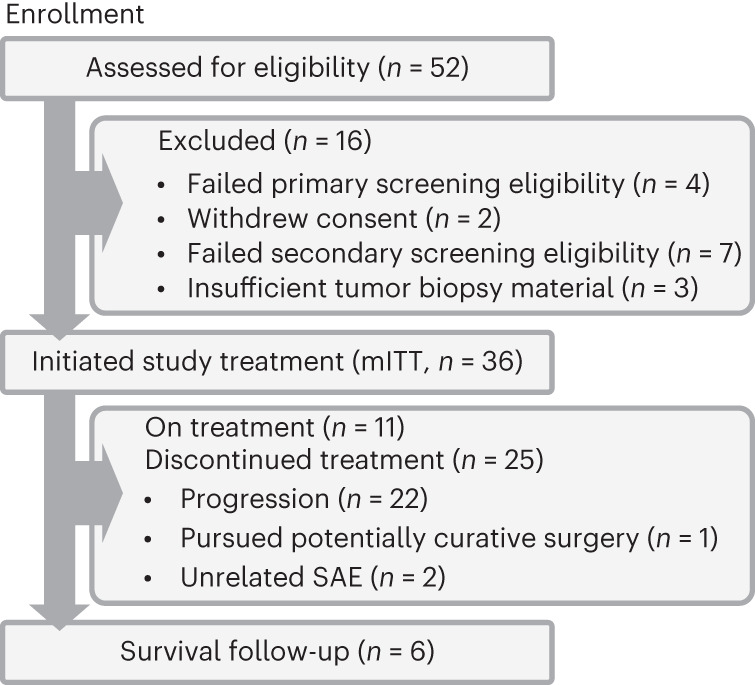
Patient flowchart. The CONSORT (Consolidated Standards of Reporting Trials) diagram shows the flow of patients as of August 18, 2023. SAE, severe adverse event.

**Fig. 2:**
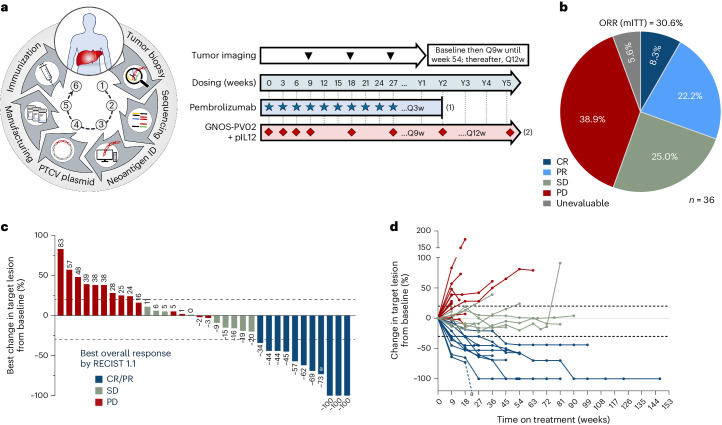
Clinical response. **a**, Manufacturing process for GNOS-PV02 and clinical trial design. In patients without disease progression, (1) treatment with pembrolizumab may continue every 3 weeks (Q3w) for 2 years per label recommendation; (2) treatment with GNOS-PV02 + pIL12 may continue Q3w for four doses, followed by Q9w until year 2 (Y2) and Q12w beyond 2 years. **b**, Pie chart with the percentage ORR, CR, PR, SD and PD according to RECIST 1.1 (*n* = 36, mITT). **c**, Waterfall plot showing the best overall response achieved by the 34 evaluable patients of the GT-30 trial at the time of data cutoff (August 18, 2023). ^a^PR patient with a primary liver lesion and two lung metastases who achieved secondary resectability owing to tumor shrinkage and remained tumor-free for 18.2 months after the first treatment dose. **d**, Spider plot showing changes in the target lesion from baseline for the 34 evaluable patients of the GT-30 trial at the time of data cutoff (August 18, 2023). ^a^The same as in **c**.

The baseline demographic and clinical characteristics of the study population are shown in Table 1. The first and last patients were enrolled on June 16, 2020, and June 14, 2023, respectively. The trial is ongoing. The median number of vaccinations at the data cutoff date was 5 (range 1–18), and the median duration of treatment was 6.1 months. At the data cutoff date (August 18, 2023), 25 patients had discontinued the study therapy (Fig. 1). The most common reason for discontinuation was disease progression (*n* = 22). All 36 patients had their personalized vaccine product available for dosing at the time they were eligible to receive second-line therapy (Fig. 2a).

**Table 1:** GT-30 baseline patient demographic and clinical characteristics

Characteristics (*n* = 36)	*n* (%)
Median age, years (range)	66.5 (40–83)
Sex	
Female	11 (30.6%)
Male	25 (69.4%)
Race	
White	21 (58.4%)
Asian	8 (22.2%)
Other (Black and Pacific Islander)	7 (19.4%)
ECOG performance status	
0	25 (69.4%)
1	11 (30.6%)
Child–Pugh score A	36 (100%)
BCLC stage	
B	18 (50.0%)
C	18 (50.0%)
Etiology	
HBV	8 (22.2%)
HCV	12 (33.3%)
HBV + HCV	1 (2.8%)
Nonviral	15 (41.7%)
Prior treatment	
Sorafenib	2 (5.6%)
Sorafenib + lenvatinib	1 (2.8%)
Lenvatinib	33 (91.6%)
PVI	7 (19.4%)
Wnt/β-catenin mutation	10 (27.8%)
Baseline AFP, ng ml^−1^	
≥400	8 (22.2%)
<400	28 (77.8%)
Targetable neoantigens^a^	
≤20	10 (27.8%)
21–40	16 (44.4%)
41–67	10 (27.8%)

Data cutoff date: August 18, 2023. *n* = 36 patients.

BCLC, Barcelona Clinic Liver Cancer; ECOG, Eastern Cooperative Oncology Group; HBV, hepatitis B virus; HCV, hepatitis C virus; PVI, portal vein invasion.

^a^Each patient’s vaccine included up to 40 neoantigens.

Treatment-related adverse events (TRAEs) observed by the cutoff date are listed in Table 2. Overall, the treatment was safe and well tolerated, with an adverse event profile similar to that reported for pembrolizumab monotherapy in HCC, except for an increase in local vaccine injection-site reactions. Low-grade TRAEs were observed in 27 patients (75.0%), and there were no grade ≥3 TRAEs. Three patients (8.3%) experienced an immune-related adverse event (irAE) requiring systemic steroid treatment (grade 2 nephritis, grade 2 pneumonitis and grade 2 hepatitis). One patient (2.8%) discontinued pembrolizumab owing to an adverse event, but no patients discontinued PTCV therapy because of an adverse event.

**Table 2:** Overall summary of GT-30 TRAEs

TRAEs^a^ (*n* = 27/36)	Grade 1/2
Injection-site reactions	
Pain, erythema, pruritus or swelling	15 (41.6%)
Gastrointestinal	
Gastroesophageal reflux	2 (5.6%)
Diarrhea	1 (2.8%)
Abdominal pain (upper)	1 (2.8%)
Nausea	1 (2.8%)
Dry mouth	1 (2.8%)
Skin	
Rash	4 (11.1%)
Pruritus	2 (5.6%)
Dermatitis	1 (2.8%)
Alopecia	1 (2.8%)
Skin disorder	1 (2.8%)
Dry skin	1 (2.8%)
Endocrine	
Hypothyroidism	2 (5.6%)
Musculoskeletal	
Pain in extremities, musculoskeletal pain or stiffness	3 (8.3%)
Arthralgia	1 (2.8%)
Metabolism	
Decreased appetite	2 (5.6%)
Hypophosphatemia	1 (2.8%)
Polydipsia	1 (2.8%)
General	
Fatigue	4 (11.1%)
Pyrexia	1 (2.8%)
Chills	1 (2.8%)
Anemia	1 (2.8%)
Dysesthesia	1 (2.8%)
Lethargy	1 (2.8%)
Somnolence	1 (2.8%)
Infusion-related reaction	1 (2.8%)
Chest discomfort	1 (2.8%)
Hepatitis	1 (2.8%)
Elevated liver function test results	1 (2.8%)
Immune-related	
Immune-mediated nephritis	1 (2.8%)
Pneumonitis	1 (2.8%)
ICI hepatitis	1 (2.8%)
Use of systemic steroids	7 (19.4%)

^a^TRAEs were determined by the investigator for those events deemed as possibly, probably or definitely related to the PTCV, IL-12, electroporation and/or ICI.

At the time of data analysis, 34 of the 36 patients had undergone at least one on-treatment restaging scan and were evaluable for response according to Response Evaluation Criteria in Solid Tumors (RECIST) 1.1. Two patients discontinued therapy owing to unrelated severe adverse events (one after the first dose, one after the third dose of therapy) and were deemed unevaluable, but both patients were included in the modified intention-to-treat (mITT) analysis as nonresponders. By investigator review, the objective response rate (ORR) (confirmed + unconfirmed, mITT) per RECIST 1.1 was 30.6% (11 of 36 patients), with 8.3% (3 of 36) of patients achieving a complete response (CR) and 22.2% (8 of 36) of patients achieving a partial response (PR). The disease control rate was 55.6% (20 of 36 patients) (Fig. 2b–d). Of the 11 patients with an objective response, 9 patients (including the 3 patients with CR) had their response confirmed at the next regularly scheduled on-treatment imaging scan. Two patients with PR had their target lesions continue to show further tumor reduction over subsequent imaging time point(s), confirming a durable response (−44% and −59%), but were categorized as unconfirmed PR owing to the emergence of new nontarget lesions (one of these patients is described as a case study in ‘Vaccine-induced immune editing leads to tumor escape’ below).

**Extended Data Fig. 1:**
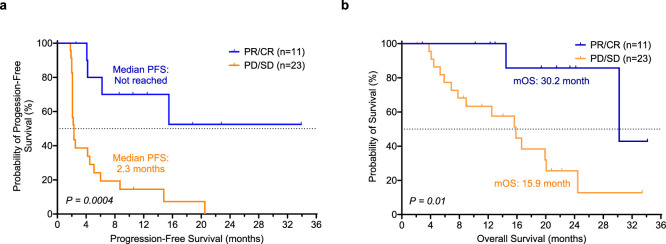
Association of clinical response with survival. **a**. Probability of progression-free survival in patients with partial and complete response (n = 11) and patients with stable disease and progression (n = 23). **b**. Probability of overall survival in patients with partial and complete response (n = 11) and patients with stable disease and progression (n = 23). Significance was evaluated by the Log-rank (Mantel-Cox) test.

**Extended Data Fig. 2:**
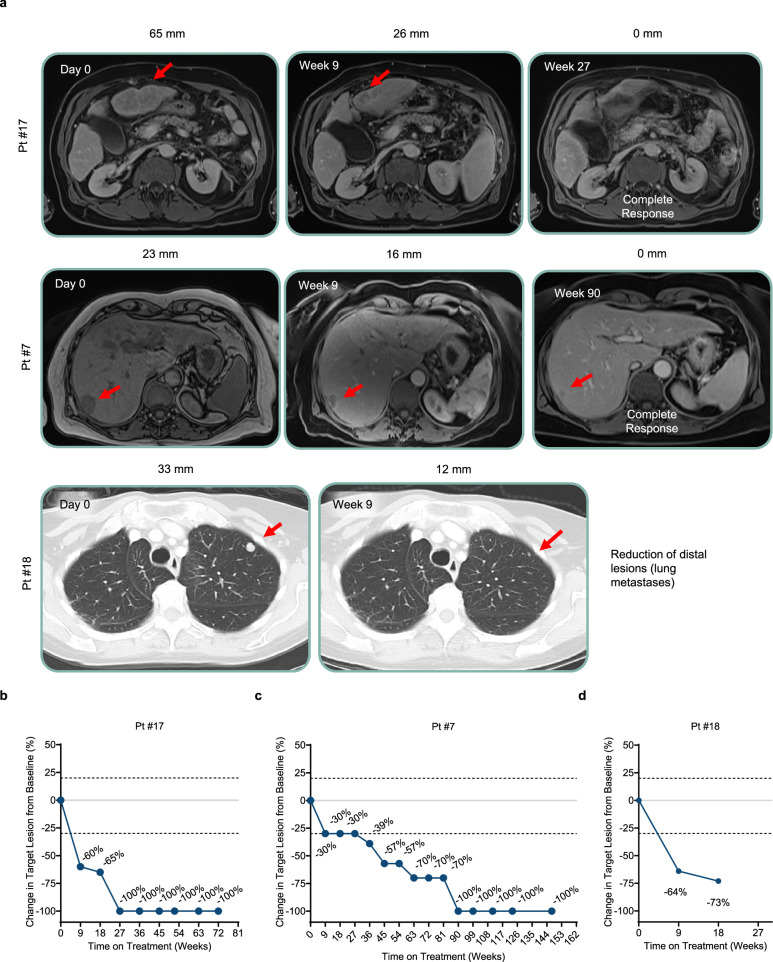
Representative clinical responses. **a**, Tumor imaging scans of GT-30 patients #17, #7, and #18 categorized as CR, CR and PR, respectively at various treatment time-points. Red arrows point at the tumor location. **b-d**, Change in target lesion from baseline (%) in three patients (Pt) #17 (CR), Pt #7 (CR), and Pt #18 (PR) evaluated by RECIST 1.1.

**Extended Data Fig. 3:**
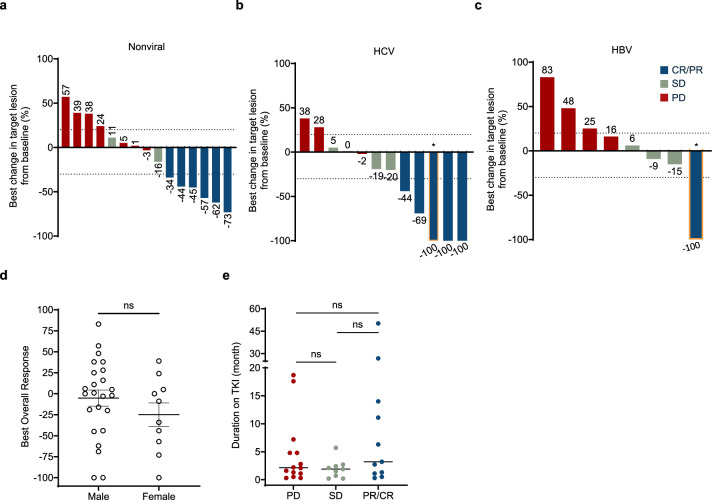
Clinical results across sex, etiologic disease subgroups, and TKI treatment. No apparent correlation of etiology (**a-c**), gender (male, n = 24; female, n = 10) (**d**), and time on TKI at baseline (PD, n = 14; SD, n = 9; PR/CR, n = 11) (**e**) with BOR. Note: 1 CR patient had a previous history of both HCV and HBV infection and is shown marked with an (*) in both (b) and (c). d, e, Data are presented as mean ± SEM. Significance was tested by a two-tailed, Mann-Whitney statistical analysis.

At the data cutoff, the median follow-up was 21.5 months. Initial response assessment was performed at 9 weeks; among patients who had a response, the median time to the response was 9.3 weeks (range 8–46 weeks). One patient with initially unresectable HCC achieved secondary resectability after five PTCV doses. The median progression-free survival (mPFS) was 4.2 months, and the median overall survival (mOS) was 19.9 months. The median duration of response was not reached. Clinical response (CR/PR versus stable disease (SD)/progressive disease (PD)) was significantly associated with survival (PFS and OS) (Extended Data Fig. 1). Example responses are shown in Extended Data Fig. 2a–d. Clinical results were generally consistent across sex, etiologic disease subgroups and time on first-line TKI treatment at baseline (Extended Data Fig. 3).

Anti-PD-1 agents have been studied extensively in both the first-line and second-line advanced HCC settings. Across registrational clinical trials of pembrolizumab, nivolumab, durvalumab and tislelizumab, the ORR ranged from 12% to 18% (refs. ^4–10,27^). For the protocol development, sample size estimation and prespecified statistical hypothesis testing, we used the comparator pembrolizumab ORR of 16.9% based on KN-240 (ref. ^28^), which was consistent with the 17.0% ORR for pembrolizumab monotherapy observed in the KN-224 phase 2 study^9^. The observed ORR of 30.6% (11 of 36 patients) achieved statistical significance with a one-sided *P* value of 0.031 (one-sided 90% confidence interval (CI), 20.4–100%) versus the prespecified historical control.

**Extended Data Fig. 4:**
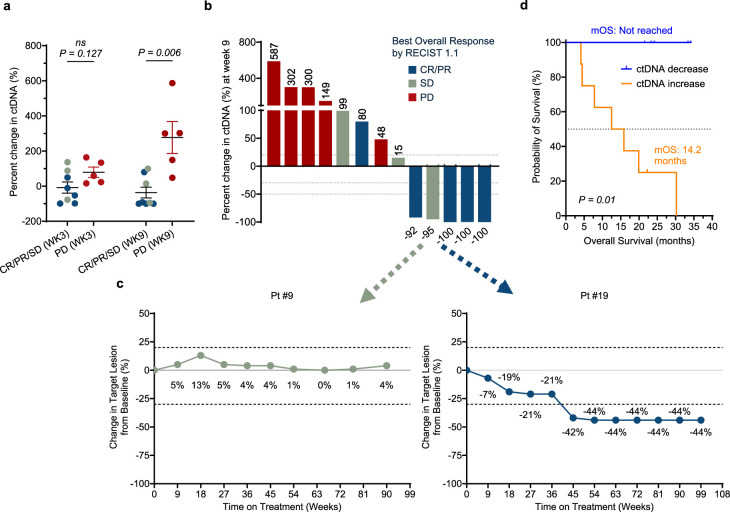
Change in ctDNA levels from baseline is predictive of clinical outcome to vaccine (n = 13). **a**, Percent change in ctDNA at weeks 3 and 9 relative to baseline levels in patients with disease control (CR/PR/SD, n = 8) vs patients with progressive disease (PD, n = 5). Each circle indicates an individual patient per group. Data are presented as mean ± SEM. All P-values were evaluated by a two-tailed, Mann-Whitney test, and P-values below 0.05 were considered statistically significant. **b**, Percent change in ctDNA (molecular response) at Week 9 relative to baseline levels shown as best overall response evaluated by RECIST 1.1. **c**, Change in target lesion from baseline (%) evaluated by RECIST 1.1 in two patients who had a deeper molecular response at 95% and 100% reduction in ctDNA relative to their objective response. **d**, Probability of survival in patients with (n = 5) or without (n = 8) reduction in ctDNA at week 9. Significance was evaluated by the Log-rank (Mantel-Cox) test.

We performed circulating tumor DNA (ctDNA) analyses using day 0 (baseline), week 3 and week 9 on-treatment samples from 13 patients. A molecular response was defined as a >50% reduction in the ctDNA level from baseline^22,29^. Changes in ctDNA levels broadly tracked with magnetic resonance imaging scans in monitoring objective responses (CR and PR). As shown in Extended Data Fig. 4a, the difference in the percentage change in ctDNA levels between patients with disease control (CR, PR or SD) and those with PD reached significance at week 9 (*P* = 0.006). The ctDNA analysis detected a stronger molecular response relative to imaging in two patients (Extended Data Fig. 4b,c). Patient 9 (determined to have SD by imaging) had a 95% reduction in ctDNA level, and patient 19 (determined to have PR by imaging) had a 100% reduction in ctDNA level. Both patients had durable responses lasting for more than 12 months. A ctDNA decrease at week 9 was significantly associated with longer survival (*P* = 0.01) (Extended Data Fig. 4d).

### Biomarker analysis of the observed clinical responses

**Extended Data Fig. 5:**
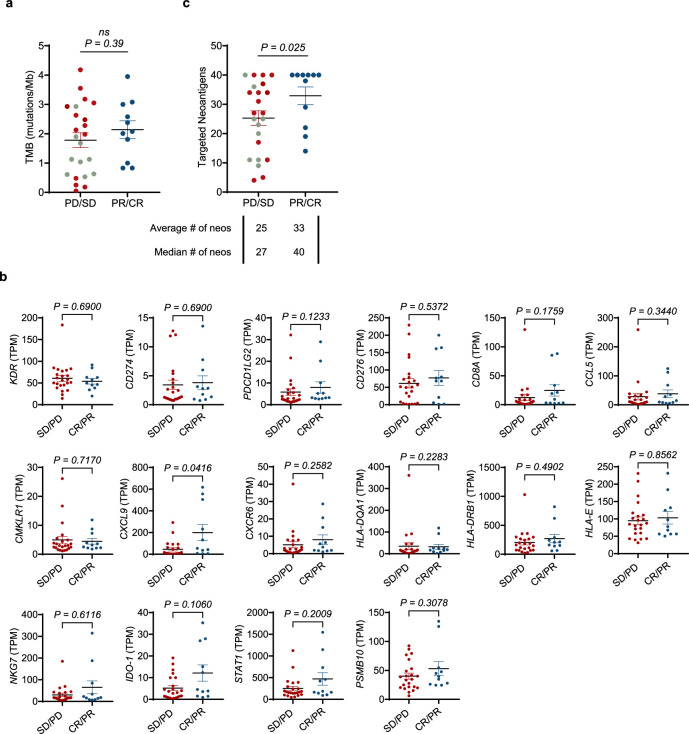
Observed clinical responses are associated with number of neoantigens included in the PTCV, but not known biomarkers of pembrolizumab response. **a**, Responses stratified by tumor mutational burden (TMB). **b**, Biomarkers of the T-cell inflamed gene expression profile (GEP) are evaluated at pre-treatment between available non-responders (SD/PD; n = 23) and responders (CR/PR; n = 11) RNA sequencing data (tumor biopsy). **c**, Association between clinical responses and the number of neoantigens included in the PTCV. SD/PD (n = 23) and CR/PR (n = 11) subjects were evaluated. Graphs indicate mean ± standard error of the mean (SEM) of data from individual subjects (circles). Data are presented as mean ± SEM. Significance was evaluated by a two-tailed (a, c), one-tailed (b), Mann-Whitney statistical analysis.

We next evaluated potential biomarkers of the observed clinical responses. All patients enrolled in GT-30 had a low TMB (fewer than five mutations per megabase), with a median TMB of 2.0 mutations per megabase. The TMB was similar in patients achieving CR/PR and those with SD/PD (Extended Data Fig. 5a). Similarly, while we observed a numerically higher ORR (35% versus 25%) in patients with baseline α-fetoprotein (AFP) levels of <400 ng ml^−1^ (*n* = 26) relative to patients with baseline AFP levels of >400 ng ml^−1^ (*n* = 8), there was no significant difference in disease control rate (58% versus 63%), mPFS (4.1 versus 6.2 months) or mOS (24.4 versus 15.6 months) (Table 1 and Supplementary Fig. 1). We next evaluated the relationship between baseline CD8 infiltration (assessed by the mRNA expression level of *CD8A*), pretreatment tumor *CD274* (PD-1 ligand 1) and *KDR* mRNA expression levels, and the T cell-inflamed gene expression profile of 15 biomarkers and the response to treatment (Extended Data Fig. 5b). These biomarkers broadly characterize an immune-inflamed phenotype and have previously shown potential utility in distinguishing responders from nonresponders to anti-PD-1-based therapies in HCC^30,31^. However, we did not observe any relationship between these pretreatment markers and the response to the PTCV plus anti-PD-1 therapy.

In contrast to the lack of differentiation based on pretreatment tumor biomarkers, an exploratory post hoc analysis demonstrated a positive correlation (*P* = 0.025) between the number of neoantigens included in the PTCV and the clinical response achieved. Sequencing of the patients’ tumors identified a median of 30 vaccine-targetable neoantigens (range 4–67). Among patients receiving a vaccine encoding ≥30 neoantigens, 7 of 17 (41.2%) had an objective response. Conversely, among those receiving a vaccine encoding <30 neoantigens, only 4 of 17 patients (23.5%) had an objective response. There was a significant difference in the number of targeted neoantigens between the CR/PR group and the SD/PD group (Extended Data Fig. 5c).

**Extended Data Fig. 6:**
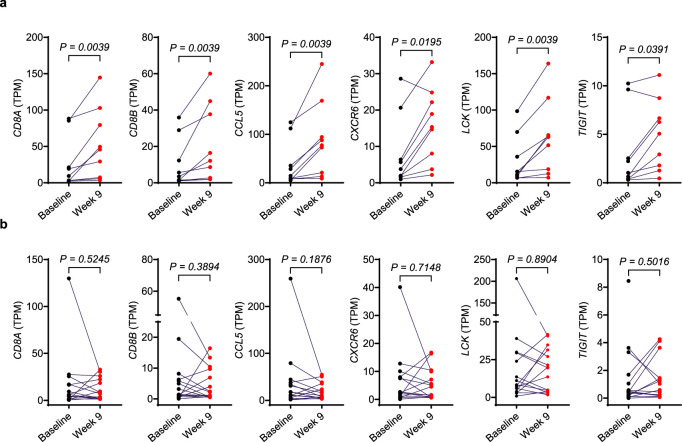
Change in T cell biomarker gene expression in the tumor upon PTCV treatment. T cell activation biomarkers are evaluated in pair-matched tumor biopsy samples from (a) responder (CR/PR; n = 9) and (b) non-responder (SD/PD; n = 15) patients treated with PTCV. Significance was tested by a two-tailed, Wilcoxon matched-pairs signed rank statistical analysis.

We next evaluated pretreatment versus on-treatment (week 9) tumor biopsy samples to determine whether changes in the expression levels of biomarkers associated with T cell activation and infiltration, consistent with a PTCV-mediated effect, could explain the observed clinical response in responders (CR/PR, *n* = 9) and nonresponders (SD/PD, *n* = 13). The expression of the T cell biomarkers *CD8A*, *CD8B*, *CCL5*, *CXCR6*, *LCK* and *TIGIT* was significantly increased in responders but not in nonresponders (Extended Data Fig. 6a,b). Although these analyses were exploratory, the results are broadly consistent with the proposed mechanism of action for PTCV-induced clinical responses. In contrast to anti-PD-1 monotherapy, which reinvigorates preexisting antitumor immunity, therapeutic cancer vaccines can prime new antitumor immune responses, providing a potential rationale for why inflamed and noninflamed tumors responded similarly to the therapy. Furthermore, the relationship between the number of neoantigens included in the PTCV and the clinical response achieved suggests that features of tumor antigenicity or of the vaccine itself drive clinical benefit with PTCV plus anti-PD-1 therapy.

### Vaccination elicits neoantigen-specific responses

In our study, predicted neoepitopes were selected for inclusion in the PTCVs using a pipeline for called variants based on an in silico analysis of the exome and transcriptome sequencing data from each patient (Methods). While the tumor RNA-sequencing (RNAseq) data confirmed that the nonsynonymous somatic variants included in the PTCVs were expressed, we did not experimentally confirm the processing and presentation of the predicted epitopes in the tumor.

**Fig. 3:**
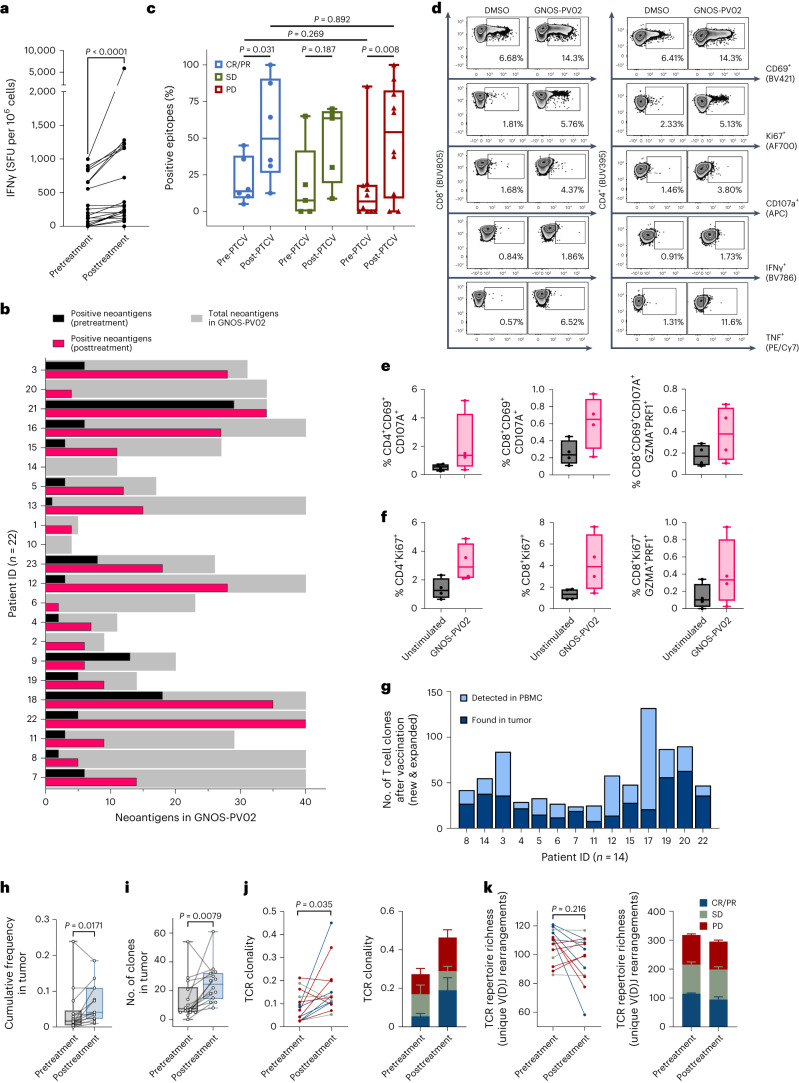
GNOS-PV02 drives polyfunctional antitumor neoantigen-specific T cell immunity. **a**, Vaccine-induced responses assessed by IFNγ ELISpot assays without cytokine stimulation (*n* = 22). Cumulative magnitudes were collected from positive epitopes before and after treatment. The postvaccination response is the ‘best’ (highest magnitude) response for each patient across time points. SFU, spot-forming units. **b**, Total neoantigens (gray bars) and positive neoantigens before (black bars) and after (red bars) vaccination in each patient’s PTCV assessed by IFNγ ELISpot. **c**, Percentage of positive responding epitopes by groups. The definition of a neoantigen-specific ELISpot response can be found in Methods. **d**, Representative density plots (patient 22) of the T cell markers CD69, Ki67, CD107a, IFNγ and TNF upon stimulation with patient-specific PTCV epitope pools. **e**,**f**, Polyfunctionality assessed by Boolean gating of CD4^+^ or CD8^+^ cytokine-producing populations. T cell activation (CD69 and CD107a; **e**) and proliferation (Ki67; **f**) were assessed together with the double-positive expression of GZMA and perforin 1 (PRF1) to evaluate the cytolytic potential of neoantigen-reactive T cells. Four patients (patients 7, 11, 18 and 22) were analyzed in **d**–**f**. **g**, T cell clones expanded in the periphery and the new or expanded clones enriched in the matched tumor sample for each patient (*n* = 14). Total PBMC and tumor-associated T cell expansion were calculated by comparing posttreatment to pretreatment PBMC or tumor samples (differential abundance statistical analysis). **h**, Cumulative frequencies of peripherally expanded TCR rearrangements in tumor biopsy samples. **i**, Expanded clone numbers in tumor biopsy samples. **j**,**k**, TCR clonality (**j**) and repertoire richness (**k**) in tumor biopsy samples (*n* = 14). PD (red), SD (gray), and CR/PR (blue). Error bars correspond to the upper s.e.m. of each group. Simpson clonality reports the distribution of TCR rearrangements in a sample, in which 0 indicates an even distribution of frequencies and 1 indicates an asymmetric distribution. TCR repertoire richness reports the mean number of unique rearrangements. Lower numbers indicate focused TCR diversity. Filled symbols in **c**, **e** and **f** and open circles in **h** and **i** represent individual patients; the box extends from the 25th to the 75th percentile; the line inside the box is the median; and the whiskers extend from the minimum to the maximum value. Significance between groups was evaluated by a two-tailed Mann–Whitney test (**c**); significance within groups was evaluated by a two-tailed Wilcoxon rank test (**a**, **h**–**k**).

Twenty-two patients with available peripheral blood mononuclear cell (PBMC) samples were evaluated for the presence of vaccine-induced neoantigen-specific responses before and after treatment using the ex vivo interferon-γ (IFNγ) enzyme-linked immunosorbent spot (ELISpot) assay. The criteria for ELISpot positivity are described in Methods. In almost all patients, treatment was associated with an increase in the magnitude of cumulative PTCV neoantigen-specific T cell responses (*P* < 0.0001) (Fig. 3a).

PTCV treatment was also associated with an increase in the number of encoded neoantigens eliciting an immune response. In 19 of 22 patients (86.4%), the number of vaccine-encoded neoantigens with T cell reactivity was higher after than before treatment (Fig. 3b). Two patients with PD, treated with PTCVs encoding 4 and 11 neoepitopes, did not yield any detectable ELISpot responses either before or after treatment; one patient with SD (20 neoantigens) had a reduced number of reactive epitopes detected after treatment relative to their pretreatment baseline. Individual epitope analyses across the cohort revealed PTCV encoded neoantigen-specific T cell responses to a median of 64.0% (range 0–100.0%) epitopes after treatment compared to 11.8% (range 0–85.3%) epitopes before treatment. PTCV immunization resulted in a significant increase in positive epitopes in both clinically responding and nonresponding patients (Fig. 3c).

**Extended Data Fig. 7:**
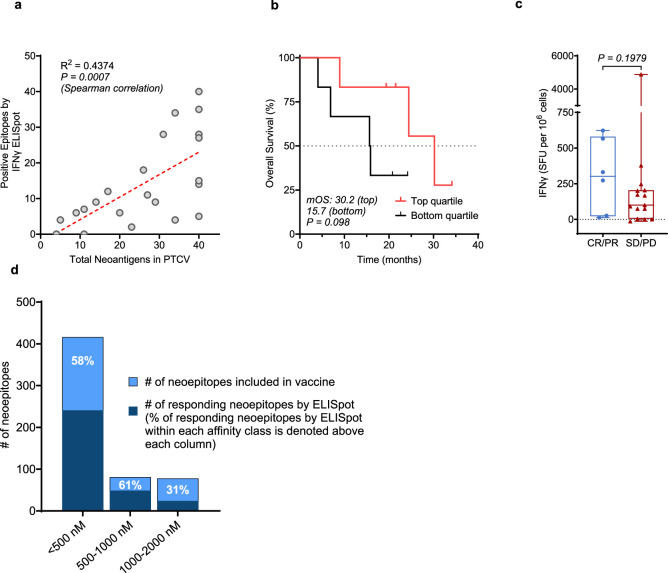
Association of clinical response with survival. **a**, A two-tailed, Spearman correlation between positive epitopes versus the total number of neoantigens included in each patient’s PTCV. **b**, Interquartile survival analysis by magnitude (best response post-PTCV; IFNγ SFU) of evaluable patients. Six patients per group; Log-rank (Mantel-Cox) test. **c**, T cell reactivity evaluated by IFNγ-ELISpot stratified by responder or non-responder groups. Symbols represent individual patients (CR/PR, n = 6; SD/PD, n = 15 per group), the box extends from the 25th to the 75th percentile, the line inside the box is the median, and the whiskers extend from the minimum to maximum values. Significance was tested by a two-tailed, Mann-Whitney statistical analysis. **d**, Cumulative number of targetable neoantigens encoded in the vaccines of the 22 pts whose samples were evaluated by IFNγ ELISpot assay. The neoantigens are stratified by the predicted MHC Class I binding affinity as high (< 500 nM), medium (500–1000nM), and low (1000–2000 nM). While the majority of the neoantigens encoded in the PTCVs were categorized as high-affinity antigens, the medium and low-affinity antigens also yielded positive responses in a similar proportion.

A positive correlation was observed between the total number of neoantigens included in the PTCV and the number of positive neoantigen responses detected by ELISpot assays (*P* = 0.0007, Spearman correlation coefficient) (Extended Data Fig. 7a). We evaluated the magnitude of IFNγ response by quartiles and observed that patients in the top quartile had a trend toward longer OS compared to patients in the bottom quartile (mOS 30.2 versus 15.7 months) (Extended Data Fig. 7b). Patients with CR or PR showed a trend toward a greater magnitude of IFNγ response (Extended Data Fig. 7c). Immune responses were observed against neoepitopes with predicted high binding affinity (*k*_d_ <500 nM), as well as against those with predicted medium or low binding affinity (*k*_d_ 500–2,000 nM), to human leukocyte antigen (HLA) class I molecules (Extended Data Fig. 7d).

Neoantigen-specific responses were confirmed in a subset of four responding patients (one with CR, three with PR) through intracellular staining of PBMCs stimulated with patient-specific neoepitope pools in vitro. Upon neoantigen stimulation, both CD4^+^ and CD8^+^ populations presented an increased activation profile as determined by the individual expression of the CD69, Ki67, CD107a, IFNγ and tumor necrosis factor (TNF) markers (Fig. 3d). Boolean gating confirmed an increasing trend of active (CD69^+^CD107a^+^) (Fig. 3e) and proliferative (Ki67^+^) (Fig. 3f) polyfunctional CD4^+^ and CD8^+^ T cells with cytolytic capabilities (GZMA^+^PRF1^+^) after stimulation. Taken together, these data indicate that vaccination is capable of eliciting polyfunctional neoantigen-specific CD4^+^ and CD8^+^ responses with cytolytic potential. We next characterized T cell clonal expansion, trafficking, neoantigen specificity, and clonal and subclonal genetic profiles as exploratory endpoints.

### Vaccination enriches T cell clone expansion and infiltration

Complementarity-determining region 3 (CDR3) regions of the T cell receptor β-chain (TCRβ) were sequenced from paired pretreatment and posttreatment (weeks 9–12) PBMC and tumor biopsy samples in 14 patients with available paired tumor biopsy samples. Although anti-PD-1 therapy is not known to modulate the diversity of tumor-reactive T cell clones^32,33^, we hypothesized that the addition of the PTCV to anti-PD-1 therapy would lead to both an increase in abundance and a broadening of the circulating HCC-reactive T cell clonal repertoire, which would subsequently traffic to the tumor microenvironment. Consistent with this hypothesis, we observed significant T cell clonal expansion in 14 of 14 (100%) patients in both the peripheral blood and tumor using a differential abundance statistical framework (Fig. 3g). The median number of new or expanded T cell clones in the periphery was 47 (range 24–132), of which a median of 21 (range 6–71) T cell clones were also new or expanded in the posttreatment tumor. The median increase in the cumulative frequencies of the significantly expanded clones was 1.94% (range 0.35–8.70%) (Supplementary Fig. 2a,b). We identified an increase in both the abundance and number of expanded T cell clones within the tumor after treatment, which was also identified in the peripheral blood after treatment (Fig. 3h,i). Importantly, we observed higher frequencies and numbers of T cell clones newly present in the tumor after vaccination (Supplementary Fig. 3a,b). Additionally, we found significantly increased TCR clonality (*P* = 0.035) (Fig. 3j) but no significant change in TCR repertoire richness in the tumor (*P* = 0.216) (Fig. 3k). These data suggest that therapy with PTCV results in the expansion of T cells in the periphery, with T cells trafficking to the tumor.

### Vaccination drives effector T cell memory clonal expansion

**Extended Data Fig. 8:**
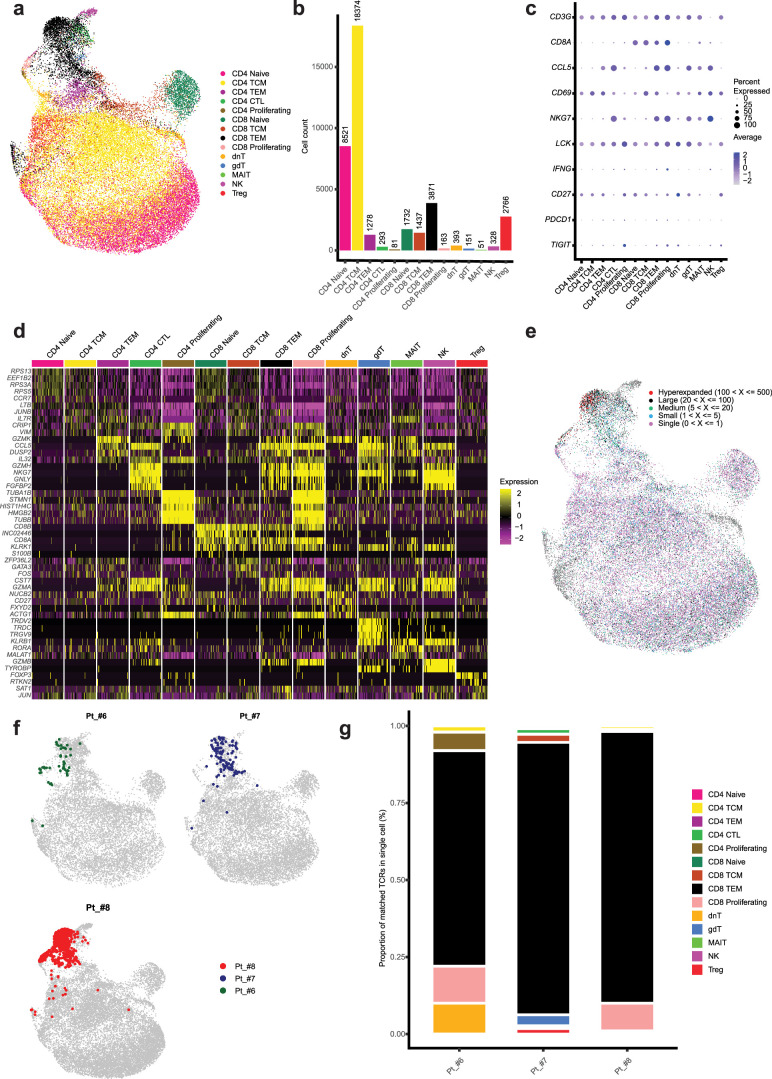
Peripheral blood T cell responses to PTCV are primarily driven by clonal expansion of T cell effector memory cells clusters. **a**, Uniform Manifold Approximation and Projection (UMAP) of single-cell transcriptomes of 39,435 T cells from peripheral blood samples (n = 4) obtained at 12 weeks post-vaccination from 3 patients (#6, #7, and #8), colored by cluster. **b**, Barplot showing the number of cells occupying each cluster. **c**, Dotplot showing gene expression CD3E, CD3G, CD8A, CD8B, CCL5, IDO1, CD69, NKG7, LCK, IFNG, CXCR6, CD27, PDCD1, and TIGIT. Size of the dot indicates the percent expression among cells assigned to each cluster and the color represents the average expression across all cells within the cluster. **d**, Heatmaps showing scaled expression of the top 5 marker genes identified for each subset. **e**, UMAP indicating areas of clonal expansion within the 31,842 cells identified as having a TCR by paired single-cell TCR-sequencing. Colors indicate the degree of clonal expansion of each TCR clonotype. **f**, UMAPs indicating single cells with a TCRβ identified as clonally expanded after vaccination by bulk TCRβ sequencing of pre- and post-treatment peripheral blood, by patient. **g**, Transcriptional phenotype of cells with a TCRβ identified as clonally expanded by bulk TCR sequencing of pre- and post-treatment peripheral blood, represented as a percentage of total number of single cells per patient.

To characterize the vaccine-induced T cell response further, we performed single-cell RNAseq/TCR sequencing (TCRseq) of peripheral blood at the 12-week postvaccination time point. In four samples obtained from three patients (patients 6 (SD), 7 (CR) and 8 (PR)), we assigned PBMCs to 14 clusters based on transfer learning from reference PBMC data and analysis of canonical marker genes (Extended Data Fig. 8a–d)^34–36^. Clusters demonstrating the highest expression of genes associated with cytotoxicity, including chemokine C–C motif ligand 5 (*CCL5*) and granzyme K (*GZMK*), *GZMB* or granulysin (*GNLY*), were CD4^+^ effector memory T (T_EM_), CD4^+^ cytotoxic T lymphocyte (CTL), CD8^+^ T_EM_ and CD8^+^ proliferating cells, as well as γδ T and natural killer (NK) cells. In 31,843 of 39,439 cells, a TCR sequence was identified by paired single-cell immune repertoire sequencing. Across all single-cell clusters with an associated TCR, clonally expanded T cell populations, which we defined as more than five cells that shared the same TCR, were most strongly associated with the CD8^+^ proliferating (odds ratio, 5.84; 95% CI, 4.07 to 8.43; *P* < 0.001), CD8^+^ T_EM_ (odds ratio, 5.58; 95% CI, 5.15 to 6.06; *P* < 0.001) and CD4^+^ CTL (odds ratio, 4.14; 95% CI, 3.13 to 5.48; *P* < 0.001) clusters by Fisher’s exact test (Extended Data Fig. 8e).

For each patient, we identified all T cells in the single-cell dataset with a TCRβ that had been identified as clonally expanded in bulk TCRseq of prevaccination and postvaccination peripheral blood (Extended Data Fig. 8f). Of 92 TCRβ sequences found to be clonally expanded by bulk sequencing, 64 sequences were identified in 1,041 cells within the single-cell dataset, of which 84.4% (879 of 1,041) were from patient 8, 10.8% (112 of 1,041) were from patient 7 and 4.8% (50 of 1,041) were from patient 6. The single-cell cluster most strongly associated with vaccine expansion by Fisher’s exact test was CD8^+^ T_EM_ (odds ratio, 107.40; 95% CI, 88.75 to 130.91; *P* < 0.001), which comprised 87.3% (909 of 1,041) of vaccine-expanded TCRβ (Extended Data Fig. 8g and Supplementary Fig. 4a). On a per-patient basis, CD8^+^ T_EM_ was also the most highly represented cluster, being found in 88% (775 of 879), 88% (99 of 112) and 70% (35 of 50) of vaccine-expanded TCRβ for patients 8, 7 and 6, respectively.

**Extended Data Fig. 9:**
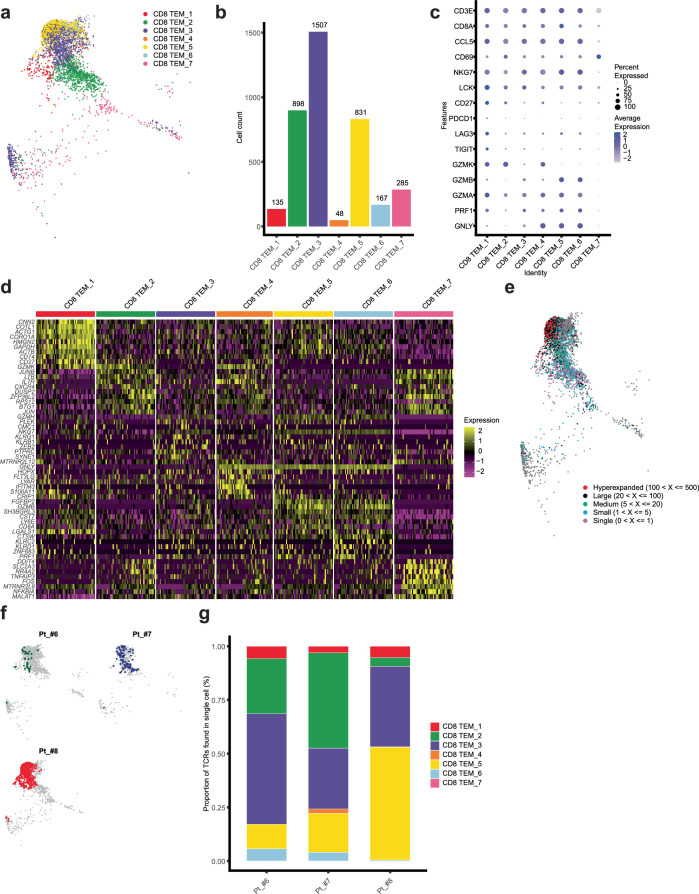
Subcluster analysis of CD8 TEM cluster in single cell sequencing of peripheral blood T cells at week 12 post-vaccination. **a**, Uniform Manifold Approximation and Projection (UMAP) of single-cell transcriptomes of 3861 CD8 TEM cells from peripheral blood samples (n = 4) obtained at 12 weeks post-vaccination from 3 patients (#6, #7, and #8), colored by cluster. **b**, Barplot showing the number of cells occupying each CD8 TEM subcluster. **c**, Dot plot showing gene expression CD3E, CD8A, CCL5, CD69, NKG7, LCK, CD27, PDCD1, LAG3, TIGIT, GZMK, GZMB, GZMA, PRF1, and GNLY. across CD8 TEM subclusters. **d**, Heatmaps showing scaled expression of the top 10 marker genes identified for each subset. **e**, UMAP indicating areas of clonal expansion in 2,738 cells identified as having a TCR by paired single-cell TCR-sequencing. Colors indicate the degree of clonal expansion of each TCR clonotype. **f**, UMAPs indicating single cells with a TCRβ matching clonally expanded TCRs identified by bulk sequencing of pre- and post-treatment peripheral blood, divided by patient. **g**, CD8 TEM subcluster identities of single cells with a TCRB matching clonally expanded TCRs identified by bulk sequencing of pre- and post-treatment peripheral blood, represented as percentage of total number of single cells per patient.

We then subdivided the CD8^+^ T_EM_ cluster into subclusters and identified seven subclusters, among which three (CD8^+^ T_EM__3, CD8^+^ T_EM__5 and CD8^+^ T_EM__6) displayed high expression of multiple genes associated with cytotoxicity, including *GZMB* and *NKG7*, and three (CD8^+^ T_EM__1, CD8^+^ T_EM__2 and CD8^+^ T_EM__4) had increased expression of *GZMK* (a preexhaustion marker)^37^ (Extended Data Fig. 9). Cytotoxic subclusters accounted for 85% (776 of 901) of vaccine-expanded CD8^+^ T_EM_, with CD8^+^ T_EM__5 and CD8^+^ T_EM__3, which comprised 47.2% (429 of 909) and 37.1% (337 of 909) of CD8^+^ T_EM_, respectively, being the two largest clusters represented. In contrast, the GZMK-expressing preexhausted subclusters CD8^+^ T_EM__2, CD8^+^ T_EM__1 and CD8^+^ T_EM__4 were less numerous (Supplementary Fig. 4b).

### Expanded TCR clones are reactive to PTCV-encoded antigens

**Fig. 4:**
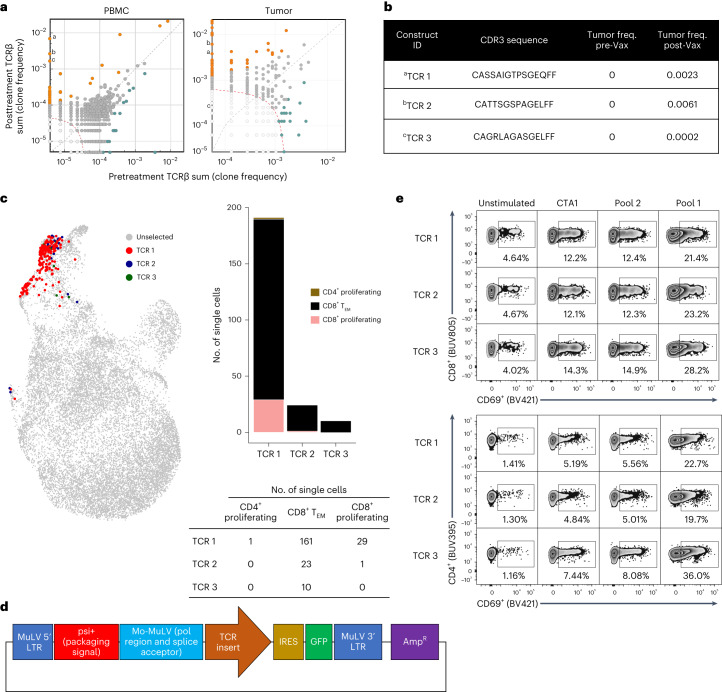
Postvaccination expanded TCR clones identified in the tumor are reactive to PTCV-encoded antigens. **a**, Most frequent TCRs identified by TCRseq and RNAseq in a patient (before vaccination versus week 9 after vaccination, pairwise scatterplots). Different superscript letters show selected high-frequency new T cell clones detected in PBMCs after vaccination and their abundance in the tumor. Orange, green, and gray circles represent expanded, contracted and not significantly changed T cell clones, respectively. **b**, CDR3 sequences of the three TCRs (from patient 8; TCR 1, TCR 2 and TCR 3) selected for cloning and their frequency (freq.) in the tumor before (pre-Vax) and after (post-Vax) vaccination. Selected cloned TCRs were present in high frequency only in the peripheral blood and tracked into the tumor after treatment. **c**, UMAP (Uniform Manifold Approximation and Projection) and stacked barplot indicating the single-cell cluster identities and number of cells for each of the three TCRs selected for cloning. **d**, Patient-specific clonal TCR sequences were gene optimized and inserted into the pMXs-IRES-GFP retroviral plasmid vector containing the viral packaging signal, transcriptional and processing elements, and the *GFP* reporter gene. MuLV, murine leukemia virus; Mo-MuLV, Moloney MuLV; LTR, long terminal repeat; Amp^R^, ampicillin resistance. **e**, TCR-engineered T cells (GFP^+^) from unvaccinated patient-derived PBMCs were stimulated for 6 h with epitope pools or the nonspecific epitope CTA1 (10 µg ml^−1^), and CD69 expression was evaluated by flow cytometry. Peptide pool 1 included the most reactive epitopes measured by ELISpot, whereas pool 2 (consisting of peptides corresponding to epitopes 21–40) served as an internal negative control.

Lastly, we sought to validate the neoantigen-specific activity of tumor-infiltrating T cells in two representative patients. The first patient had 42 significantly expanded clones in the periphery, of which 27 were found in the tumor sample after treatment (Figs. 3g and 4a). Three of the most frequent TCR sequences (Fig. 4b) from T cell clones newly present in the tumor after vaccination were found primarily in the CD8^+^ T_EM_ cluster in postvaccination peripheral blood single-cell sequencing (Fig. 4c). These three TCR sequences were selected and cloned into the pMXs-IRES (internal ribosome entry site)-GFP (green fluorescent protein) retroviral plasmid vector for further studies (Fig. 4d). To characterize the neoantigen-specific cellular response driven by treatment with GNOS-PV02, we stimulated TCR-engineered T cells from patient-derived PBMCs with the patient’s PTCV-specific neoantigen pools. We found T cell activation (CD69^+^) associated with pool 1 (consisting of peptides corresponding to neoantigens 1–20), which included the most reactive epitopes measured by ELISpot; pool 2 (consisting of peptides corresponding to neoantigens 21–40) served as an internal control for specificity and showed similar levels as the nonspecific epitope (CTA1) control (Fig. 4e). In the second representative patient, we were able to map the new T cells/TCRs to a specific vaccine-encoded epitope. From IFNγ ELISpot analysis, we first identified a strongly immunogenic epitope (ATP1A1-ALB) encoded in the patient’s personalized vaccine (Supplementary Fig. 5a). Patient-derived PBMCs were subjected to in vitro stimulation for T cell enrichment and expansion and then stimulated with ATP1A1-ALB peptides. We found both CD4^+^ and CD8^+^ T cells with specific polyfunctional responses (CD69^+^, Ki67^+^, CD137^+^, IFNγ^+^, IL-2^+^) against ATP1A1-ALB (Supplementary Fig. 5b). High-frequency TCRs were identified by TCRseq/RNAseq (33 clones expanded in the periphery, of which 15 were found in the tumor) and engineered (Supplementary Fig. 5c,d). Engineered TCRs were stimulated with a pool of epitopes containing all the neoantigens in the patient’s PTCV. Similar to the first patient, we observed CD4^+^ and CD8^+^ T cell specificity against the patient-specific neoantigens (Supplementary Fig. 5e) relative to the unstimulated or nonspecific peptide (CTA1)-stimulated controls. These data validate the postvaccination infiltration and increase in the frequency of T cells in the tumor with specificity to vaccine-encoded neoantigens.

### Vaccine-induced immune editing leads to tumor escape

**Extended Data Fig. 10:**
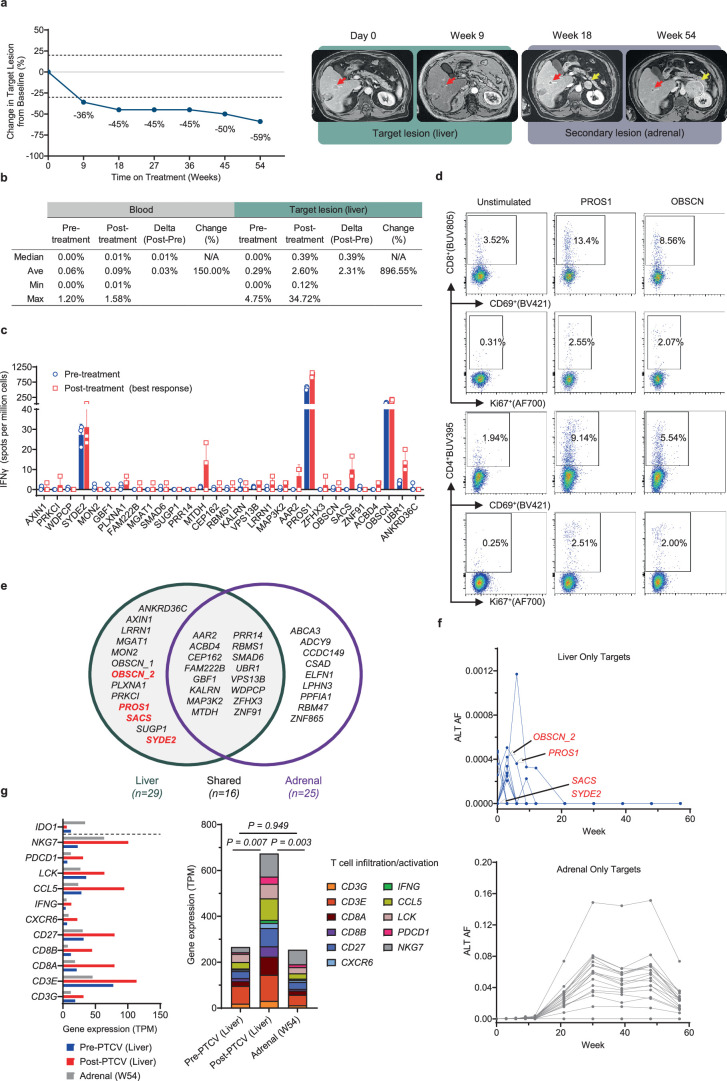
Patient #11 – Case Study. **a**, Change in target lesion from baseline (%) evaluated by RECIST 1.1 and tumor imaging scans at various treatment time points. Liver lesion (red arrows); Adrenal lesion (yellow arrows). **b**, TCR frequencies (%) of 25 expanded T cell clones at pre- or post-treatment in the periphery or in the target lesion (liver). **c**, PBMCs (3 × 10^5/per well) were stimulated with vaccine-encoded epitopes at the concentration of 10 μg/mL for 18–24 hours. Cells were evaluated for the presence of vaccine-induced neoantigen-specific responses prior to and post-personalized GNOS-PV02 vaccination using an interferon IFNγ ELISPOT assay without cytokine stimulation. The bar indicates the mean SFU of n = 3 individual technical replicates ± SD per group. **d**, Neoantigen-specific T cell activation was evaluated by stimulating patient-derived PBMCs (week 9) with DMSO, PROS1, or OBSCN peptides ex vivo by intracellular cytokine staining. **e**, Venn diagram of neoantigens identified in the liver (day 0) and adrenal lesions (week 54) by RNA/DNA sequencing. **f**, Monitoring of the dynamic expression of liver- or adrenal-specific targets collected over 50 weeks by ctDNA analysis. **g**, T cell infiltration/activation biomarkers are evaluated at pre-treatment and post-treatment (liver lesion), and from the adrenal lesion (week 54). The T cell suppressor, IDO1, is separated from the other markers with a dashed line. The significance between evaluated stacked gene expression groups was tested by a two-tailed, Mann-Whitney statistical analysis.

Tumor immune editing and escape are key mechanisms of cancer progression and metastatic dissemination. We used paired tumor biopsy samples to investigate the mechanisms of tumor escape in a patient with nondurable PR (patient 11) treated with a PTCV encoding 29 neoantigens plus pembrolizumab. At the first restaging interval at week 9, the patient had a target liver lesion reduction of −36%. By week 18, the response in the target liver lesion intensified, eventually reaching a −59% reduction, but a new lesion was observed in the adrenal gland (Extended Data Fig. 10a).

TCR/tumor-infiltrating lymphocyte (TIL) analysis of week 9 versus screening biopsy samples revealed the clonal expansion and infiltration frequencies of 25 T cell clones after vaccination (Extended Data Fig. 10b). IFNγ ELISpot analysis detected T cell responses to 9 of 29 epitopes and strong responses to 4 of 29 vaccine epitopes (Extended Data Fig. 10c), none of which were recognizable as driver mutations. Flow cytometry analysis showed a high frequency of antigen-specific, activated (CD69^+^, Ki67^+^) CD8^+^ and CD4^+^ T cells (Extended Data Fig. 10d). Sequencing of the new adrenal lesion identified 25 neoantigens, including 16 shared with the primary liver lesion (Extended Data Fig. 10e). However, all four vaccine neoantigens with the strongest ELISpot responses were absent in the adrenal lesion, consistent with neoantigen loss resulting from immune editing and clonal escape. The ctDNA analysis results were consistent with CR of the liver-specific tumor clones by week 21, persisting through week 57, but showed an increasing level of the adrenal-specific ctDNA over time (Extended Data Fig. 10f). Tumor tissue biomarker analysis of week 0 versus week 9 liver tumor samples showed robust CD8^+^ T cell infiltration, whereas the adrenal lesion had low CD8^+^ T cell density resembling that of the pretreatment liver tumor (Extended Data Fig. 10g). These data are consistent with tumor immune escape in the setting of tumor heterogeneity and the loss of passenger neoantigens targeted by the PTCV, which supports the proposed mechanism of action of PTCV-mediated antitumor immunity.

## Discussion

HCC is characterized by a modest response to anti-PD-1 monotherapy. New personalized immunotherapies that induce tumor-specific T cell responses may sensitize tumors to ICI therapy^11,12,14,38–40^. We show that treatment with a PTCV containing up to 40 tumor neoantigens in combination with pembrolizumab is feasible and associated with clinical responses in a subset of patients with advanced HCC. Although the single-arm design and small sample size of the current study limit the ability to attribute clinical efficacy to the PTCV definitively, the observed response rate (30.6%) is higher than that in historical clinical trials of anti-PD-1 monotherapy in HCC (12–18%)^4–10^.

Immunological analysis confirmed both the induction of new T cell responses to vaccine-encoded antigens and an expansion of the TCR repertoire in both the peripheral blood and tumor. Anti-PD-1 monotherapy can reverse T cell dysfunction in existing neoantigen-specific T cell clones but is not known to induce new neoantigen-specific T cell clones^32,33^. Therefore, the present study provides evidence that a PTCV can enhance responses to anti-PD-1 therapy through the induction of neoantigen-specific T cells in the peripheral blood and tumor. Single-cell sequencing analysis showed that the PTCV-expanded T cells clustered predominantly with the CD8^+^ T_EM_ populations, followed by CD8^+^ proliferating populations, and displayed high expression of markers consistent with a cytotoxic (GZMB, NKG7) phenotype and, less frequently, with a preexhausted (GZMK) phenotype. These immunologic data support a PTCV’s contribution to anti-PD-1 efficacy.

The present trial builds upon recent clinical updates with neoantigen-specific cancer vaccines across a variety of tumor types and vaccine platforms. One notable difference between the vaccine platform used in the present study and other neoantigen vaccine platforms is that other vaccine platforms have generally targeted a more limited selection of neoantigens. Our vaccination approach aimed to include all targetable neoantigens in each patient’s vaccine—drivers and passengers, truncal and branch, shared and private, and with a broader range of predicted binding affinity to major histocompatibility complex molecules. We reasoned that vaccines that encode for a larger repertoire of tumor-derived neoantigens may lead to the priming of a broader set of immune responses, increasing the likelihood of effective tumor control. We observed that an increased number of epitopes encoded in the PTCV correlated with an increased number of reactive epitopes after vaccination. We detected de novo responses not only to predicted high-affinity epitopes but also to medium- and low-affinity epitopes at similar rates. Collectively, these data are consistent with preclinical findings^18,24^ that contemporary vaccines can prime immune responses to a broader range of neoantigens than previously envisioned.

A recent study of an mRNA-based neoantigen vaccine (autogene cevumeran) in the adjuvant treatment of pancreatic ductal adenocarcinoma showed that patients with a vaccine response (50% of all patients) achieved improved recurrence-free survival compared to those who did not mount a T cell response to the vaccine neoantigens^41^. We also observed a trend toward longer OS among patients who had a stronger vaccine-driven response relative to those who had a weaker response. However, in our study, 90.1% of the evaluated patients (20 of 22) had neoantigen-specific T cells detected by IFNγ ELISpot assays, limiting comparisons to only *n* = 2 patients who did not have such responses. Our on-treatment PBMC and tumor tissue biomarker data showed that patients with a clinical response of CR or PR demonstrated increased levels of T cell activity markers relative to baseline. Thus, other factors such as the tumor microenvironment and immune fitness may have a confounding role in ultimately driving the clinical response^42^. Our analysis of a single patient with early acquired resistance to therapy in the GT-30 study provides initial support for neoantigen loss and tumor heterogeneity as important barriers to PTCV therapy. We envision that, in the future, longitudinal sampling of tumor DNA from multiple lesions coupled with rapidly synthesized therapeutic vaccines with evolved neoantigen panels can be used to address tumor heterogeneity and control newly unresponsive lesions.

The major limitations of the present study include the small sample size and the single-arm study design. Although the combination of a PTCV and pembrolizumab met the prespecified endpoint for clinical response rate as compared to a historical control of pembrolizumab monotherapy, we cannot exclude the possibility that study population differences affected the response rates observed in the present study. Prospective, randomized clinical trials are needed to confirm that the PTCV can improve clinical outcomes as compared to pembrolizumab alone. An additional limitation is that this study was conducted in the setting of a rapidly evolving HCC clinical landscape, limiting the clinical applicability of these findings. Since the initiation of the present study, multiple ICI combinations have demonstrated improved survival benefits versus mTKI therapy in patients with advanced HCC in the first-line setting (including bevacizumab plus atezolizumab^43^, durvalumab plus tremelimumab^27^, and camrelizumab plus rivoceranib^44^), with similar response rates as were observed in the present study. Additionally, new triplet regimens are under investigation and may prove superior to current therapeutic options. However, many patients are not candidates for bevacizumab-based combinations, and a particular challenge with anti-vascular endothelial growth factor-sparing combinations in HCC is the potential for irAEs. For example, the combination of ipilimumab (an anti-CTL-associated antigen 4 antibody) and nivolumab (an anti-PD-1 antibody) is approved with a 32% response rate in second-line HCC treatment^45^, which is similar to what was observed in the present study; however, more than half of the patients experienced serious irAEs. Therefore, there is a particular need for new immunotherapies that can enhance anti-PD-1 responses without increasing the risk of irAEs. Except for an increase in injection-site reactions, the overall safety profile of the PTCV plus anti-PD-1 combination was similar to that of pembrolizumab alone despite driving tumor-directed TILs and clinical responses. Therefore, a PTCV could eventually be integrated into the current armamentarium of effective systemic therapies for HCC. In summary, our data indicate that the development of PTCVs is feasible and can induce clinical responses in combination with anti-PD-1 therapy in advanced HCC.

## Methods

### Trial design and treatment plan

We conducted a 36-patient, phase 1/2, multicenter, open-label trial of a PTCV (GNOS-PV02 and pIL12) plus pembrolizumab in patients with advanced HCC who progressed or were intolerant to first-line therapy with an mTKI. The individualized patient demographic information is shown in Supplementary Table 1. Methods for tumor sequencing and PTCV manufacturing are described below. All selected neoantigens (up to 40) were assembled into a single patient-specific vaccine plasmid (GNOS-PV02) and manufactured for each patient while they were receiving first-line systemic therapy. The PTCV (GNOS-PV02 (1 mg) and pIL12 (0.34 mg)) was administered intradermally using a CELLECTRA 2000 electroporation device (INOVIO Pharmaceuticals) into two locations (deltoid area of both arms) Q3w for four doses, followed by Q9w until year 2 and Q12w thereafter. Pembrolizumab was administered at the standard dose of 200 mg intravenously Q3w for up to 2 years per label recommendation. Therapy was continued until the progression of disease, development of unacceptable toxicity, withdrawal of consent or end of the study.

Eligibility criteria included age ≥18 years, a confirmed diagnosis of HCC, BCLC stage B or C disease, Child–Pugh class A, a predicted life expectancy of >6 months, a performance status of 0 or 1 using the ECOG performance scale, and measurable disease based on RECIST 1.1. Key exclusion criteria were the use of prior systemic therapy for HCC other than sorafenib or lenvatinib and active autoimmune disease. The full eligibility and exclusion criteria are provided in the study protocol (Supplementary Information).

### Study oversight

The protocol of the GT-30 clinical study was approved by the institutional review board or ethics committees at each participating institution. Written informed consent was obtained from each patient. The study was registered at https://clinicaltrials.gov/ under the identifier NCT04251117. The trial was conducted in accordance with the principles of the Declaration of Helsinki. Data were collected by the study investigators and analyzed by employees of Geneos Therapeutics.

### Endpoints

The primary endpoint was safety, graded using the Common Terminology Criteria for Adverse Events version 5.0. The coprimary endpoint was immune response, assessed by quantifying IFNγ-secreting T lymphocytes in PBMCs by ELISpot. Secondary endpoints included ORR according to RECIST 1.1, PFS and OS. Response data were provided by local sites and were not centrally reviewed. This analysis included all patients enrolled in the cohorts who received at least one dose of the PTCV. The exploratory endpoints included the evaluation of tumor and immune biomarkers and their association with the treatment outcome. The data cutoff date was August 18, 2023.

### PTCV design

Next-generation sequencing of patient-specific tumor samples was performed using the ACE technology (Personalis). DNA samples from matched normal tissue were also prepared for germline whole-exome sequencing. Sequence alignments, variant discovery and annotation, and comprehensive analysis were performed to identify all targetable neoantigens for vaccine design. Targetable neoantigens were identified based on somatic, nonsynonymous nucleotide changes (single-nucleotide variants, indels, fusions) with a DNA allelic fraction of >0.05 and RNAseq FPKM (fragments per kilobase of transcript per million mapped reads) of >1. The resulting peptides were filtered for duplicates and self-similarity and ranked by HLA class I-binding affinity (nM, high to low, NetMHCpan 4.0). All targetable neoantigens (up to 40) were included in each patient’s PTCV. Where >40 potential neoantigens were identified, the 40 neoantigens with the strongest HLA-binding affinity (lowest nM *k*_d_) were included in the PTCV. Patient-specific DNA vaccine constructs were designed to consist of a string of epitopes with flanking sequences with a center-embedded CD8 epitope separated by synthetic furin cleavage sites for efficient epitope presentation during processing^18,24^. Each neoepitope contained the somatic variant and its flanking sequence so that each is about 33 amino acids in length. The final assembled cassettes were codon and RNA optimized, synthesized and subcloned into the vaccine expression vector pGX0001 (GenScript). Details of the PTCV design and vaccine plasmid construction are shown in Supplementary Fig. 6. IL-12 DNA consisted of a single plasmid containing a dual-promoter system for the expression of both the IL-12 *p35* and *p40* genes necessary for the production of the active heterodimeric IL-12 protein^25,46^. All plasmids were sequence verified and manufactured under current good manufacturing practice conditions (VGXI) and met all acceptance criteria for release.

### Blood collection and PBMC isolation for immunology assessments

Whole blood samples were collected before treatment, Q3w after treatment until week 12, and then Q9w for immunological analyses. PBMCs from whole blood were isolated, counted and cryopreserved in CryoStor CS10 (part no. 210102, STEMCELL Technologies) according to standardized protocols. Cryopreserved cells were thawed, washed, counted and rested overnight before use in immunological assays.

### Peptides

Custom-made, recombinant, lyophilized peptides specific to each patient were produced (GenScript). Peptides were reconstituted at 100 mg ml^−1^ per peptide in sterile dimethylsulfoxide (DMSO) (cat. no. BDH1115-1LP, VWR International), aliquoted and stored at −80 °C. A single-neoepitope peptide pool (1 mg ml^−1^) consisted of four peptides covering the entire 33-mer neoepitope, each containing 15 amino acids, overlapping by eight amino acids. Additionally, a 9-mer predicted CD8 epitope was synthesized and included in the single-neoepitope pool. To make two large peptide pools covering all vaccine-encoded patient-specific neoepitopes, we pooled (at a concentration of 1 mg ml^−1^) all peptides covering the first half of the vaccine-encoded neoepitopes into pool 1 and pooled (at a concentration of 1 mg ml^−1^) the remaining peptides covering the second half of the vaccine-encoded neoepitopes into pool 2. The B*40:01-restricted NY-ESO-1 epitope, EFTVSGNIL (CTA1), was used as a nonspecific stimulus at 10 µg ml^−1^.

### IFNγ ELISpot assay

The ELISpot assay was performed (FlowMetric) using the standard ELISpot protocol^47^ and the human IFNγ single-color ELISpot kit (all reagents and plates included; stock keeping unit no. hIFNgp-1M, Cellular Technology Limited). Briefly, PBMCs from each patient (3 × 10^5^ cells per well) were placed in ELISpot multiscreen plates precoated with antihuman IFNγ capture antibody, stimulated with the matching peptide pools (single-neoepitope pool or larger pools with multiple neoepitopes) at a concentration of 10 μg ml^−1^ for 18–24 h. No cytokine stimulation was performed. An equivalent amount of DMSO was added to control wells. PBMCs from each patient were set in duplicate or triplicate for peptide stimulations and controls. After stimulation, cells were removed and a biotinylated secondary antibody was added. After 2 h of incubation, the plates were washed, added with streptavidin-conjugated alkaline phosphatase and further incubated for 1 h. The plates were developed by adding 5-bromo-4-chloro-3-indolyl phosphate/nitroblue tetrazolium as the substrate. ELISpot plates were analyzed on the CTL ImmunoSpot S6 Ultimate-V analyzer (Cellular Technology Limited) using ImmunoSpot software version 5.1. The cell viability and counts upon thawing of the PBMC vials are reported in Supplementary Fig. 7.

A T cell response to a specific epitope at a time point was considered positive if it met each of the three criteria to assure with 95% confidence that the response could be attributed to the specific peptide. The epitope-specific response had to be (1) at least 2 s.d. above the corresponding unstimulated control sample (background), (2) at least twofold above the corresponding unstimulated control sample (background) and (3) at least 5 SFU. The same criteria were used to evaluate pretreatment samples (for preexisting neoepitope responses) and on-treatment samples. Data are presented as SFU per 10^6^ PBMCs.

For the calculation of the number and percentage of responding epitopes, an epitope was counted if it resulted in a positive ELISpot response, as defined above, at any time point.

For the analysis of the magnitude of response, upon determination of a positive response, each sample was background corrected by subtracting the average value of the negative control peptide wells. The background-subtracted responses of each positive epitope were summed for the pretreatment baseline and for each available on-treatment time point to determine the cumulative ELISpot response for that time point. The postvaccination response for each patient is the ‘best’ cumulative response (highest magnitude) for that patient across the available time points.

### In vitro stimulation and intracellular staining

The patients’ PBMCs (2.5 × 10^5^ cells) were cultured in a growth medium (RPMI with 10% FBS) supplemented with a cocktail of the IL-2 (20 IU ml^−1^), IL-4 (10 ng ml^−1^) and IL-7 (10 ng ml^−1^) cytokines and enriched for neoantigen-specific T cells using 10 µg ml^−1^ of epitope stimuli. Three days later, cells were washed and the supplemented growth medium was replaced. On day 4, epitope stimuli (10 µg ml^−1^) or controls were added, followed by incubation for 1 h. Then, anti-CD107a-APC (clone H4A3, BioLegend) antibody and Protein Transport Inhibitor Cocktail (1:500 dilution, Invitrogen) were added. After a 5-h incubation, cells were stained using fluorescently labeled surface marker antibodies: anti-CD3-BV711 (clone UCHT1, BD Biosciences), anti-CD4-BUV395 (clone RPA-T4, BD Biosciences), anti-CD8-BUV805 (clone RPA-T8, BD Biosciences), anti-CD69-BV421 (clone FN50, BD Biosciences), anti-CD137-BV605 (clone 4B4-1, BioLegend) and dump markers including anti-CD14/-CD16/-CD19-APC-H7 (clone MΦP9, 3G8, SJ25CI, respectively) (all from BD Biosciences). Dead cells were stained using Live/Dead Blue solution (1:1,000 dilution, Thermo Fisher Scientific), followed by overnight fixation and permeabilization using fixation/permeabilization buffers (cat. nos. 00-5123-43 and 00-5223-56, eBioscience) according to the manufacturer’s instructions. Cells were then stained intracellularly in eBioscience permeabilization buffer (cat. no. 00-8333-56) with anti-IFNγ-BV786 (clone 4S.B3, BD Biosciences), anti-IL-2-FITC (clone MQ1-17H12, BD Biosciences), anti-Ki67-AF700 (clone B56, BD Biosciences), anti-GZMA-PerCP/Cy5.5 (clone CB9, BioLegend), anti-perforin-PE/Dazzle 594 (clone dG9, BioLegend) and anti-TNF-PE/Cy7 (clone MAb11, BD Biosciences) antibodies. The gating strategy is shown in Supplementary Fig. 8. Flow cytometry data were acquired on the LSRFortessa analyzer (BD Biosciences) using FACSDiva software version 8.0.1 and analyzed using FlowJo version 10.4 or later.

### TCR variable β-chain sequencing

Immunosequencing of the CDR3 regions of human TCRβ chains was performed using the immunoSEQ assay (Adaptive Biotechnologies). Extracted genomic DNA (500 ng) was amplified from each patient’s pair-matched PBMC and tumor biopsy samples in a bias-controlled multiplex PCR followed by high-throughput sequencing. Sequences were collapsed and filtered to identify and quantify the absolute abundance of each unique TCRβ CDR3 region for further analysis, as previously described^48–50^.

### TCRseq analysis

Raw sequence reads were demultiplexed according to Adaptive’s proprietary barcode sequences. Demultiplexed reads were then further processed to remove adapter and primer sequences and to identify and remove primer dimer, germline and other contaminant sequences. The filtered data were clustered using both the relative frequency ratio between similar clones and a modified nearest-neighbor algorithm to merge closely related sequences to correct for technical errors introduced through PCR and sequencing. The resulting sequences were sufficient to allow the annotation of the V, D and J genes and the N1 and N2 regions constituting each unique CDR3 and the translation of the encoded CDR3 amino acid sequence. Gene definitions were based on annotation in accordance with the IMGT database (www.imgt.org). Data were analyzed using the immunoSEQ analyzer toolset.

### Single-cell RNAseq/TCRseq and digital gene expression

T cells were isolated and enriched from the week 12 posttreatment PBMC samples of patients 6, 7 and 8 using a human pan T cell isolation kit (cat. no. 130-096-535, Miltenyi Biotec), following the manufacturer’s instructions. For patient 8, two samples from the same time point were sequenced. Next-generation sequencing libraries were prepared using the 10x Genomics Chromium Single-Cell 5′ Reagent kit v2 per the manufacturer’s instructions. Libraries were uniquely indexed using the Chromium Dual Index kit, pooled and sequenced on an Illumina NovaSeq 6000 sequencer in a paired-end, dual-indexing run. Sequencing for each library targeted 20,000 mean reads per cell.

### Single-cell data preprocessing, quality control and analysis

Cell Ranger version 6.0.0 was used to demultiplex FASTQ reads, perform sequence alignment to the GRCh38 (Genome Reference Consortium Human Build 38) transcriptome and extract unique molecular identifier barcodes. Single-cell gene expression matrices were analyzed using the R package Seurat version 4.1.3 (ref. ^51^). Quality control was performed by excluding genes found in fewer than three cells, cells with <200 or >4,000 expressed genes, and cells with ≥25% mitochondrial RNA content. To avoid clonotype bias, we removed the TCRα and TCRβ genes from the count data^52^. The Seurat function SCTransform was used to normalize raw count data to a gamma–Poisson generalized linear model, perform variance stabilization, identify highly variable features, and scale features^53,54^. Integration of individual samples was performed using the Seurat functions FindIntegrationAnchors and IntegrateData. Cells were projected into the first 30 principal components using the RunPCA function in Seurat and further reduced into a two-dimensional visualization space using the RunUMAP function. Initial cell-type assignment was performed using the Seurat function MapQuery to perform reference mapping to an annotated human PBMC dataset. Cluster identities were then manually assigned by identification of differentially expressed genes using the MAST hurdle model, as implemented in the Seurat FindAllMarkers function with a log(fold-change) threshold of 0.25 and minimum fractional expression threshold of 0.25 (ref. ^55^). Canonical marker genes used for cluster identity included the following: CD4^+^ naive (*CCR7*), CD4^+^ T central memory (*IL7R*, *LTB*), CD4^+^ T_EM_ (*GZMK*, *CCL5*), CD4^+^ CTL (*GZMH*, *NGK7*, *GNLY*), CD4^+^ proliferating (*MKI67*, *TOP2A*), CD8^+^ naive (*CD8B*, *CCR7*), CD8^+^ T central memory (*CD8A*, *IL7R*), CD8^+^ T_EM_ (*CCL5*, *GZMK*, *GZMH*), CD8^+^ proliferating (*MKI67*, *CD8B*), double-negative (*NUCB2*, *FXYD2*), γδ T (*TRGV9*, *TRDV2*), mucosal-associated invariant T (*KLRB1*, *RORA*), NK (*NKG7*, *TYROBP*) and T regulatory (*FOXP3*, *RTNK2*) cells. For single-cell VDJ sequencing, only cells with full-length sequences were retained. Integration of the single-cell TCRseq data into the single-cell RNAseq data was performed using the R package scRepertoire^56^. Integration of Adaptive bulk TCRβ sequencing data and single-cell data was performed by comparing matching TCRβ CDR3 amino acid sequences between datasets.

### TCR-engineered constructs

High-frequency T cell clones were identified in patient-derived PBMC samples after vaccination by TCRseq and single-cell RNAseq analyses. Patient-specific clonal TCR sequences were gene optimized and inserted into the pMXs-IRES-GFP retroviral plasmid vector containing the viral packaging signal, transcriptional and processing elements, and the *GFP* reporter gene (GenScript). TCRβ and TCRα were positioned in sequence separated by a P2A (2A peptide derived from porcine teschovirus-1) cleavage site (TCRβ-P2A-TCRα). Retroviral particles encoding TCR constructs were generated by transfecting Phoenix-AMPHO cells (American Type Culture Collection) using Lipofectamine 3000 (Thermo Fisher Scientific) following the manufacturer’s instructions. Unvaccinated (pretreatment) patient-derived PBMCs (1 × 10^6^ cells) were retrovirally transduced to express the selected TCRs, as previously described^57^. Cells were cultured in RPMI medium supplemented with 10% FBS, 50 U ml^−1^ IL-2 and 1 ng ml^−1^ IL-7 (Peprotech) in a 5% CO_2_ humidified incubator for 10 days. The cell culture medium was refreshed every 2–3 days. TCR-engineered T cells were stimulated in vitro for 6 h with neoantigen pools (10 µg ml^−1^). Then, cells were evaluated by flow cytometry for T cell activation.

### ctDNA extraction, sequencing and analysis

Whole blood samples were collected when feasible in cell-free DNA (cfDNA) BCT Streck tubes (cat. no. 218997, Streck) at baseline (pretreatment), on week 3, and then Q3w until week 9 and Q9w thereafter. Plasma was separated from the cellular component and clarified using a double-spin protocol (1,600*g* for 10 min, 3,200*g* for 10 min). cfDNA was purified from plasma samples using the QIAamp circulating nucleic acid kit (cat. no. 55114, Qiagen). cfDNA was quantified using the cfDNA ScreenTape assay (Agilent). cfDNA samples from 13 patients who had baseline samples were batched and analyzed using personalized ctDNA assays. Up to 50 ng cfDNA was used as input for library preparation before enrichment and deep sequencing. Somatic mutation calls were made using Personalis ACE exome data from tumor tissue biopsy samples. Capture probe panels were designed for personalized targets. Advanced noise suppression, mutation calling, aggregate tumor tracking and measurable residual disease calling were performed using Personalis ctDNA technology.

### Statistical analysis

This study aimed to enroll 36 patients with advanced HCC. This article details results, as of the data cutoff date, from the full cohort of 36 patients enrolled. With a null hypothesis of an ORR of 16.9%, a sample size of 36 patients provides 80% power to detect an alternative hypothesis of at least 33.1% using a one-sided *α* of 0.10. No data were excluded from the analyses, and there was no randomization as part of the trial design. The key secondary analysis was performed with a one-sided exact binomial test, and results are reported with a one-sided 90% CI. Otherwise, descriptive statistics of counts and rates are used for categorical outcomes, and median times to events are used for time-to-event outcomes.

### Reporting summary

Further information on research design is available in the Nature Portfolio Reporting Summary linked to this article.

## Online content

Any methods, additional references, Nature Portfolio reporting summaries, source data, extended data, supplementary information, acknowledgements, peer review information; details of author contributions and competing interests; and statements of data and code availability are available at 10.1038/s41591-024-02894-y.

### Supplementary information

Supplementary Information Supplementary Figs. 1–8 and Table 1.

Reporting Summary

## Data Availability

Single-cell RNA sequencing data are deposited in the Gene Expression Omnibus under accession number GSE255830. TCRβ sequencing data can be found in the open-access immuneACCESS database under Digital Object Identifier 10.21417/RP2024NM. The TCR constructs used to evaluate T cell vaccine specificity are deposited in GenBank under accession numbers PP316119 (patient 8_TCR1), PP316120 (patient 8_TCR2), PP316121 (patient 8_TCR3), PP316116 (patient 5_c3-1), PP316117 (patient 5_c3-2) and PP316118 (patient 5_c6). Deidentified individual participant clinical data that underlie the results reported in this article are available for transfer. Interested investigators can obtain and certify the data transfer agreement and submit requests to the corresponding author (N.Y.S.). Investigators and institutions who consent to the terms of the data transfer agreement form, including but not limited to the use of these data for a specific project and only for research purposes, and to protect the confidentiality of the data and limit the possibility of identification of participants in any way whatsoever for the duration of the agreement will be granted access. Geneos will then facilitate the transfer of the requested deidentified data. This process is expected to be through a Geneos Secure File Transfer Service, but Geneos reserves the right to change the specific transfer method at any time, provided appropriate levels of access authorization and control can be maintained.

## Code availability

All custom code used to generate the results in this study has been deposited in a GitHub repository at https://github.com/FertigLab/GeneosCollaboration.
