# Supplementary material for: Personalized neoantigen vaccine and pembrolizumab in advanced hepatocellular carcinoma: a phase 1/2 trial
Source: Nat Med. 2024 Apr 7;30(4):1044–53. doi: 10.1038/s41591-024-02894-y (PMC11031401; doi:10.1038/s41591-024-02894-y)
Supplement: Supplementary file 1 — Supplementary Figs. 1–8 and Table 1. [file 41591_2024_2894_MOESM1_ESM.pdf]

nature medicine

Article https://doi.org/10.1038/s41591-024-02894-y

Personalized neoantigen vaccine and pembrolizumab in advanced hepatocellular carcinoma: a phase 1/2 trial

In the format provided by the authors and unedited

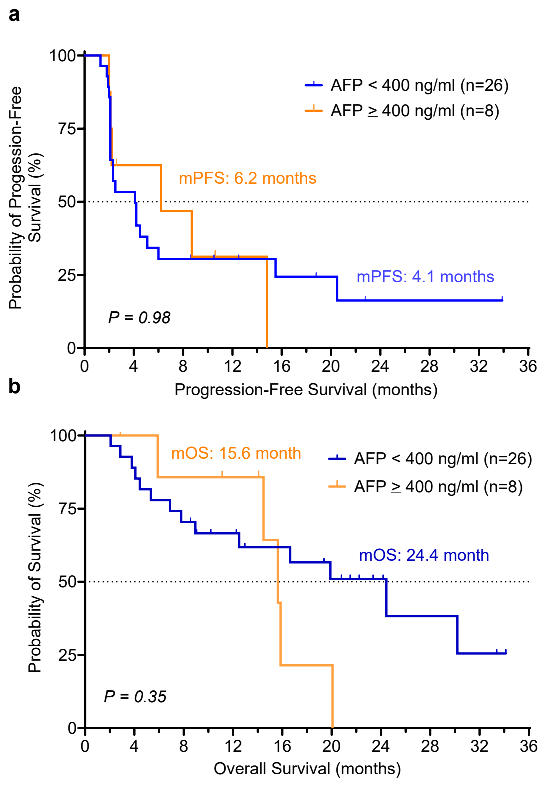

Supplemental Data Fig. 1 | Survival rates by AFP levels. a, Progression-free survival, **b**, and overall survival in patients with AFP>400 ng/ml or <400 ng/ml. Significance was evaluated by the Log-rank (Mantel-Cox) test.

a **b**

Differential Abundance Analysis of TCR Clones from Patient Samples

a — Data from PBMC samples (Pre- vs Post-treatment)

A	B	C	D	E	F
		Frequency of individual clones (pre-treatment)			
**Patient #**	**Total expanded clones in PBMC**	**Min**	**Max**	**Median**	**Ave**
8	42	0.00%	0.82%	0.00%	0.03%
14	55	0.00%	1.74%	0.01%	0.11%
3	84	0.00%	1.01%	0.00%	0.03%
4	29	0.00%	0.77%	0.00%	0.04%
5	33	0.00%	0.02%	0.00%	0.00%
6	27	0.00%	0.00%	0.00%	0.00%
7	24	0.00%	0.23%	0.00%	0.03%
11	25	0.00%	1.20%	0.00%	0.06%
12	58	0.00%	0.15%	0.00%	0.01%
15	48	0.00%	0.01%	0.00%	0.00%
17	132	0.00%	1.91%	0.00%	0.03%
19	87	0.00%	1.67%	0.00%	0.06%
20	90	0.00%	5.14%	0.02%	0.20%
22	47	0.00%	3.41%	0.00%	0.36%

A	G	H	I	J
	Frequency of individual clones (post-treatment)			
**Patient #**	**Min**	**Max**	**Median**	**Ave**
8	0.01%	2.11%	0.02%	0.16%
14	0.01%	3.62%	0.04%	0.24%
3	0.01%	1.51%	0.01%	0.07%
4	0.01%	0.92%	0.02%	0.06%
5	0.01%	0.04%	0.01%	0.01%
6	0.01%	0.11%	0.02%	0.03%
7	0.01%	0.57%	0.02%	0.06%
11	0.01%	1.58%	0.01%	0.09%
12	0.00%	0.23%	0.01%	0.02%
15	0.00%	0.03%	0.01%	0.01%
17	0.01%	2.60%	0.01%	0.07%
19	0.00%	2.25%	0.02%	0.10%
20	0.01%	6.63%	0.05%	0.29%
22	0.01%	5.37%	0.02%	0.48%

A	K	L	M
**Patient #**	**Overall abundance (pre-treatment)**	**Overall abundance (post-treatment)**	**Delta (post-pre %)**
8	1.42%	6.68%	5.27%
14	5.81%	13.25%	7.44%
3	2.89%	5.89%	3.01%
4	1.04%	1.72%	0.68%
5	0.06%	0.42%	0.35%
6	0.01%	0.76%	0.75%
7	0.66%	1.53%	0.87%
11	1.49%	2.19%	0.70%
12	0.37%	0.96%	0.60%
15	0.03%	0.44%	0.41%
17	4.36%	8.97%	4.62%
19	5.19%	8.54%	3.35%
20	17.81%	26.50%	8.70%
22	17.02%	22.54%	5.52%

			Percentage (pre-treatment)		Percentage (post-treatment)
Full cohort	Median	0.00%	0.00%	0.02%	0.02%
	Min	0.00%	0.00%	0.01%	0.01%
	Max	0.02%	0.02%	0.05%	0.05%

Median (Delta)	1.94%
Min (Delta)	0.35%
Max (Delta)	8.70%

Total expanded clones in PBMC		
	Median	47.5
	Min	24
	Max	132

b — Data from tumor samples

Expanded Clones Post-Treatment

N	O
**Number of Clones**	**Cum. Frequency of Clones**
27	1.57%
38	0.06%
36	0.44%
22	0.15%
15	0.13%
12	0.63%
19	0.07%
8	0.14%
14	0.18%
28	0.26%
21	1.54%
56	0.18%
63	0.31%
36	0.13%

Median	25	0.18%
Min	8	0.06%
Max	63	1.57%

Supplemental Data Fig. 2 | Overall T cell clone frequencies in the blood post-vaccination. a, Cumulative frequencies from significantly expanded T cell clones (min, max, median, average) per patient in the periphery. The average at pre- or post-treatment time points was multiplied by the total expanded clones per patient to identify the overall abundance contribution. The overall change was calculated by subtracting the pre-treatment from the post-treatment overall abundance (Delta post-pre). Individual frequencies, median, min, and max values of Delta are expressed in percentage. **b,** Expanded T cell clone numbers and frequencies in PBMC that are found in the tumor by differential abundance analysis. The median, min, and max values are reported for 14 evaluated patients.

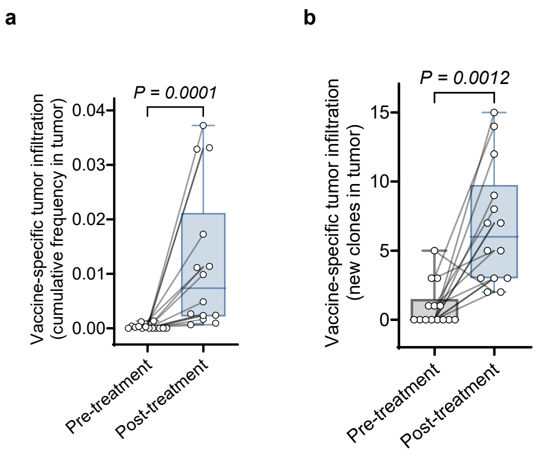

Supplemental Data Fig. 3 | Vaccine-specific new clone influx in the tumor. a, Cumulative frequencies of novel TCR rearrangements tracked from significantly expanded clones in the periphery in pre- and post-treatment tumor biopsies. **b,** Significantly expanded new T cell clone numbers found in pre- and post-treatment tumor biopsies. Pre-existing, significantly expanded clones were identified by the differential abundance statistical framework and excluded from the analyses **(a**,**b)**. Open circles represent individual patients (n=14 per group), the box extends from the 25th to the 75th percentile, the line inside the box is the median, and the whiskers extend from the minimum to maximum values. Significance was evaluated by a two-tailed, Wilcoxon rank test.

a

ID	# cells expanded post PTCV	Percent (%)
CD4 TCM	11	1.06
CD4 TEM	1	0.10
CD4 CTL	6	0.58
CD4 Proliferating	4	0.38
CD8 TCM	4	0.38
CD8 TEM	909	87.32
CD8 Proliferating	85	8.17
dnT	8	0.77
gdT	4	0.38
NK	3	0.29
Treg	6	0.58
**Total**	1041	100

b

ID	# cells expanded post PTCV	Percent (%)
CD8 TEM_1	46	5.06
CD8 TEM_2	84	9.24
CD8 TEM_3	337	37.07
CD8 TEM_4	3	0.33
CD8 TEM_5	429	47.19
CD8 TEM_6	10	1.10
CD8 TEM_7	0	0
**Total**	909	100

Supplemental Data Fig. 4 | Genetic signature by cluster of significantly expanded TCR post-PTCV. Pair-matched TCRβ and single-cell sequencing of PBMC at week 12 post-PTCV from Pt #6, #7, and #8. The CDR3 identity of significantly expanded TCRs was determined by differential abundance framework analysis. Number of cells and percentage found in **a,** vaccine expanded TCRβ (n=1041), and **b,** CD8 TEM subclusters.

a

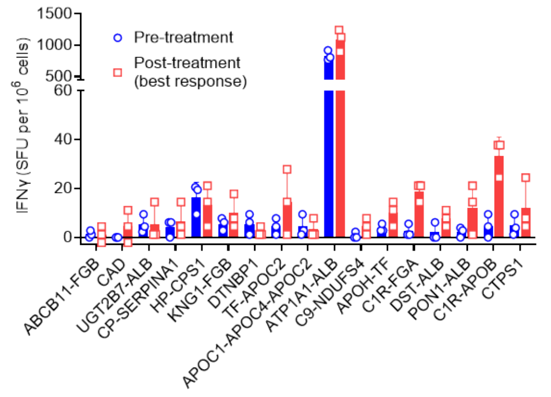

b

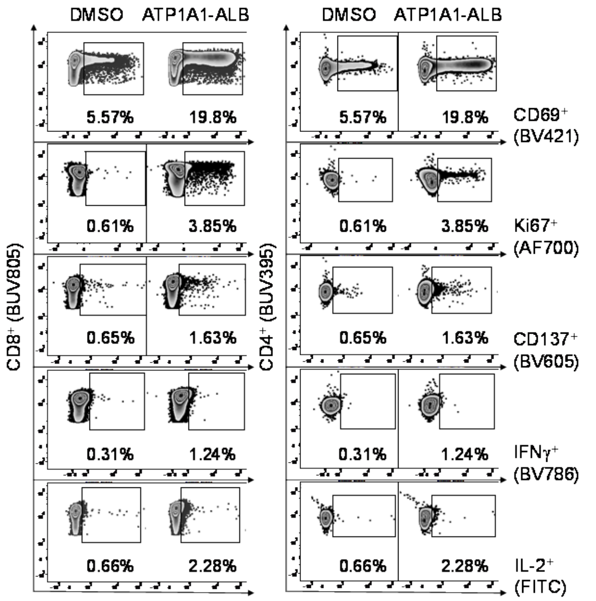

c

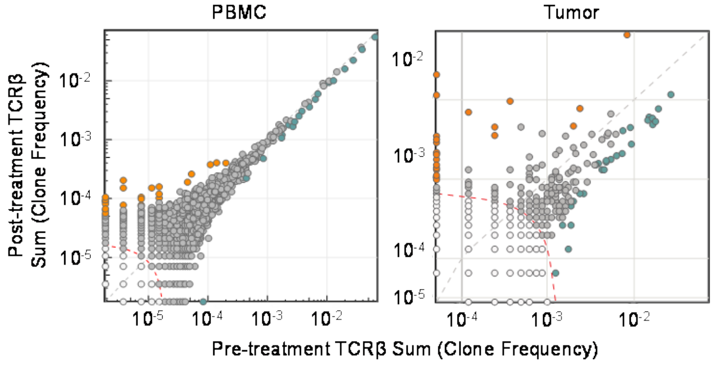

d

Construct ID	CDR3 sequence	Tumor freq. pre. Vax (%)	Tumor freq. post. Vax (%)
Pt #5_c3-1<br>Pt #5_c3-2	CSARDLYRNTEAFF	0.54	1.59
Pt #5_c6	CASSRQGQEQYF	1.62	3.73

e

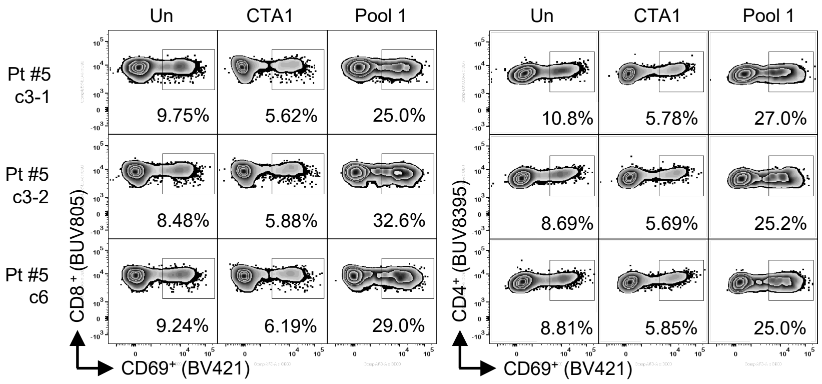

Supplemental Data Fig. 5 | *Ex vivo* validation (in a second patient) of specificity of GNOS-PV02 driven new TCR clones post-vaccination and their reactivity to a specific vaccine-encoded antigen. a, PBMCs from patient #5 (3x10^5/per well) were stimulated with vaccine-encoded epitopes at the concentration of 10 µg/mL for 18–24 hours. Cells were evaluated for the presence of vaccine-induced neoantigen-specific responses before and post-personalized GNOS-PV02 vaccination using an interferon IFNγ ELISPOT assay without cytokine stimulation. The bar indicates the mean SFU of n=3 individual technical replicates ± SD. **b,** T cells from the patient were isolated and expanded from PBMCs stimulated *in vitro* with the immunodominant epitope ATP1A1-ALB (10 µg/mL) and a cocktail of cytokines (IL-2/IL-4/IL-7). Four days later, the expression of T cell activation markers such as CD69, Ki67, CD137, IFNγ, and IL2 were assessed by flow cytometry. **c,** Most frequent TCRs identified by TCRβ and RNA sequencing in patient (Pt) #5. Pre-vaccination versus week 9 post-vaccination (Pair-wise Scatter Plot). Orange, green, and grey circles represent expanded, contracted, and not significantly changed T cell clones, respectively, in the matched pre- and post-treatment PBMC and tumor samples (based on differential abundance statistical analysis). **d,** Evaluated TCRs were selected for cloning based on their substantial increase in frequency (pre- vs post-vaccination) and occurrence in high frequency in the tumor post-vaccination. The patient-specific clonal TCR sequences were gene optimized using GOAL algorithm and inserted into the pMXs-IRES-GFP retroviral plasmid vector containing viral packaging signal, transcriptional and processing elements, and GFP reporter gene. Two constructs were designed for Pt #5_c3 as it presented two different TRA sequences and a shared TRB sequence. **e,** TCR-engineered T cells (GFP positive) confirm antigen-specific reactivity to Pt #5's GNOS-PV02 encoded antigens ((6 hours-stimulation with Pool 1 or the unspecific epitope CTA1, 10 ug/mL)) detected by the expression of CD69 as evaluated by flow cytometry.

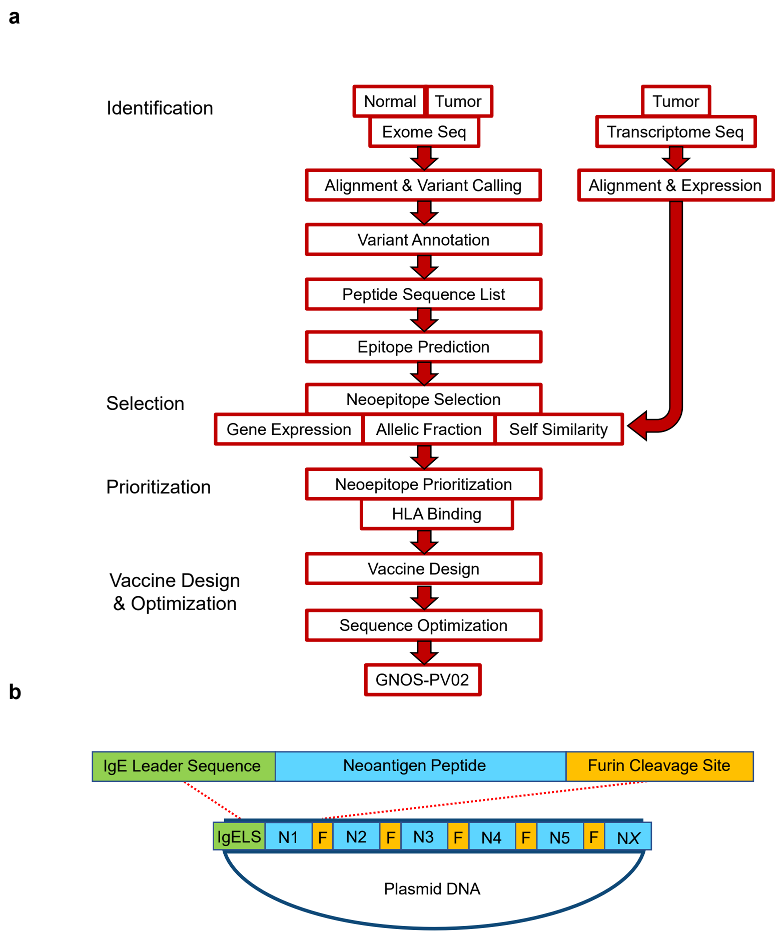

Supplemental Data Fig. 6 | PTCV design and construction. a, Flow chart describing neoantigen identification, selection, prioritization, vaccine design and sequence optimization. Tumor and normal samples were taken for DNA and RNA isolation and sequencing. Identified neoantigens were prioritized according to expression and MHC binding. The final vaccine insert was gene optimized and cloned into a DNA plasmid vector. **b**, Plasmid DNA design. The optimized plasmid insert encodes an IgE leader sequence at its N-terminus, followed by up to 40 neoepitope peptides separated by furin cleavage sites. The neoantigen peptides consist of the predicted MHC class I epitopes where the core amino acid mutation is flanked by 16 residues on either side.

	Descriptive statistics (ELISPOT)	
	Viability %	Cell count
Events	124	124
Average	91.9	3.71E+07
Median	92.9	3.39E+07
Min	72.1	6.00E+06
Max	98.8	1.30E+08

Supplemental Data Fig. 7 | Descriptive statistics (ELISpot). a, Cell viability percentage and counts upon PBMC vial thaw from 124 ELISpot assays across the cohort.

a

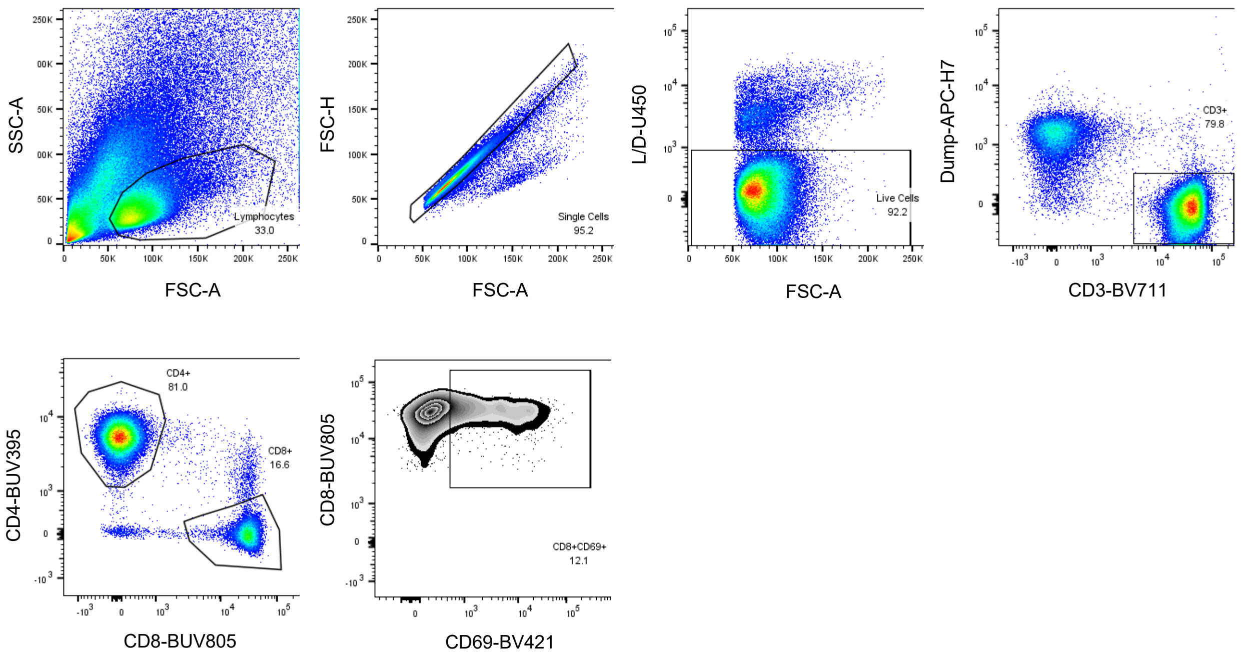

b

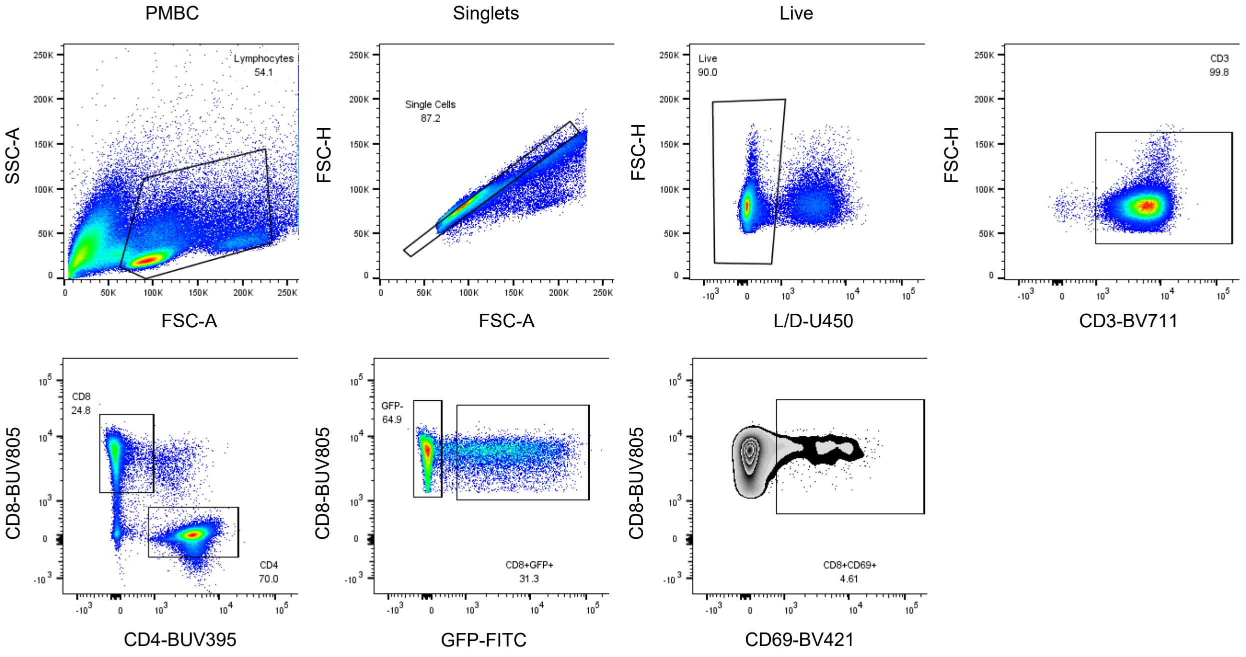

Supplementary Data Fig. 8 | Gating strategy (left to right). a, IVS and ICS: Cells were gated on PBMCs (forward scatter (FSC)-A versus side scatter (SSC)-A), singlets (FSC-A versus FSC-H), live cells (FSC-H versus Live/Dead), lymphocytes (CD3+ versus dump markers), CD8 or CD4 T cells (CD8 versus CD4) and cytokine+ cells. Representative density plots (patient 22) of individual T cell activity markers CD69+, Ki67+, CD107A+, IFNγ+, and TNFα+ upon stimulation with patient-specific PTCV epitope pools. **b,** TCR-engineered constructs: Cells were gated on PBMCs (forward scatter (FSC)-A versus side scatter (SSC)-A), singlets (FSC-A versus FSC-H), live cells (FSC-H versus Live/Dead), lymphocytes (CD3), CD8 or CD4 T cells (CD8 versus CD4), GFP positive (CD8 or CD4 versus GFP) and CD69+ cells.

Patient #	Race	Sex	Age	Etiology	AFP ≥ 400 ng/ml at baseline (Y or N)	# of Targetable neos	# of vaccination	Best response
1	asian	male	55	HBV	Y	5	3	PD
2	white	male	64	HCV	N	9	5	SD
3	asian	male	70	Non-viral	N	31	4	PD
4	asian	male	65	HCV	Y	11	12	SD
5	asian	male	61	HBV	N	17	4	PD
6	Maori	male	60	Non-viral	N	23	12	SD
7	white	female	61	HCV	N	40	18	CR
8	white	male	63	HCV	Y	48	5	PR
9	white	male	70	HCV	N	20	13	SD
10	white	male	76	HCV	Y	4	8	PD
11	white	male	75	Non-viral	N	29	9	PR
12	white	male	75	HCV	N	42	4	SD
13	white	male	73	Non-viral	N	43	3	PD
14	white	male	78	Non-viral	N	11	3	PD
15	Pacific	female	63	Non-viral	N	27	8	PD
16	black	female	77	Non-viral	N	55	3	PD
17	white	male	73	HCV	N	49	10	CR
18	white	female	68	Non-viral	N	52	5	PR
19	white	female	48	Non-viral	N	14	14	PR
20	asian	male	64	HBV	N	34	5	PD
21	asian	male	40	HBV	N	34	10	PD
22	white	male	72	HCV/HBV	N	47	11	CR
23	white	female	72	HBV	N	26	3	unevaluable
24	asian	male	64	HCV	N	43	1	unevaluable
25	asian	female	51	HBV	Y	25	9	SD
26	white	female	83	Non-viral	N	38	9	PR
27	white	male	64	HCV	N	19	8	PR
28	Pacific	male	63	HCV	N	67	4	PD
29	Pacific	female	60	HBV	Y	36	8	SD
30	white	female	79	Non-viral	Y	27	5	PD
31	white	male	77	Non-viral	N	48	7	PR
32	Pacifc	male	58	HBV	N	11	6	SD
33	white	male	58	Non-viral	N	25	4	SD
34	white	male	68	HCV	N	34	5	PD
35	Maori	female	73	Non-viral	Y	22	5	PR
36	white	male	71	Non-viral	N	37	5	PD

Supplemental Data Table 1. Individualized patient demographic information

Protocol

This trial protocol has been provided by the authors to give readers additional information about their work.

Protocol for: **Yarchoan M, et al. Personalized Neoantigen Vaccine and Pembrolizumab in Advanced Hepatocellular Carcinoma: A phase 1/2 trial.**

This supplement contains the following:

1. Current protocol, protocol synopsis

STUDY PROTOCOL

An Open-label, Multi-center, Phase I/IIa Study of a Personalized Neoantigen DNA Vaccine (GNOS-PV02) and Plasmid encoded IL-12 (INO-9012) in Combination with Pembrolizumab (MK-3475) in Subjects with Advanced Hepatocellular Carcinoma

Protocol Amendment 7
Protocol Version Date: 17Oct2023

CONFIDENTIAL

The information in this document is considered privileged and confidential by Geneos Therapeutics, Inc. and may not be disclosed to others except to the extent necessary to obtain Institutional Review Board (IRB)/Research Ethics Board (REB)/Ethics Committee (EC) approval and Informed Consent, or as required by local regulatory authorities. Persons to whom this information is disclosed must be informed that this information is privileged and confidential and that it should not be further disclosed without the written permission of Geneos Therapeutics. Any supplemental information added to this document is also confidential and proprietary information of Geneos Therapeutics and must be kept in confidence in the same manner as the contents of this document.

An Open-label, Multi-center, Phase I/IIa Study of a Personalized Neoantigen DNA Vaccine (GNOS-PV02) and Plasmid encoded IL-12 (INO-9012) in Combination with Pembrolizumab (MK-3475) in Subjects with Advanced Hepatocellular Carcinoma

**Study Sponsor:**	Geneos Therapeutics, Inc.<br>3675 Market Street<br>Suite 200<br>Philadelphia, PA 19104
**Investigational product:**	GNOS-PV02
**IND number:**	[redacted]
**Protocol number:**	GT-30
**Phase:**	I/IIa
**Final protocol:**	06Nov2019
**Amendment 1:**	24Jun2020
**Amendment 2:**	12Nov2020
**Amendment 3:**	12Mar 2021
**Amendment 4:**	13Dec2021
**Amendment 5:**	11Aug2022
**Amendment 6:**	03Feb2023
**Amendment 7:**	17Oct2023
**Sponsor emergency contact:**	[redacted]
**Cell:**	[redacted]

Regulatory Statement

This study will be performed in compliance with the protocol and in accordance with Good Clinical Practice (International Conference on Harmonization, Guidance E6 (R2), March 2018), principles of human subject protection, and applicable country-specific regulatory requirements.

Confidentiality Statement

The information in this document contains trade secrets and commercial information that are privileged or confidential and may not be disclosed unless such disclosure is required by applicable laws and regulations. In any event, persons for whom this information is disclosed must be informed that the information is privileged or confidential and may not be further disclosed by them. These restrictions on disclosure will apply equally to all future information supplied to you that is indicated as privileged or confidential.

VERSION HISTORY PAGE

Version 1.0, 06Nov2019
Initial Creation

Version 2.0, 24Jun2020 (Protocol Amendment 1)

Changes from Version 1.0

- Exclusion criteria added: Less than 2 sites available for injection and presence of tattoos/keloids/hypertrophic scars within 2 cm of the injection site.
- Added language to Section 8.1.2.1 to specify the appropriate sites for injection and electroporation.
- *"Subjects will receive one ID injection of GNOS-PV02 + INO-9012 above each of two acceptable muscle locations for injection, each in a volume of ~0.1 mL. The injection site is assessed for IP leakage and followed immediately by EP using the CELLECTRA® 2000 device. Only if the deltoid muscle is not a suitable location (see exclusion criterion '23'), the ID injection can then be administered above the lateral quadriceps followed immediately by EP. The IP must not be given within 2 cm of a tattoo, scar, keloid, previous injection site or active lesion/rash. Also, ID injection followed immediately by EP must not be performed in the muscle of an extremity which has any metal implants or implantable medical device (e.g., surgical rods, pins or joint replacements). If a subject has an implanted cardiac defibrillator or pacemaker, ID injection/EP must not be performed above the deltoid muscle on the same side of the body where the device is located."*
- Section 8.3: Changes were made to the subject study number. *"All subjects who sign the ICF will be assigned an 8-digit subject study number which will be retained for the duration of the study. GT-30-2 +1-digit unique site no. + 2-digit subject no. (GT-30-2101)."*
- Removed HPV/EBV assessment at screening, if unknown.
- Clarified that a negative urine test is sufficient to prove that the patient is not pregnant. Serum test only required if urine test is indeterminate.
- Modified Figure 1 Study schema to clarify that the initial screening visit doesn't require a RECIST assessment.
- Clarification and consistency throughout the protocol amendment that AEs will be collected from Day 0 until the end of the study.
- Modification explaining that intradermal administration is '*less painful*' than intramuscular administration (replacing '*more tolerable*').
- Device return instructions have been completed with telephone number and email address.
- Non-acceptance of HCC subtypes (fibrolamellar, sarcomatoid, mixed cholangiocarcinoma) has been specified in inclusion criteria.
- Language has been modified to include notification to 'regulatory authorities', not only FDA.
- Section 10.2.10.2: Additional language added to specify the preferred method to collect the 3 biopsy cores. 'preferably two (2) fresh-frozen with RNAlater and one (1) formalin fixed and paraffin embedded (FFPE)'.
- Included ctDNA in schedule of assessments for clarity on timing of assessment.
- Included Free T3 as alternative to T3 in assessment of thyroid function.
- Extend visit window from 2 to 3 days to accommodate patient schedules.
- Grammatical, editorial, and administrative updates have been made throughout the protocol amendment for consistency.

Version 3.0, 12NOV2020 (Protocol Amendment 2)
**Changes from Version 2.0**<br>• Expand the number of subjects from approximately 12 to approximately 24, and update of statistical considerations of the sample size increase.<br>• Not require contraindication/intolerance to CT scan to allow MRI. Similar to contrast-enhanced HCC, MRI is a highly sensitive technique for detection and characterization of HCC lesions.<br>• Remove the requirement in the protocol to report administration site reactions as an AESI. Based on historical use and reported adverse events with the CELLECTRA 2000® device for Intradermal administration, Geneos Safety has concluded that there is no need to continue collecting injection site reactions as AESIs. Injection site reactions will continue to be collected as AE in the eCRF and those that meet criteria for seriousness will be collected and reported as SAE according to the protocol.<br>• Clarification that investigational product (IP) can be administered on previous injection sites if they present with no active lesions at the time of administration. Amendment 1 specified that IP should not be administered over lesioned skin, however, the writing seemed to suggest that it should also not be administered on healthy skin where it had been previously administered at an earlier visit. In amendment 2 we clarify that language.<br>• Modification of abbreviated inclusion criteria as follows:<br>o Platelets: ≥75,000/µL (instead of ≥100,000/ µL)<br>o Bilirubin: ≤2.5×ULN (instead of ≤2 mg/dL)<br>o Albumin: ≥2.8 g/dL (instead of ≥3 g/dL)<br>Rationale: Although there is no modification for inclusion criteria at screening, abbreviated inclusion criteria, which occur immediately before the first dose of investigational product, have been modified to permit dosing of patients whose platelets, bilirubin or albumin levels have slightly worsened from screening. COVID-19 has resulted in delayed manufacturing timelines of the personalized vaccines. This longer manufacturing timelines put at risk the ability of the patients to receive their personalized treatment if the same analytical levels are required before dosing compared to screening.
**Version 4.0, 12Mar2021 (Protocol Amendment 3)**
**Changes from Version 3.0**<br>• Allow any non-live COVID-19.<br>• Reword exclusion criteria #12 to change specific tumor types allowed (curatively treated basal cell carcinoma of the skin, squamous cell carcinoma of the skin, and/or curatively resected in situ cervical and/or breast cancers. Subjects with history of early-stage prostate cancer that has been curatively treated or appropriate for observation may be enrolled) to a general rule ((a) non-invasive carcinomas subject to successful curative treatment in the opinion of the investigator which require no further therapy and (b) other malignancies for which patients have undergone potentially curative therapy and have been considered disease free for at least 3 years prior to screening).<br>• Specify in exclusion criteria #19 that oncolytic viruses are not considered immunotherapy.<br>• Clarify the steroid dose allowed in prohibited medications.<br>• Change biopsy sample preparation preferences to remove the preference for fresh-frozen instead of FFPE.
**Version 5.0, 13Dec2021 (Protocol Amendment 4)**
• Expand the number of subjects from approximately 24 to approximately 36, and update study duration and statistical considerations of the sample size increase.

Version 6.0, 11Aug2022 (Protocol Amendment 5)
• Allow continued treatment with GNOS-PV02+INO-9012 for 36 months.<br>• After 2-years of treatment, GNOS-PV02+INO9012 will be administered Q12W.
**Version 7.0, 03Feb2023 (Protocol Amendment 6)**
• Provide additional clarity to version 6 of the protocol.<br>• Removed duplicate figure 1.1 Study Schema<br>• Added language confirming subjects with CR, PR or SD who are still receiving treatment at the end of approx. 2 years (week 99) are eligible to continue with GNOS-PV02 treatment every 12 weeks until week 147.<br>• Table 3 Schedule of Assessments updated Tumor Measurements, Immunology and ctDNA to be collected every 12 weeks (post 24 mo).<br>• Confirmatory scans are requested in the 4-6 weeks' timeframe after the first radiographic evidence of PR/CR.<br>• Subjects who have disease progression with evidence of new lesions while receiving GNOS-PV02 treatment may have a new personalized vaccine manufactured and administered using a newly biopsied lesion, as per investigator's discretion and Sponsor's approval.<br>• Clarified Section 11.2: (i) Removed language indicating ORR determination at 12 weeks since we can only determine PD rate (but not SD/PR/CR) based on first on-treatment scan at week 9 i.e.we can only determine (1-ORR) by week 12; and (ii) statistical testing will be only done once and only on the full cohort of 36 patients.
Version 8.0, 17Oct2023 (Protocol Amendment 7)
• Subjects with CR, PR or SD who are still receiving treatment at the end of approximately 2 years (Week 99) are eligible to continue with GNOS-PV02 treatment every 12 weeks for approx. 5 years and every 26 weeks (q6mos) thereafter until confirmed disease progression, investigator discretion or no additional study drug available.

SPONSORS PROTOCOL AMENDMENT 7 SIGNATURE PAGE

**Sponsor:**	Geneos Therapeutics, Inc.
**Address:**	660 W. Germantown Pike, Suite LL 100, Plymouth Meeting, PA 19462
**Protocol Title**	An Open-label, Multi-center Phase I/IIa Study of a Personalized Neoantigen DNA Vaccine (GNOS-PV02) and Plasmid encoded IL-12 (INO-9012) in Combination with Pembrolizumab (MK-3475) in Subjects with Advanced Hepatocellular Carcinoma
**Protocol Number**	GT-30
**Protocol Amendment**	7
**Version:**	8.0
**Final Protocol Date:**	17Oct2023

I approved the protocol and confirmed that the protocol follows the current ICH/GCP guidelines.

31Oct2023

[redacted] Date

31 Oct 2023

[redacted] Date

TABLE OF CONTENTS

1. PROTOCOL AMENDMENT 7 SUMMARY/PROTOCOL AMENDMENT 7 SYNOPSIS 14

1.1. Study Schema 14

2. TERMS, ACRONYMS, ABBREVIATIONS 28

3. INTRODUCTION 31

3.1. Background 31

3.2. Rationale 31

3.2.1 Rationale for the Study and Selected Subject Population 31

3.2.2 Unmet Medical Need for Patients with Advanced HCC 31

3.2.3 Intradermal delivery of GNOS-PV02 + INO-9012 using CELLECTRA® 2000 device 40

3.3. Rationale for Dose Selection/Regimen 40

3.4. Benefit/Risk 40

4. PHARMACEUTICAL AND THERAPEUTIC BACKGROUND 40

4.1. Neoantigen DNA Vaccine, GNOS-PV02 40

4.1.1 Chemical Name and Structure 40

4.1.2 Manufacturing facility 42

4.1.3 Manufacturing process 43

[redacted] 43

[redacted] 43

[redacted] 43

[redacted] 44

[redacted] 44

[redacted] 44

[redacted] 45

[redacted] 46

5. OBJECTIVES AND ENDPOINTS 46

6. STUDY DESIGN 47

6.1. Research Hypotheses 47

6.2. Overall Design 47

6.3. End of Study Definition 49

7. STUDY POPULATION 50

7.1. Inclusion Criteria 50

7.2. Exclusion Criteria 51

7.3. Abbreviated Inclusion and Exclusion criteria (After Completion of Standard of Care TKI Therapy, Prior to Investigational Therapy) ... 54
7.3.1 Inclusion ... 54
7.3.2 Exclusion ... 55
7.4. Screen Failures ... 55
7.5. Strategies for Recruitment and Retention ... 56
8. STUDY INTERVENTION ... 56
8.1. Study Intervention(s) Administration ... 56
8.1.1 Study Treatment Description ... 56
8.1.2 Dosing, Administration and Preparation ... 56
8.2. Preparation/Handling/Storage/Accountability ... 57
8.2.1 Acquisition and Accountability ... 57
8.2.2 Formulation, Appearance, Packaging and Labeling for GNOS-PV02 + INO-9012 ... 58
8.2.3 Product Storage and Stability ... 58
8.3. Randomization and Blinding ... 58
8.4. Concomitant Therapy ... 59
8.4.1 Allowed concomitant treatments ... 59
8.4.2 Prohibited Medications or Treatments During Study ... 59
8.4.3 Rescue Medication ... 60
8.5. Dose Modification and/or Interruption ... 60
9. STUDY INTERVENTION DISCONTINUATION AND PARTICIPANT DISCONTINUATION/WITHDRAWAL ... 60
9.1. Discontinuation of Study Intervention ... 60
9.2. Participant Discontinuation/Withdrawal from the Study ... 60
9.3. Lost to Follow-up ... 61
10. STUDY ASSESSMENTS AND PROCEDURES ... 61
10.1. Efficacy Assessments ... 61
10.1.1 Tumor Assessment ... 61
10.2. Safety Assessments ... 64
10.2.1 Demographics and Medical History ... 64
10.2.2 Physical Examinations ... 65
10.2.3 Eastern Cooperative Oncology Performance Status ... 65
10.2.4 Vital Signs ... 65

10.2.5 Electrocardiograms 65
10.2.6 Clinical Safety Laboratory Assessments 65
10.2.7 Hepatitis and HIV Screening 66
10.2.8 Pregnancy Test 66
10.2.9 TSH, T3, and FT4 66
10.2.10 Immunogenicity Assessments 67
10.3. Adverse Events and Serious Adverse Events 70
10.3.1 Definition of Adverse Event 70
10.3.2 Definition of Serious Adverse Event 70
10.3.3 Classification of an Adverse Event 72
10.3.4 Adverse Event Reporting 73
10.3.5 Serious Adverse Event Reporting 73
10.3.6 Serious and unexpected suspected adverse reaction 74
10.3.7 Unanticipated (Serious) Adverse Device Effect 75
10.3.8 Dose-Limiting Toxicities 75
10.3.9 Stopping Rules 75
10.3.10 Adverse Events of Special Interest 76
10.3.11 There are no defined Adverse Events of Special Interest for this study. Reporting of Pregnancy 76
10.3.12 Time Period and Frequency for Event Assessment and Follow-Up 76
10.3.13 Safety Committee 76
11. STATISTICAL CONSIDERATIONS 76
11.1. Statistical Hypotheses 77
11.2. Sample Size Determination 77
11.3. Populations for Analyses 77
11.4. General Statistical Methods 77
11.5. Primary Safety Analyses 78
11.6. Primary Immunogenicity Analyses 78
11.7. Secondary Efficacy Analyses 78
11.7.1 Safety Stopping Rule 79
11.8. Exploratory Analyses 80
11.9. Analysis of Other Safety Data 80
11.10. Disposition 80

11.11. Demographic and Other Baseline Characteristics....... 80
11.12. Interim Analyses 81
11.13. Multiplicity 81
11.14. Missing Data 81
11.15. Randomization and Blinding 81
12. SUPPORTING DOCUMENTATION & OPERATIONAL CONSIDERATIONS 81
12.1. Regulatory, Ethical, and Study Oversight Considerations 81
12.1.1 Regulatory and Ethical Issues 81
12.1.2 Financial Disclosure....... 81
12.1.3 Informed Consent Process 82
12.1.4 Data Protection....... 82
12.1.5 Committee Structure 82
12.1.6 Data Quality Assurance....... 83
12.1.7 Source Documents 83
12.1.8 Study and Site Closure....... 84
12.1.9 Publication Policy 84
13. REFERENCE LIST 84

LIST OF TABLES

Table 1. Adequate Organ Function Laboratory Values 20

Table 2. Study Treatments 23

Table 3. Schedule of Assessments (SOA) 23

Table 4. Results of Immune Checkpoint Inhibitors and Combination Therapy 34

Table 5. Total Subjects and Doses Administered via CELLECTRA® 2000 Device IM, ID, and Overall (Number of Studies Included = 37) 45

Table 6. Adequate Organ Function Laboratory Values 51

Table 7. Adequate Organ Function Laboratory Values 54

Table 8. Imaging and Treatment After First Radiologic Evidence of Progressive Disease 63

Table 9. Test Analytes for Laboratory Assessments 66

Table 10. Guidelines for Adverse Event Severity 72

Table 11. Time Point Response: Patients with Target (± Non-target Disease) 94

Table 12. Time Point Response: Patients with Non-target Disease Only 95

Table 13. Best Overall Response when Confirmation of Complete Response and Partial Response required 95

Table 14. Assigning Time Point Response for iRECIST 98

Table 15. Assigning Best Overall Response for iRECIST 99

LIST OF FIGURES

Figure 1. Schema of the Study of a Personalized Neoantigen DNA Vaccine (GNOS-PV02) and Plasmid Encoded IL-12 (INO-9012) in Combination with Pembrolizumab (MK-3475) in Subjects with Advanced Hepatocellular Carcinoma 14

Figure 2. How Electroporation Works in the Body 38

Figure 3. Map of Representative Patient-specific Personalized Neoantigen Vaccine (GNOS-PV02) Plasmid (pGNOS-PV02-30-2YYY) and the Structure of the Polyepitope Insert 42

Figure 4. Map of IL-12 Plasmid pGX6001 42

Figure 5. Conditions for Continuing Study Treatment in the Presence of Increased Radiographic Tumor Size 64

1. PROTOCOL AMENDMENT 7 SUMMARY/PROTOCOL AMENDMENT 7 SYNOPSIS

1.1. Study Schema

Figure 1. Schema of the Study of a Personalized Neoantigen DNA Vaccine (GNOS-PV02) and Plasmid Encoded IL-12 (INO-9012) in Combination with Pembrolizumab (MK-3475) in Subjects with Advanced Hepatocellular Carcinoma

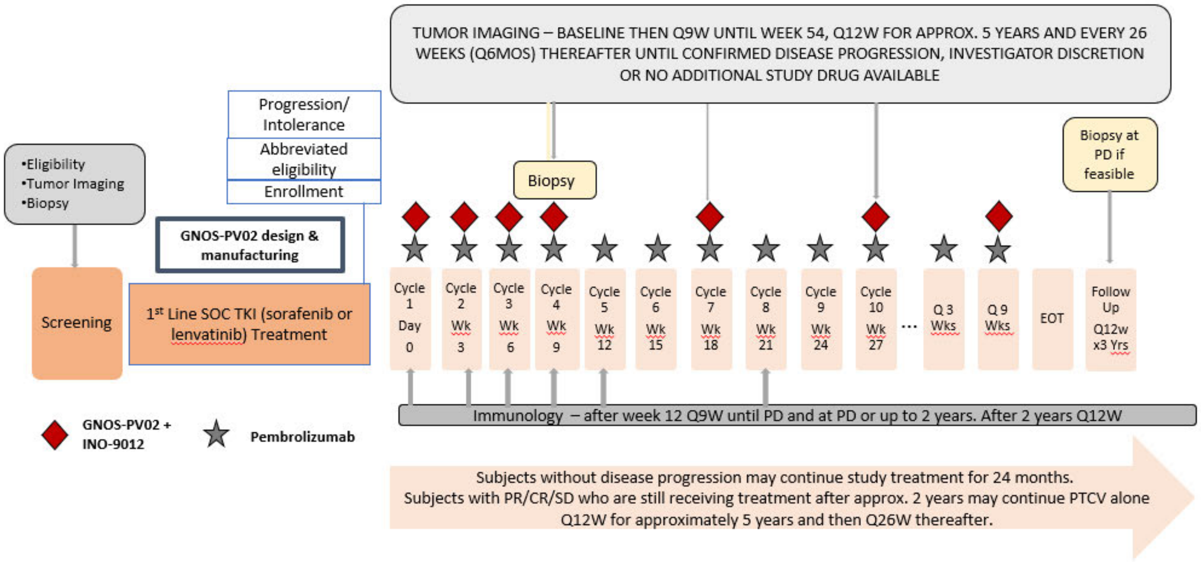

**Abbreviated Title**	A Phase I/IIa Study of a Personalized Neoantigen DNA Vaccine (GNOS-PV02) and Plasmid encoded IL-12 (INO-9012) in Combination with Pembrolizumab (MK-3475) in Subjects with Advanced Hepatocellular Carcinoma
**Sponsor Product Identifiers**	GNOS-PV02<br>Personalized Neoantigen DNA Vaccine<br>INO-9012<br>Plasmid encoded IL-12<br>MK-3475<br>Pembrolizumab
**Study Phase**	Phase I/IIa
**Clinical Indication**	Hepatocellular carcinoma (HCC)
**Study Type**	Interventional
**Type of control**	No treatment control
**Route of administration**	GNOS-PV02 + INO-9012 Intradermal (ID) followed by CELLECTRA® 2000 electroporation (EP)<br>Pembrolizumab Intravenous (IV)
**Study Blinding**	Unblinded, open-label
**Treatment Groups**	Pembrolizumab 200 mg IV every 3 weeks (Q3W) until week 102, concurrently with GNOS-PV02 (1 mg) + INO-9012 (0.34 mg) ID every 3 weeks for 4 doses, every 9 weeks (Q9W) until week 99, every 12 weeks (Q12W) for approximately 5 years, then every 26 weeks (W26W) until disease progression, investigator discretion or depletion of IP
**Number of study subjects**	Approximately 36 subjects will be treated
**Number of study sites**	Up to 4 sites
**Estimated duration of study**	The Sponsor estimates that the study will require approximately 54 months from the time the first subject signs the informed consent until the last subject's last study-related phone call or visit. The enrollment period is estimated to be about 35 months.

**Duration of Participation**	Each subject will participate in the study from the time the subject signs the informed consent form (ICF) through the final contact. Study participants will initially receive standard of care first-line treatment with lenvatinib or sorafenib. During first-line treatment, follow-up visits, blood work, and restaging scans will be scheduled at the discretion of the treating oncologist per standard of care. As this treatment is considered standard of care, no adverse events (AEs), will be collected. Discontinuation of first-line standard of care therapy will occur at the discretion of the treating oncologist. Upon discontinuation of first-line therapy and guidance from the treating physician, subjects will begin receiving pembrolizumab Q3W and the personalized neoantigen DNA vaccine every 3 weeks for 4 doses and then Q9W until week 99. Treatment will continue until progressive disease (PD), unacceptable adverse events intercurrent illness that prevents further administration of treatment, investigator's decision to withdraw the subject, subject withdrawal of consent, pregnancy of the subject, noncompliance with study treatment or procedure requirements, subject receiving approx. 2 years of pembrolizumab or GNOSPV02, or administrative reasons requiring cessation of treatment. Subject may continue to be treated with GNOS-PV02 and pembrolizumab post progression as long as deemed clinically stable by the physician. Subjects who stop pembrolizumab or GNOS-PV02 as a result of obtaining a confirmed complete response (CR) or those subjects who stop after receiving 2 years of study drug may be eligible to receive GNOS-PV02 Q12W, at the discretion of the investigator, for an additional 1 year after PD, if they meet the criteria for retreatment (Second Course Phase). Subjects with CR, PR or SD who continue to receive treatment at the end of approx. 2 years (week 99) are eligible to continue with GNOS-PV02 treatment every 12 weeks for approximately 5 years and every 26 weeks until disease progression, investigator discretion or depletion of IP. Subjects who discontinue study treatment for reasons other than PD will have post-treatment follow-up visits for monitoring disease status until PD, initiating a non-study cancer treatment, withdrawing consent from study participation, or becoming lost to follow-up. All subjects will be followed (e.g., by telephone or visit) for overall survival (OS) until death, withdrawal of consent from study participation, loss to follow-up, or the end of the study, whichever comes first. After the last dose of study drug, each subject will be followed for 30 days for AE monitoring. Serious adverse events (SAEs) will be collected until the patient reaches the end of the study.
**Study Design**	This is a single-arm, open-label, multi-site Phase I/IIa study of a personalized neoantigen DNA vaccine (GNOS-PV02) and plasmid encoded IL-12 (INO-9012) in combination with pembrolizumab (MK-3475) in subjects with histologically or cytologically confirmed diagnosis of HCC based on pathology report. To be eligible, subjects must be receiving or scheduled to begin first-line therapy with sorafenib or lenvatinib and must provide a tissue sample for personalized neoantigen DNA vaccine development that passes sequencing quality control. Upon discontinuation of first-line therapy, subjects will begin therapy with the personalized neoantigen DNA vaccine in combination with pembrolizumab. Additionally, subjects must also have disease not amenable to a curative treatment approach (e.g., transplant, surgery, or ablation). Subjects must also have at least 1 measurable lesion per Response Evaluation Criteria in Solid Tumors (RECIST) 1.1, Child-Pugh liver score A, an Eastern Cooperative Oncology Group (ECOG) performance score of 0 or 1 and predicted life expectancy of greater than 6 months. An imaging scan is required up to 21 days prior to initiation of the study treatment. Subjects are required to provide a tumor tissue sample for whole exome sequencing and personalized neoantigen vaccine development. Approximately 36 subjects will be enrolled and will receive combination therapy with the personalized neoantigen DNA vaccine (1 mg) + INO-9012 (0.34 mg) given ID via CELLECTRA® 2000 EP and pembrolizumab 200 mg IV. The total DNA vaccine dose is split between 2 doses delivered 1 after another and administered by an ID injection via CELLECTRA® 2000 EP into 2 locations (e.g., both arms; see section 8.1.2.1 and pharmacy manual).

	A key goal of this study is to determine the objective response rate (ORR) of the personalized neoantigen DNA vaccine given in combination with pembrolizumab. Beginning with screening, all imaging assessments will be evaluated using RECIST 1.1. On-study imaging assessments will be performed Q9W, calculated from the date of study treatment and independent of treatment delays. RECIST 1.1 will be used by the site for treatment decisions until the first radiologic evidence of PD.<br>Following the first radiologic evidence of PD by RECIST 1.1, treatment decisions may be made by using immune RECIST (iRECIST) to accommodate tumor response patterns seen with checkpoint inhibitor therapy including pembrolizumab treatment (e.g., tumor flare). This was first described by Nishino, et al. 2013 and is used in immunotherapy clinical studies. For a clinically stable subject with first radiologic evidence of PD, it is at the discretion of the site investigator to continue treating the subject with the personalized vaccine and pembrolizumab, until PD is confirmed, at least 4 weeks after the date of the first tumor imaging suggesting PD, per the site investigator. If radiologic PD is confirmed by the subsequent tumor imaging, the subject should be discontinued from treatment, unless in the opinion of the investigator, the subject is achieving a clinically meaningful benefit. In this case, an exception for continued treatment may be considered following consultation with the Sponsor.<br>Subjects who attain a CR by 2 tumor imaging assessments at least 4 weeks apart, and who have received approximately 6 months of therapy with the personalized neoantigen vaccine and pembrolizumab, may discontinue treatment at the discretion of the investigator after receiving at least 2 treatments beyond the initial determination of a CR. Subjects who stop GNOS-PV02 and/or pembrolizumab after receiving approximately 2 years of therapy for reasons other than PD or intolerability, or who stop after attaining a CR, may be eligible for retreatment with GNOS-PV02 + INO-9012 up to an additional 1 year of therapy Q12W (second course of treatment) after they have experienced radiographic PD. The decision to retreat will be at the discretion of the investigator, only if no other cancer treatment was administered since the last dose of GNOS-PV02 and pembrolizumab, the subject still meets the parameters listed in the inclusion and exclusion criteria, and the study remains open and there is vaccine available. Additionally, Subjects with CR, PR or SD who are still receiving treatment at the end of approximately 2 years (Week 99) are eligible to continue with GNOS-PV02 treatment every 12 weeks for approx. 5 years and every 26 weeks (q6mos) thereafter until confirmed disease progression, investigator discretion or no additional study drug available AEs will be monitored throughout the study starting at Day 0 and graded in severity according to the guidelines outlined in the National Cancer Institute (NCI) Common Terminology Criteria for Adverse Events (CTCAE) version 5.0. After the end of treatment, each subject will be followed for 30 days for AE monitoring. SAEs will be collected until the patient reaches the end of the study.<br>In addition to regularly scheduled blood sample collections, tumor tissue samples will be collected whenever clinically feasible and available: at screening, post-4 doses of vaccine (Week 9, Cycle 4, on treatment), and at time of disease progression. Also, whenever treatment-naïve archival tissue is available, it will be requested. Pre-treatment tissue sample is required and will be prioritized for tumor DNA whole exome and tumor RNA sequencing (for vaccine manufacturing). Additionally, the on-treatment tissue sample collection is strongly recommended.<br>It is anticipated that the personalized DNA vaccine will be manufactured and available for delivery in combination with pembrolizumab at the time of progression or intolerance to sorafenib or lenvatinib, or at the discretion of the treating oncologist. If patients discontinue first-line therapy and the personalized DNA vaccine is not yet available, participants may wait for the vaccine to be manufactured, or come off study to receive standard of care second-line therapy.

Research Hypothesis	GNOS-PV02 + INO-9012 delivered by ID injection, followed by EP using the CELLECTRA® 2000 device in combination with pembrolizumab, will be generally safe, well tolerated, immunogenic, and lead to anti-tumor responses in adult subjects with previously treated advanced HCC.

**Objectives and Endpoints**	
**Primary Objectives:**	**Associated Primary Endpoints:**
1. To determine the safety and tolerability of GNOS-PV02 + INO-9012 delivered by ID injection followed by EP using the CELLECTRA® 2000 device in combination with pembrolizumab	• Adverse events, as graded by CTCAE v5.0
2. To evaluate preliminary immune response to GNOS-PV02 +INO-9012 in combination with pembrolizumab in subjects with previously treated advanced HCC	• Neoantigen-specific cellular immune responses that may be assessed by but not limited to:<br>○ Interferon-γ secreting T lymphocytes in peripheral blood mononuclear cells (PBMCs) by ELISpot<br>○ T-cell activation and cytolytic cell phenotype in PBMCs by flow cytometry or secretion of immune molecules
**Secondary Objective**	**Associated Secondary Endpoints**
To evaluate the anti-tumor activity of GNOS-PV02 + INO-9012 in combination with pembrolizumab in subjects with previously treated advanced HCC	• ORR by RECIST 1.1 by investigator review<br>• ORR by iRECIST<br>• Duration of Response (DOR)<br>• Disease Control Rate (DCR)<br>• Progression Free Survival (PFS) as assessed by RECIST 1.1 and iRECIST<br>• OS
**Exploratory Objective**	**Associated Exploratory Endpoints**
1. To determine the feasibility of generating the personalized neoantigen DNA vaccine (GNOS-PV02) for subjects with advanced HCC	The feasibility assessment will be based on:<br>• Tumor-specific neoantigen identification<br>• Successful neoantigen-based DNA vaccine manufacturing<br>• The time period elapsed between tumor tissue collection and availability of the neoantigen vaccine for subject dosing
2. To evaluate tumor and immune biomarkers and their association with treatment outcome (antitumor activity and/or safety) in subjects with previously treated advanced HCC	• Assessment of myeloid derived suppressor cells (MDSC)

	• T-cell receptor (TCR) sequencing of PBMCs for diversity and putative antigen specificity<br>• Immune gene transcript profiling of PBMCs<br>• Assessment of pro-inflammatory and immunosuppressive elements in neoplastic and adjacent normal tissue, where feasible<br>• Pre- and post-treatment peripheral blood and tumor immune related gene expression.<br>• Expression of tumor-specific oncoproteins in tissue<br>• MicroRNA (miRNA) profiling to predict treatment efficacy evaluating pre- and post-treatment peripheral blood samples<br>• Circulating tumor DNA (ctDNA) analysis and tracking for progression
**Eligibility Criteria**	
**Diagnosis/Condition for Entry into the Study**	Male and female subjects with advanced HCC currently receiving or scheduled to receive first-line treatment with sorafenib or lenvatinib, will be enrolled in this study. Combination therapy with GNOS-PV02 and pembrolizumab will begin after discontinuation of first-line lenvatinib or sorafenib.
**Subject Inclusion Criteria**	In order to be eligible for participation in this study, the subject must:<br>1. Be willing and able to provide written informed consent for the study. The subject may also provide consent for Future Biomedical Research (FBR). However, the subject may participate in the main study without participating in FBR.<br>2. 18 years of age on day of signing informed consent.<br>3. Have histologically or cytologically confirmed diagnosis of HCC based on pathology report (not accepted: fibrolamellar, sarcomatoid, mixed cholangiocarcinoma). Radiological diagnosis is valid to initiate screening pending confirmation by pathology.<br>4. Have Barcelona Clinic Liver Cancer (BCLC) Stage C disease or BCLC Stage B disease not amenable to locoregional therapy, or refractory to locoregional therapy, and not amenable to a curative treatment approach.<br>5. Have a Child-Pugh Class A liver score.<br>6. Have a predicted life expectancy of greater than 6 months.<br>7. Have measurable disease based on RECIST 1.1.<br>8. Have a performance status of 0 or 1 using the ECOG Performance Scale within 7 days of first dose of study drug.<br>9. Receiving or eligible for first-line therapy with sorafenib or lenvatinib.<br>10. Willing to submit a tissue sample for personalized DNA vaccine manufacturing.<br>11. Patients with chronic or acute HBV infection [as characterized by positive hepatitis B surface antigen (HBsAg) and/or hepatitis B core antibodies (anti-HBcAb) with detectable HBV DNA (≥10 IU/ml)] must be treated with effective antiviral therapy, as per institutional practices, prior to enrollment and for the duration of the study therapy. Patients who test positive for anti-hepatitis B core (HBc) with undetectable HBV DNA (<10 IU/ml) do not require anti-viral therapy prior to enrollment; however, these subjects will be tested at every cycle to monitor

HBV DNA levels and initiate antiviral therapy if HBV DNA is detected (≥10 IU/ml). Subjects with chronic infection by hepatitis C virus (HCV), who are untreated, are allowed on study. In addition, subjects with successful HCV treatment (defined as sustained virologic response [SVR] 12 or SVR 24) are allowed, as long as 4 weeks have passed between completion of HCV therapy and start of study drug. Subjects receiving antiviral therapy during TKI may be enrolled.

12. Women of childbearing potential (WOCBP) must have a negative urine or serum pregnancy test for the patient to be eligible for trial enrolment.
13. Be willing to use an adequate method of contraception for the course of the study through 150 days after the last dose of study drug (male and female subjects of childbearing potential).
    Note: Abstinence is acceptable if this is the usual lifestyle and preferred contraception for the subject.
14. Demonstrate adequate organ function. See Table 1.

Table 1. Adequate Organ Function Laboratory Values

System	Laboratory Value
**Hematological**	
Absolute neutrophil count	≥1200/µL
Platelets	≥100,000/µL
Hemoglobin	≥8g/dL without transfusion or EPO dependency within 7 days
**Renal**	
Creatinine **OR**<br>Measured or calculated creatinine clearance<br>(GFR can also be used in place of creatinine or creatinine clearance)	≤1.5×ULN OR<br>≥60 mL/min for subject with creatinine levels<br>>1.5× institutional ULN<br>*Note: Creatinine clearance should be calculated per institutional standard*
**Hepatic**	
Total bilirubin	≤2 mg/dL, or direct bilirubin ≤ULN for those with total bilirubin >2 mg/dL
AST (SGOT) and ALT (SGPT)	≤5×ULN
Albumin	≥3.0 g/dL
**Coagulation**	

	INR or PT aPTT / ≤1.5×ULN unless subject is receiving anticoagulant therapy as long as PT or aPTT is within therapeutic range of intended use of anticoagulants
**Subject Exclusion Criteria**	The subject must be excluded from participating in the study if the subject:<br>1. Is currently participating and receiving study drug or has participated in a study of an investigational agent and received study drug or used an investigation device, within 4 weeks of the first dose of treatment. Subjects must also have recovered from associated therapy (i.e., to Grade ≤1 or baseline) and from AEs due to any prior therapy.<br>2. Has received sorafenib or lenvatinib within 14 days of first dose of study drug.<br>3. Has had esophageal or gastric variceal bleeding within the last 6 months. If suspected, subjects will be screened for esophageal varices. If varices are present, they should be treated according to institutional standards before starting study treatment.<br>4. Has clinically apparent ascites on physical examination. Note: only ascites detectable on imaging studies is allowed.<br>5. Evidence of portal vein invasion based on imaging is allowed pending subjects meet laboratory criteria for enrollment.<br>6. Has had encephalopathy in the last 6 months. Subjects on rifaximin or lactulose to control their encephalopathy are not allowed.<br>7. Had a solid organ or hematologic transplant.<br>8. Had prior systemic therapy for HCC other than sorafenib or lenvatinib.<br>9. Has active autoimmune disease that has required systemic treatment in past 2 years (i.e., with use of disease-modifying agents, corticosteroids, or immunosuppressive drugs). Replacement therapy (e.g., T4, insulin, or physiologic corticosteroid replacement therapy for adrenal or pituitary insufficiency, etc.) is not considered a form of systemic treatment.<br>10. Has a diagnosis of immunodeficiency or is receiving systemic steroid therapy or any other form of immunosuppressive therapy within 7 days prior to the first dose of study drug. The use of physiologic doses of corticosteroids may be approved after consultation with the Sponsor.<br>11. Has received locoregional therapy to liver (transarterial chemoembolization [TACE], transarterial embolization [TAE], radiation, radioembolization, or ablation) or major surgery to liver or other site within 3 weeks prior to the first dose of study drug. Minor surgery (e.g., simple excision, tooth extraction) must have occurred at least 7 days prior to the first dose of study drug (Cycle 1, Day 1). Subjects must have recovered adequately (i.e., Grade ≤1 or baseline) from the toxicity and/or complications from any intervention prior to starting therapy.<br>12. Has a diagnosed additional malignancy within 5 years prior to first dose of study drug, with the exception of: (a) non-invasive carcinomas subject to successful curative treatment in the opinion of the investigator which require no further therapy and (b) other malignancies for which patients have undergone potentially curative therapy and have been considered disease free for at least 3 years prior to screening.

	13. Has radiographically detectable (even if asymptomatic and/or previously treated) central nervous system (CNS) metastases and/or carcinomatous meningitis, as assessed by local site investigator.<br>14. Has a known history of, or any evidence of, interstitial lung disease or active non-infectious pneumonitis.<br>15. Has an active infection requiring systemic therapy.<br>16. Has a history or current evidence of any condition, therapy, or laboratory abnormality that might confound the results of the study, interfere with the subject's participation for the full duration of the study, or is not in the best interest of the subject to participate, in the opinion of the treating investigator, including dialysis.<br>17. Has known psychiatric or substance abuse disorders that would interfere with cooperation with the requirements of the study.<br>18. Is pregnant or breastfeeding or expecting to conceive or father children within the projected duration of the study, starting with the screening visit through 150 days after the last dose of study drug.<br>19. Has received prior immunotherapy including anti-programmed death (PD)-1, anti-programmed death ligand (PD-L)-1, or anti–PD-L2 agents, or personalized therapies such as adoptive cell therapy or neoantigen-based vaccine. Note: oncolytic viruses are not considered immunotherapy.<br>20. Has a known history of human immunodeficiency virus (HIV) (HIV I/II antibodies).<br>21. Has untreated active hepatitis B virus (HBV), unless planned antiviral therapy during TKI.<br>Note: Patients with HBV infection, characterized by positive HBsAg and/or HBcAb with detectable HBV DNA (≥10 IU/ml or above the limit of detection per local lab standard), must be treated with antiviral therapy as per institutional practice to ensure adequate viral suppression (HBV DNA ≤2000 IU/mL) prior to treatment with the study drug. Patients who test positive for HBcAg with undetectable HBV DNA (<10 IU/ml or under the limit of detection per local lab standard) do not require anti-viral therapy prior to enrollment.<br>22. Has received a live vaccine within 30 days of planned start of study treatment (Cycle 1, Day 1).<br>Note: The killed virus vaccines used for seasonal influenza vaccines for injection are allowed; however, intranasal influenza vaccines (e.g., FluMist®) are live attenuated vaccines and are not allowed. Non-live COVID-19 vaccines are allowed.<br>23. Any contraindication for treatment with the CELLECTRA® 2000 Device:<br>Less than two acceptable sites available for ID injection and EP considering the deltoid and anterolateral quadriceps muscles:<br>a. Tattoos, keloids or hypertrophic scars located within 2 cm of intended administration site.<br>b. Implantable-Cardioverter-defibrillator (ICD) or pacemaker (to prevent a life-threatening arrhythmia) that is located ipsilateral to the deltoid injection site (unless deemed acceptable by a cardiologist).

	c. Any metal implants or implantable medical device within the intended treatment site (i.e. electroporation area).<br>24. Has no mutations detected after sequencing of the tumor.
**Study Treatments**	

The treatments to be used in this study is outlined below in Table 2.

Table 2. Study Treatments

Drug	Dose	Dose Frequency	Route of Administration	Regimen	Use
Personalized Neoantigen DNA Vaccine + plasmid encoded IL-12 (GNOS-PV02 + INO-9012)	1 mg GNOS-PV02 + 0.34mg INO-9012	Q3W (Cycles 1-4), Q9W thereafter	ID injection followed by CELLECTRA® 2000 EP	Day 1 of each 21-day cycle for Cycles 1-4, Q9W thereafter	Experimental
Pembrolizumab (MK-3475)	200 mg	Q3W	IV Infusion	Day 1 of each 21-day cycle	Standard of Care

Table 3. Schedule of Assessments (SOA)

Visit	Pre-study[a]	Day 0	Every 3 Weeks	Every 9 Weeks[w]	Every 12 Weeks[v] (post 24 mo)	Every 26 Weeks[v] (post 5 yrs)	Treatment Discontinuation Visit[b]	Follow-up (Every 12 Weeks)
**Visit Window (Days)**		±3	±3	±7	±7	±7	±3	±7
Signed ICF(s)[a]	X							
Medical, surgical, and cancer histories, including demographic information[c]	X	X						
Full Inclusion/Exclusion Criteria	X							
Abbreviated Inclusion/ Exclusion Criteria		X						

Visit	Pre-study[a]	Day 0	Every 3 Weeks	Every 9 Weeks[w]	Every 12 Weeks[v] (post 24 mo)	Every 26 Weeks[v] (post 5 yrs)	Treatment Discontinuation Visit[b]	Follow-up (Every 12 Weeks)
**Visit Window (Days)**		±3	±3	±7	±7	±7	±3	±7
Complete physical exam[d]	X	X						
Targeted physical exam[e]			X[f]				X	
ECOG performance status[f]	X	X	X				X	
HIV, HBV and HCV serology[g]	X							
Concomitant medications[h]	X	X	X				X	
Vital signs and weight[i]	X	X	X				X	
Height	X							
12-lead ECG[j]	X							
Alpha-fetoprotein (AFP)	X	X	X					
Tumor measurements[k]		X[k]		X	X	X		
Hematology[l]	X	X[f]	X[f]					
Serum chemistry[m]	X	X[f]	X[f]					
Coagulation panel (aPTT, INR)	X	X						
Pregnancy[n]	X	X	X				X	
TSH, T3 and Free T4[o]	X	X	X					
60 mL blood samples for immunology assessments[p]	X		After Day 0, every 3 weeks until week 12, then every 9 weeks		X	X		
120 mL blood samples for research[p]		X	Week 12 mandatory and at first evidence of disease progression if feasible				X	

Visit	Pre-study[a]	Day 0	Every 3 Weeks	Every 9 Weeks[w]	Every 12 Weeks[v] (post 24 mo)	Every 26 Weeks[v] (post 5 yrs)	Treatment Discontinuation Visit[b]	Follow-up (Every 12 Weeks)
**Visit Window (Days)**		±3	±3	±7	±7	±7	±3	±7
Blood samples for ctDNA[p]		X	Every 3 weeks until week 12, then every 9 weeks		X	X	X	
Tumor specimen[q]	X		9 weeks after first GNOS-PV02 dose (mandatory if clinically feasible), and at first evidence of disease progression (optional)					
Adverse events[r]		X	X		X	X	X	
Pembrolizumab[s]		X	X					
GNOS-PV02 + INO-9012[t]		X	Every 3 weeks for Cycles 1-4 then every 9 weeks until week 99, then every 12 weeks for 5 years until PD			X		
Survival and new anti-cancer therapy follow-up[u]								X

Each Cycle= 21 days, assessments scheduled on the days of study treatment should be performed before the study treatment unless otherwise noted.

[a] Written informed consent can be obtained up to 28 days prior to performing any study-specific tests or procedures. Tumor tissue for personalized vaccine manufacturing should be submitted within 28 days of signing informed consent. Results of standard-of-care tests or examinations performed prior to obtaining informed consent and within 28 days of signing informed consent may be used for screening assessments rather than repeating such tests.

[b] Off-study evaluations will be completed within 30 days of discontinuing study therapy.

[c] Cancer history includes stage, date of diagnosis, and prior anti-tumor treatment. Previous progression data should be collected as well. Demographic information includes sex, age, and self-reported race/ethnicity. HIV, HBV, HCV and reproductive status and smoking/alcohol history should also be captured.

[d] A complete physical exam will include head, eyes, ears, nose, throat and cardiovascular, dermatological, musculoskeletal, respiratory, gastrointestinal and neurological systems. Height and weight will also be collected. Any signs and symptoms, other than those associated with a definitive diagnosis, should be collected at Day 0 and during the study.

[e] A targeted, symptom-directed exam will be performed, as clinically indicated.

[f] ECOG performance status, targeted physical exam, and local laboratory assessments may be obtained ≤72 hours before each dosing visit.

[g] Subjects should be tested for HIV locally prior to the inclusion into the study only based on investigator’s clinical suspicion for HIV infection and HIV-positive subjects will be excluded from the clinical study. Subjects who are HBsAg positive, in order to qualify for enrollment, must receive antiviral therapy during TKI therapy, and HBV

viral load must be less than 2000 IU/mL prior to first dose of study drug. Subjects receiving antiviral therapy during TKI may be enrolled. Those on active HBV therapy with viral loads under 2000 IU/mL should stay on the same therapy throughout study treatment. Those subjects who are anti-HBc (+), and negative for HBsAg, and negative for anti-HBs, and have an HBV viral load under 100 IU/mL do not require HBV anti-viral prophylaxis but need close monitoring. These subjects will be tested at every cycle to monitor HBV DNA levels and initiate antiviral therapy if HBV DNA is detected (≥10 IU/ml or above the limit of detection per local lab standard).

[h] Concomitant medications include any prescription medications or over-the-counter medications. At screening, any medications the subject has used within the 7 days prior to the screening visit should be documented. At subsequent visits, changes to current medications or medications used since the last documentation of medications will be recorded.

[i] Vital signs include heart rate, respiratory rate, blood pressure, and temperature. For first infusion of pembrolizumab, the subject's vital signs should be determined within 60 minutes before the infusion. If clinically indicated, vital signs should be at 15, 30, 45, and 60 minutes (± 5 minutes for all time points) after the start of the infusion, and 30 (± 10) minutes after the infusion. For subsequent infusions, vital signs will be collected within 60 minutes before the infusion and at 30 (± 5) minutes after the infusion. Subjects will be informed about the possibility of delayed post-infusion symptoms and instructed to contact their study physician if they develop such symptoms.

[j] ECG recordings will be obtained during screening and as clinically indicated at other time points. Subjects should be resting and in a supine position for at least 10 minutes prior to ECG collection.

[k] Baseline scans must be done < 3 weeks prior to C1D0 of pembrolizumab and GNOS-PV02 administration. Tumor measurement will be performed with contrast CT chest/abdomen/pelvis. Non-contrast CT chest/abdomen/pelvis or CT chest and MRI abdomen/pelvis can be performed as an alternative to contrast CT, and the same imaging technique should be used in patient throughout the study. Imaging will be repeated every 9 weeks (+/-7 days), irrespective of dosing schedule or treatment delays. If patients remain on study for 54 weeks, imaging may be extended to every 12 weeks (+/- 7 days). Patients who continue treatment beyond radiographic or clinical disease progression will be monitored with a follow-up scan at the next scheduled tumor assessment. Confirmatory scans are requested in the 4-6 weeks timeframe after the first radiographic evidence of PR/CR. After the confirmatory scan is obtained, standard imaging schedule will resume (i.e. Q9W)

[l] Hematology consists of complete blood count, including red blood cell count, hemoglobin, hematocrit, white blood cell count with automated differential (absolute counts of neutrophils, lymphocytes, eosinophils, monocytes, basophils, and other cells [if any]), and platelet count. A manual differential can be done if clinically indicated.

[m] Serum chemistry includes BUN or urea, creatinine, sodium, potassium, magnesium, chloride, bicarbonate or CO2, calcium, phosphorus, glucose, total bilirubin (direct bilirubin only if total bilirubin is elevated), ALT, AST, alkaline phosphatase, lactate dehydrogenase, total protein, and albumin.

[n] Pregnancy test (for women of childbearing potential, including women who have had a tubal ligation) must be performed and documented as negative within 72 hours prior to each dose. Urine test is valid if negative or positive result. Serum test is required if urine is indeterminate.

[o] Thyroid function tests will be performed at screening and Q3W from Day 0. Free T3 can be used as an alternative if T3 is not available.

[p] Immunology/research/ctDNA samples are to be drawn at screening (60 mL+ 3mL for sequencing), at Day 0 (130 mL), Week 3 (70 mL), Week 6 (70 mL), Week 9 (70 mL), at Week 12 (130 mL), Q9W (70 mL) until week 99, then Q12W (70 mL) until disease progression, as well as at disease progression (130 mL, if feasible).

[q] A pre-treatment tumor tissue sample is required and will be prioritized for tumor DNA whole exome and tumor RNA sequencing (for vaccine manufacturing). After signing of the ICF, tumor tissue should be submitted to the Sponsor as soon as possible. Tumor tissue should be of good quality based on total and viable tumor content. Acceptable samples include core needle biopsies for deep tumor tissue or lymph nodes or excisional, incisional, punch, or forceps biopsies for cutaneous, subcutaneous, or mucosal lesions. Fine-needle aspiration may be acceptable pending Sponsor approval; however, brushing, cell pellets from pleural effusion, and lavage samples are not acceptable. For core needle biopsy specimens, at least 3 cores should be submitted for evaluation. A new biopsy will be obtained for subjects without sufficient archival tissue. All subjects will undergo a mandatory tumor biopsy

sample collection, if clinically feasible as determined by the study investigator, after 4 doses of GNOS-PV02 + INO-9012 and at the first evidence of radiographic or clinical disease progression (i.e., not proceeded by meaningful tumor regression). For subjects who respond and subsequently progress, an optional biopsy may be obtained at the time of disease progression. Subjects who are unable to undergo biopsy sample collection, but otherwise meet criteria outlined in protocol, may continue to receive study drug. Also, whenever treatment-naïve archival tissue is available, it will be requested.

[r] AEs will be collected from the time of therapy initiation with pembrolizumab and GNOS-PV02+INO-9012, whichever occurs first. SAEs will be collected from the time of therapy initiation with pembrolizumab and GNOS-PV02+INO-9012, whichever occurs first. AEs that occur during the administration of standard of care TKI therapy will not be collected.

[s] Pembrolizumab will be given Q3W until confirmed disease progression, unacceptable toxicity, deemed intolerable by investigator, or up to 24 months in subjects without disease progression. The window for each visit is ±3 days unless otherwise noted. A 60-minute observation period is recommended for the first dose and 30 minutes for subsequent doses.

[t] GNOS-PV02 + INO-9012 is administered Q3W for 4 doses (dose 1-4). After the first 4 doses, GNOS-PV02+INO-9012 will be administered Q9W until confirmed disease progression, unacceptable toxicity, deemed intolerable by investigator or up to 36 months in subjects without disease progression. Continued treatment with GNOS-PV02+INO-9012 after 2-years will be administered Q12W. The window for each visit is ±3 days unless otherwise noted. A 30-minute observation period is recommended for each subsequent dose.

[u] Survival follow-up information will be collected via telephone calls, subject medical records, and/or clinical visits approximately Q12W for up to 3 years, until death, lost to follow-up, withdrawal of consent, or study termination by Sponsor. All subjects will be followed for survival and new anticancer therapy information unless the subject requests to be withdrawn from follow-up; this request must be documented in the source documents and signed by the investigator. If the subject discontinues study drug without documented clinical disease progression, every effort should be made to follow up regarding survival, progression (if not already progressed), and new anti-cancer therapy initiation.

[v] Patients with CR, PR or SD who are still receiving treatment at the end of approximately 2 years (Week 99) are eligible to continue with GNOS-PV02 treatment every 12 weeks for approx. 5 years and every 26 weeks (q6mos) thereafter until confirmed disease progression, investigator discretion or no additional study drug

[w] Patients who have progressed on a non-biopsy lesion have the option to have another vaccine manufactured with sponsor approval and continue treatment until year 2. Patients will continue to follow the study assessments as noted above.

2. TERMS, ACRONYMS, ABBREVIATIONS

Abbreviation/Term	Definition
ADL	Activities of daily living
AE	Adverse event
ALT	Alanine aminotransferase
aPTT	Activated partial thromboplastin time
AST	Aspartate aminotransferase
BCLC	Barcelona Clinic Liver Cancer
CD	Cluster of differentiation
cDNA	Complementary deoxyribonucleic acid
cGMP	Current Good Manufacturing Practice
CI	Confidence interval
CMV	Cytomegalovirus
CPI	Checkpoint inhibitor
CR	Complete response
CRF	Case report form
CT	Computed tomography
CTCAE	Common Terminology Criteria for Adverse Events
ctDNA	Circulating tumor DNA
CTL	Cytotoxic T-lymphocyte
CTLA-4	Cytotoxic T-lymphocyte–associated antigen-4
DCR	Disease control rate
DLT	Dose-limiting toxicity
DNA	Deoxyribonucleic acid
DOR	Duration of response
ECG	Electrocardiogram
ECOG	Eastern Cooperative Oncology Group
eCRF	Electronic case report form
EP	Electroporation
EPO	Erythropoietin
FBR	Future biomedical research
FDA	Food and Drug Administration
FFPE	Formalin-fixed paraffin embedded
GFR	Glomerular filtration rate
GLP	Good laboratory practice
HBsAg	Hepatitis B surface antigen

Abbreviation/Term	Definition
HBV	Hepatitis B virus
HCC	Hepatocellular carcinoma
HCV	Hepatitis C virus
HIV	Human immunodeficiency virus
HLA	Human leukocyte antigen
HR	Hazard ratio
IB	Investigator's Brochure
ICF	Informed consent form
ICH	International Council for Harmonization
iCR	Complete response by iRECIST
ID	Intradermal
IEC	Independent Ethics Committee
IFN	Interferon
IgG	Immunoglobulin G
IL-12	Interleukin 12
IM	Intramuscular
IND	Investigational New Drug
INR	International normalized ratio
IP	Investigational product
iPR	Partial response by iRECIST
IRB	Institutional Review Board
iRECIST	Immune Response Evaluation Criteria in Solid Tumors
iSD	Stable disease by iRECIST
iUPD	Unconfirmed progressive disease by iRECIST
IV	Intravenous
MDSC	Myeloid derived suppressor cells
MHC	Major histocompatibility complex
MRI	Magnetic resonance imaging
mRNA	Messenger ribonucleic acid
NE	Not evaluable
NLT	New lesion target
ORR	Objective response rate
OS	Overall survival
PBMC	Peripheral blood mononuclear cell
PD	Progressive disease

Abbreviation/Term	Definition
PD-1	Programmed cell death protein 1
PD-L1	Programmed cell death protein-ligand 1
PET	Positron emission tomography
PFS	Progression free survival
PP	Per protocol
PR	Partial response
Q3W	Every 3 weeks
Q9W	Every 9 weeks
Q12W	Every 12 weeks
Q26W	Every 26 weeks
RECIST	Response Evaluation Criteria in Solid Tumors
RNA	Ribonucleic acid
SAE	Serious adverse event
SARS	Severe acute respiratory syndrome
SD	Stable disease
SGOT	Serum glutamic-oxaloacetic transaminase
SGPT	Serum glutamic-pyruvic transaminase
SIV	Simian immunodeficiency virus
SSC	Saline sodium citrate
SynCon®	Synthetic Consensus
T3	Triiodothyronine
T4	Thyroxine
TACE	Transarterial chemoembolization
TCR	T-cell receptor
TEAE	Treatment-emergent adverse event
TKI	Tyrosine kinase inhibitor
TNF-α	Tumor necrosis factor-alpha
TSH	Thyroid stimulating hormone
UADE	Unanticipated Adverse Devise Effect
ULN	Upper limit of normal
US	United States
WFI	Water for injection

3. INTRODUCTION

3.1. Background

Refer to the Investigator's Brochure (IB) V1.0 for more details on GNOS-PV02. For more details on pembrolizumab, refer to the package insert https://www.merck.com/product/usa/pi_circulars/k/keytruda/keytruda_pi.pdf.

3.2. Rationale

Anti-programmed cell death protein 1 (PD-1) antibodies including nivolumab and pembrolizumab can induce durable remissions in 13%–16% of patients with advanced hepatocellular carcinoma (HCC), but the majority of patients fail to respond due to inadequate intra-tumoral immune cell infiltration. It is hypothesized that these tumors are generally immunologically "cold," or T-cell excluded for various reasons. Even if there is intra-tumoral T-cell infiltration, these T-cells are not able to exert a significant anti-tumor effect, likely owing to the suppressive tumor microenvironment. Neoantigens are expressed in advanced HCC, but de-novo responses to these neoantigens may be limited due to immune evasion. Our hypothesis is that GNOS-PV02 + INO-9012 will help generate functional, activated CD8+ T-cells, which when combined with a checkpoint inhibitor (CPI) will lead to improved clinical outcomes. Geneos Therapeutics plans to develop GNOS-PV02 + INO 9012 in combination with pembrolizumab in a study targeting patients with advanced HCC which is a population with high unmet medical need. The overall goal is to improve clinical outcomes in patients who are eligible to receive CPI therapy.

3.2.1 Rationale for the Study and Selected Subject Population

Immune checkpoint blockade, specifically those targeting the Cytotoxic T Lymphocyte–associated antigen-4 (CTLA-4) and PD-1/PD-L1 pathways, has shown efficacy in multiple solid and hematologic malignancies. Furthermore, as has been demonstrated in metastatic melanoma, combined CTLA-4 and PD-1/PD-L1 blockade has shown improved objective response rate (ORR) and overall survival (OS), though there is a significant increase in serious immune-related adverse events (AEs). As such, current studies are exploring different doses, administration schedules, and immune checkpoint agents. One alternative approach, however, is to introduce a tumor-directed therapy such as a personalized neoantigen vaccine combined with these immune modulating agents (i.e., immune checkpoint blocking antibodies) to maximize the tumor specific response but minimize the toxicity associated with increasing non-specific systemic immune activation by generating a potent and focused neoantigen specific immune response.

Justification for the synergistic effect of a vaccine combined with immune checkpoint blockade has been demonstrated preclinically in various tumor models.

Clinically, various vaccine strategies, including autologous or allogeneic tumor lysate, as well as peptides against shared tumor antigens, have been combined with either CTLA-4 or PD-1 blockade therapy in melanoma, pancreatic adenocarcinoma, and prostate cancer.

To date, however, no clinical efficacy data has been obtained from HCC studies in patients combining vaccination with PD-1/PD-L1 blockade therapy. It is hypothesized that GNOS-PV02 + INO-9012, given its biologic properties and HCC-specific uniquely personalized neoantigen targets, can impact on biologically relevant cancer targets in a differentiated and meaningful manner. Accordingly, the Sponsor seeks now to generate initial signals of the impact of GNOS-PV02 + INO-9012 in oncologic investigational spaces of high unmet medical need, including advanced HCC.

3.2.2 Unmet Medical Need for Patients with Advanced HCC

HCC is the sixth most common cancer worldwide, with over 700,000 annual diagnoses, and is the fourth most common cause of cancer-related death worldwide. Fifty-five percent of HCC cases occur in China;

however, the incidence is rising in the United States (US) and Europe. (1) In most patients, HCC arises in conjunction with liver cirrhosis and is attributed to several risk factors, including infection (usually with Hepatitis B Virus [HBV] or Hepatitis C Virus [HVC]), excessive alcohol consumption, and non- alcoholic fatty liver disease. Currently, HCC management for patients with early-stage disease relies on a multidisciplinary approach with the first step being the multidisciplinary team evaluation, followed by assessment for curative treatment options such as resection, transplantation, or radiofrequency ablation. Institutional expertise of regional therapy such as transarterial chemoembolization (TACE), radioembolization, and radiation also play a role in the treatment of early-stage disease. Most patients however present with advanced, unresectable disease. These patients are typically treated worldwide with systemic therapies.

Tyrosine kinase inhibitors (TKIs) are indicated for first-line treatment, followed by CPIs as second-line treatment. Patients are also encouraged to participate in clinical studies because of the lack of treatment options. For advanced HCC, the standard of care in first-line therapy are the TKIs lenvatinib or sorafenib. In second-line therapy, the CPIs pembrolizumab and nivolumab, as well as other TKIs regorafenib, cabozantinib, and ramucirumab are approved.

Two separate large, randomized studies have evaluated OS of sorafenib in advanced HCC: the Sorafenib Hepatocellular Carcinoma Assessment Randomized Protocol (SHARP)(2) vs Asia-Pacific Study(3), which led to approval of sorafenib in first-line therapy. In the SHARP trial, subjects receiving sorafenib vs placebo had an OS of 10.7 vs 7.9 months and a hazard ratio (HR) of 0.69 (0.55–0.87). In the Asia-Pacific trial, subjects receiving sorafenib vs placebo had an OS of 6.5 vs 4.2 months and an HR of 0.68 (0.50–0.93).

The REFLECT trial was a global, randomized, open-label, phase III noninferiority study which evaluated lenvatinib vs sorafenib as first-line treatment in patients with unresectable HCC. The study enrolled 954 subjects in a 1:1 randomization. Subjects had no prior systemic therapy for unresectable HCC, ≥1 measurable target lesion based on modified Response Evaluation Criteria in Solid Tumors (RECIST), Barcelona Clinic Liver Cancer (BCLC) Stage B or C, Child-Pugh Class A, Eastern Cooperative Oncology Group (ECOG) performance score ≤1, and adequate organ function. Subjects with ≥50% liver occupation, clear bile duct invasion, or portal vein invasion at the main portal vein were excluded. No difference in OS was observed (13.6 vs 12.3 months). ORR was significantly higher in the lenvatinib arm, confirmed on blinded central review. Progression free survival (PFS; lenvatinib 7.4 vs sorafenib 3.7 months), HR (0.66), and ORR (lenvatinib 24.1% vs sorafenib 9.2%) were superior for lenvatinib ($p<0.001$). Lenvatinib met pre-specified non-inferiority margin of 95% confidence interval (CI) upper limit being <1.08. Treatment-emergent adverse event(s) (TEAEs) were generally similar between the two arms, with the exception of higher incidence of grade 3/4 incidence of hypertension in the lenvatinib arm and higher incidence of grade 3/4 of palmar-plantar erythrodysesthesia. Subsequently, lenvatinib was approved in the first-line setting.

CELESTIAL was a phase III study of cabozantinib vs placebo which enrolled 760 subjects in a 2:1 randomization ratio. The population included subjects with advanced HCC who had received prior sorafenib and had <2 prior systemic therapies. OS was 10.2 months in the cabozantinib arm vs 8.0 on the placebo arm.

The REACH-2 study enrolled 278 subjects with a baseline alpha-fetoprotein >400ng/ml and randomized subjects 2:1 between ramucirumab and placebo. Median OS was 6.7 months with ramucirumab vs 4.5 months with placebo.

The RESORCE trial evaluated regorafenib vs placebo, enrolling 573 subjects with a 2:1 randomization ratio. Regorafenib was administered 160 mg orally with 3 weeks on, 1 week off (4-week cycle). The study enrolled HCC subjects with documented radiologic progression during sorafenib treatment. The subjects were stratified by geographic region (Asia vs rest of the world), macrovascular invasion, extrahepatic

disease, ECOG performance score (0 vs 1) and alpha-fetoprotein (< or ≥400 ng/mL). All subjects received best supportive care and subjects were treated until disease progression, death, or unacceptable toxicity. Median OS was 10.6 months in the regorafenib arm vs 7.8 months (HR 0.63 [0.50-0.79]; p<0.0001 [2-sided)]) in the placebo arm. Other statistically significant endpoints were PFS (3.1 vs 1.5 months, HR = 0.46, p<0.001), time to progression (3.2 vs 1.5 months HR = 0.44, p<0.001), ORR (10.6% vs 4.1%, p=0.005), and disease control rate (DCR; 65.2% vs 36.1%, p<0.001).

Recently, immunotherapy with checkpoint blockade inhibitors has shown promising preliminary results in subjects with advanced HCC, either as monotherapy or combination therapy. The anti-PD-1 inhibitor nivolumab has shown encouraging outcomes and a safety profile that is generally consistent with that reported in other tumor types, and as such, has received accelerated approval in the US for the treatment of HCC that has previously been treated with sorafenib. In the Checkmate 040 trial, long-term survival was observed across sorafenib-naive and previously treated cohorts. Pembrolizumab, an anti-PD-1 monoclonal antibody, has shown antitumor activity, and a manageable safety profile in many cancers, including melanoma, non-small cell lung cancer, head and neck cancer, squamous cell carcinoma, gastric, urothelial, cervical cancers, and classical Hodgkin's lymphoma, leading to many approvals in first-line and second-line settings.

The KEYNOTE-224 trial was a nonrandomized, multicenter, open-label, phase II trial conducted in 47 centers in 10 countries, and it enrolled 104 subjects who received 200 mg pembrolizumab IV (intravenous) every 3 weeks (Q3W) for 2 years or until disease progression. At the time of data cutoff on February 13, 2018, 17 (16%) subjects were still under treatment with pembrolizumab. The median duration of follow-up was 12.3 months (interquartile range 7.6–15.1). Of 104 subjects, 18 achieved an objective response, and the ORR was 17% (95% CI 11–26). Forty-six subjects (44%) had stable disease (SD) and 34 subjects (33%) had progressive disease (PD).

The median time to response was 2.1 months (interquartile range 2.1–4.1). Of 18 responders, 12 (67%) reached a response at the time of the first imaging examination for efficacy during Weeks 8–10. The median response duration was not reached (range 3.1–14.6+) and the response duration was favorable (i.e., ≥9 months in 12 [77%] subjects). At the time of data cutoff, 84 subjects (81%) had experienced death or PD, and the median PFS was 4.9 months (95% CI 3.9–8.0). Sixty subjects (58%) died before data cutoff, and the median OS was 12.9 months (95% CI 9.7–15.5).(4)

Based on results of the KEYNOTE-224 trial, pembrolizumab was the second CPI to be approved in advanced HCC, after prior progression or intolerance to a TKI.

KEYNOTE-240 was a phase III, randomized, global trial between pembrolizumab and placebo designed to detect a survival advantage for subjects receiving pembrolizumab as second-line therapy. In the final analysis of the KEYNOTE-240 study, there was an improvement in OS for subjects treated with pembrolizumab compared with placebo, but these OS results did not meet statistical significance per the statistical plan. Results for PFS were also favorable in the pembrolizumab arm compared with placebo but did not reach the pre-specified statistical significance.

Table 4, adapted from Kudo (5) summarizes available clinical data with CPIs as monotherapy and combination therapy in advanced HCC.

Table 4. Results of Immune Checkpoint Inhibitors and Combination Therapy

	Nivolumab (n=214)	Pembrolizumab (n=104)	Pembrolizumab + lenvatinib (n=26)	Atezolizumab +bevacizumab (n=73)	SHR-1210 +apatinib (n=18)	Durvalumab + tremelimumab (n=40)
ORR (95% CI)	20% (15-26)	17% (11-26)	53.3% (34.3-71.7)	32%	38.9%	25%
DCR (95% CI)	64% (58-71)	62% (52-71)	90%	77%	83.3%	57.5% (>16 weeks)
PFS, months (95% CI)	4.0 (2.9-5.4)	4.9 (3.4-7.2)	9.7 (7.7-NE)	14.9 (0.5-21.5)	7.2 (2.6-NE)	NA
OS, months (95% CI)	NR[a]	12.9 (9.7-15.5)	14.6 (9.9-NE)	NR	NR	NA
DOR, months	9.9 (8.3-NE)	≤9 (77%)	8.3 (3.8-11.0)	≥12 (26%)	NE	NA

DCR = disease control rate; DOR = duration of response; NA = not available; NE = not evaluable; NR = not reached; ORR = objective response rate (RECIST 1.1); OS = overall survival; PFS = progression free survival.

[a]Nine months: 74%

A combination phase III study of pembrolizumab and lenvatinib is ongoing; however, previously, the updated results of an open-label phase Ib study of combination therapy with pembrolizumab and lenvatinib (6) were presented at a medical conference in early 2019. Thirty patients were evaluated for safety and efficacy; the response rate was 60.0%, and the disease control rate (DCR) was 93.3%, per modified RECIST, by independent image review. According to RECIST 1.1, the response rate was 53.3% and the DCR was 90.0%, which are the best results of PD-1/PD-L1 antibody-based monotherapy or combination therapy with CTLA-4 antibody or other TKI/anti-vascular endothelial growth factor antibody therapy obtained to date. PFS was also a favorable 9.7 months. Based on these results, the pembrolizumab + lenvatinib first-line therapy received breakthrough therapy designation and the results of the phase III trial are eagerly awaited.

The liver maintains a balance between activation of, and tolerance by, immune cells in response to antigenic hyperstimulation, and dysregulation of this tightly controlled immunological network leads to chronic liver disease and HCC. HCC has been shown to be associated with inflammation and a suppressed immune environment. An inflammatory gene-expression signature was shown to be predictive of lower OS in liver tissue adjacent to tumors in subjects with HCC, and high expression of PD-L1 in tumors correlates with a poorer prognosis than lower expression of PD-L1 in subjects with resected HCC. Furthermore, upregulation of PD-1 and PD-L1 expression on T-cells is also associated with a more advanced disease stage and higher recurrence rates in subjects with HCC. Signature genes in subsets of infiltrating regulatory T-cells and exhausted CD8+ cells of subjects with HCC have been identified, including layillin, which might be linked to immune suppression by these cells. These immunological findings further suggest that immunotherapy approaches could benefit these subjects.

Geneos Therapeutics plans to evaluate GNOS-PV02 + INO-9012 in combination with pembrolizumab in subjects with advanced HCC who progress or are intolerant of TKIs.

3.2.2.1 Neoantigens in Cancer

Tumor antigens are often classified as shared tumor antigens and tumor-specific antigens. The majority of tumor-specific antigens are now believed to be the result of somatic mutations present in the tumor. Shared tumor antigens are expressed in multiple cancers and are often self-differentiation antigens that are expressed in a limited subset of normal tissues but overexpressed in cancers. Examples of shared tumor antigens include MAGE (melanoma-associated antigen), prostatic acid phosphatase (prostate cancer), and human epidermal growth factor receptor 2/neu (breast cancer). On the other hand, tumor-specific antigens, or neoantigens, are uniquely expressed in individual cancers and are typically the result of point mutations or other genetic changes that are present only in the tumor (7). As such, tumor-specific antigens represent the only antigens that are truly unique to the tumor and not expressed in normal tissues. The first human mutant tumor-specific antigen was described in 1995, resulting from a point mutation of cyclin-dependent kinase 4(8). Since that time, additional publications have described the expression of mutant tumor-specific antigens in melanoma, non-small cell lung cancer and other human cancers.

Cancer vaccine strategies targeting mutant tumor-specific antigens have scientific advantages over strategies targeting shared tumor antigens. These potential advantages include: (1) Targeting mutant tumor specific antigens is potentially safer. Mutant tumor-specific antigens are expressed only in the tumor, decreasing the risk of autoimmunity. (2) Targeting mutant tumor-specific antigens is potentially more effective. T-cell responses to mutant tumor-specific antigens are high in affinity and are not limited by central mechanisms of self-tolerance. (3) Targeting mutant tumor-specific antigens potentially limits antigen-loss, a common tumor escape mechanism. One of the hallmarks of cancer is genome instability, and one clear weakness of cancer vaccines that target a single shared tumor antigen is antigen-loss. Targeting multiple mutant tumor-specific antigens may preclude antigen loss. In addition, many mutant tumor-specific antigens play a functional role in neoplastic transformation (driver mutations). Immune selection resulting in loss of driver mutations may fundamentally alter the phenotype of targeted cancers. (4) Targeting mutant tumor-specific antigens is likely to be universally applicable in solid tumors. Solid tumors appear to have a remarkable number of nonsynonymous mutations present (each nonsynonymous mutation is a candidate mutant tumor-specific antigen), suggesting that a personalized vaccine approach could be used in most solid tumor subjects, regardless of intrinsic subtype or human leukocyte antigen (HLA) type.

3.2.2.2 Immunogenomics: A Sequencing-based Strategy to Identify Neoantigens

Cancer genome sequencing is the first step in the tumor neoantigen identification effort. Cancer immunogenomics is a strategy that uses the information obtained from these DNA and RNA sequencing efforts to identify expressed subject tumor-specific mutations, or neoantigens. Neoantigens are then pipelined through various readily available in silico HLA binding prediction algorithms to identify candidate neoantigens that bind with high affinity to a subject’s individual HLA alleles. As such, robust next-generation sequencing strategies and computational programs for the identification of mutant tumor specific antigens will be required for the successful clinical translation of personalized cancer vaccine strategies. Therefore, a major focus of research studies has been the development of cost-effective and accurate next-generation sequencing strategies to identify mutant tumor-specific antigens and validate the expression of these antigens at the mRNA (messenger ribonucleic acid) level. Initially, a cancer genome sequencing approach was used. While cancer whole genome sequencing is informative and provides comprehensive information about both the coding and noncoding regions of the genome, this level of information may not be necessary for identifying mutant tumor-specific antigens, or prioritizing antigens for immune intervention. It has been demonstrated that tumor/normal exome sequencing is a robust and accurate strategy for the identification of mutant tumor-specific antigens (9). Of note, recent studies suggest that approximately 40% of mutations identified by cancer exome sequencing are not expressed at the mRNA

level, so it is important to confirm expression of the mutant allele at the mRNA level. To evaluate mRNA expression, complementary deoxyribonucleic acid-capture (cDNA) sequencing analyses can be performed. cDNA-capture sequencing can be used to successfully confirm expression of sequencing-identified mutant tumor-specific antigens at the mRNA level. This analysis also provides an estimation of how highly expressed the mutated allele is expressed relative to other genes in the tumor. For the clinical study proposed, tumor/normal exome sequencing analysis will be used to identify mutations (single nucleotide variants, fusions, insertions and deletions) present only in the tumor, and cDNA capture sequencing will be used to confirm mutant allele expression and expression level in the tumor mRNA.

3.2.2.3 Prioritization of Sequencing-Identified Neoantigens

[redacted]

The neoantigen DNA vaccine strategy is based on the DNA vaccine platform. The observation that direct administration of recombinant DNA can generate potent immune responses established the field of DNA vaccines in the early 1990s (10). Since that time, DNA vaccines have remained an area of intense research interest, and vaccines targeting infectious disease agents and cancers have progressed into clinical trials, including notably a DNA plasmid encoded tumor multi-antigen vaccine INO-5401, in combination with plasmid encoded IL-12 (interleukin 12; INO-9012) and anti-PD1 therapy (REGN2810; Cemiplimab) in subjects with newly diagnosed glioblastoma [NCT03491683; IND# 17671]. The INO-5401 Phase I/II study is based on the identical plasmid DNA backbone as is proposed in this study and uses the identical plasmid encoded IL-12 (INO-9012) as an adjuvant. Advantages of the DNA vaccine platform include the remarkable safety profile of DNA vaccines and the relative ease of manufacture, relative to proteins and other biologics. Perhaps most important, however, is the molecular flexibility of the DNA vaccine platform, with the ability to genetically manipulate encoded antigens, and/or incorporate other genes to amplify the immune response (11, 12). The molecular flexibility of the DNA vaccine platform allows us to target multiple neoantigens using a single polyepitope DNA vaccine. Polyepitope DNA vaccines integrate multiple epitopes in a single construct. We have optimized the polyepitope DNA vaccine to maximize antigen presentation of neoantigens (13) and the induction of antigen specific T-cells using plasmid encoded IL-12 as a molecular adjuvant [redacted].

3.2.2.4 Use of IL-12 Plasmid with DNA Vaccines

Preclinical studies showed that the immunogenicity of DNA vaccines can be substantially increased by the use of cytokine adjuvants (14-16). Co-administration of cytokine plasmids with DNA vaccines has been studied in rodents (15-17) and provided the basis for evaluation of INO-5401 + INO-9012 in humans. IL-12 is a heterodimeric cytokine, encoded by 2 separate genes, IL-12A (p35) and IL-12B (p40). Although p35

IL-12 gene transcripts are ubiquitous, p40 transcripts are unique to cells producing biologically active IL-12, which include monocytes, macrophages, dendritic cells, polymorphonuclear leukocytes, and B cells(18). These studies demonstrated a dramatic increase in specific Cytotoxic T Lymphocyte activity when a DNA plasmid was co-administered with an IL-12 plasmid, as compared with results in animals receiving therapeutic plasmid alone. The molecular adjuvant activity of several T-helper 1 cytokines (granulocyte-macrophage colony-stimulating factor, IL-2, IL-12, IL-15, and IL-18) was evaluated in mice in a subsequent study(17). This study revealed that the IL-12 plasmid was the best driver of MHC-restricted CD8+ Cytotoxic T Lymphocyte activity. Co-delivery of IL-12 DNA with DNA vaccines was also evaluated in macaques and chimpanzees with enhanced responses to DNA immunogens with IL-12 plasmid injections (19-21). A substantial enhancement of cellular immune responses with IL-12 DNA and DNA therapeutic plasmid administration compared with a DNA therapeutic plasmid alone in macaques has also been demonstrated(21, 22). Of note, no significant additional toxicity has been observed when cytokine adjuvants such as plasmid encoded IL-12 were co-administered with DNA vaccines in preclinical studies.

In a clinical trial with a vaccine of HIV-1 DNA immunogen, co-administration of IL-12 DNA was associated with an enhanced CD8+ antigen-specific immune response(23, 24). The use of IL-12 DNA has also been associated with expansion of antigen-specific IFN-γ positive effector cells, as well as granzyme B production. The induced immunity included both a CD8+ as well a CD4+ component(20).

3.2.2.5 Use of checkpoint inhibitors in combination with DNA Vaccines

Cancer vaccines that expand a functional T-cell response have the potential to synergize with checkpoint blockade immunotherapy. The ability of PD1 pathway blockade to synergize with a murine telomerase reverse transcriptase (TERT) DNA vaccine was demonstrated in a syngeneic TC-1 tumor model (13). The murine TERT DNA vaccine administered alone exhibited a robust capacity to generate an immune response against vaccine matched peptides and could break tolerance to native mouse TERT peptides in C57BL/6 mice. Therapeutic dosing with anti-PD1 alone or therapeutic vaccination with the murine TERT DNA vaccine alone slightly slowed tumor growth. However, anti-PD1 in combination with murine TERT DNA vaccine demonstrated significant slowing of tumor growth compared to non-treated mice or to mice receiving only the vaccine. Thus, these studies support the rationale to combine tumor antigen vaccines with PD1 pathway blockade in the clinic.

3.2.2.6 Use of Electroporation with DNA Vaccines

Several groups are developing methods to improve the immune responses of DNA vaccines, using genetic optimization, cytokine adjuvants, and alternative cellular delivery with devices such as electroporation (EP)(25). Geneos employs all these methods with an emphasis on improving the transfection of DNA using *in vivo* EP. This physical process exposes the target tissue to a brief electric field pulse that induces temporary and reversible pores in the cell membrane to enhance the cellular uptake of large molecules such as DNA. By temporarily increasing the permeability of cell membranes, EP has been shown to be an efficient way to introduce DNA into cells(26) and to increase the expression level of antigens encoded by DNA(27, 28). This technology has been used for more than 3 decades by molecular biologists for *in vitro* cell transfection including use of gene editing approaches for development of personalized therapies such Chimeric Antigen Receptor T-cell (CAR-T) therapy. The proposed device to be used in this study, CELLECTRA® 2000 device has been licensed from Inovio Pharmaceuticals for the purpose of enabling the uptake of the plasmid DNA encoding the personalized neoantigen vaccines.

The hypothesis is that EP will increase the uptake and expression of plasmid DNA, generating significantly increased immunogenicity. Electroporation enhances both cellular and humoral immune responses, using less DNA than intramuscular (IM) or ID immunizations alone(29). Studies have demonstrated the ability of EP to augment specific cellular immune responses in mice(30) and in macaques(31). It has been found

that Simian Immunodeficiency Virus (SIV) DNA vaccines potency can be increased 50- to 200-fold when delivered IM followed by EP. There are various means of delivering EP (i.e., constant current vs constant voltage) and testing these strategies in mice and pigs has shown a constant current device may be the most effective at generating immune responses(27). Studies in macaques found that EP of SIV DNA + IL-12 plasmids yielded 10-fold higher responses than DNA without EP(27), and that this immune response was boosted with additional doses. These responses are also polyfunctional, as defined by the ability of immune cells from treated animals to generate IFN-γ, TNF-α, and IL-2. Furthermore, the functional consequence of EP delivery of DNA encoding an antigen in combination with IL-12 is an improved ability of specific CD8+ T-cells to proliferate in culture in response to antigenic stimulation compared to delivery without EP (27). Additionally, it has been shown that delivering DNA vaccines with EP has a significant dose-sparing effect, along with superior immunogenicity when compared to DNA vaccines delivered without the use of EP (23, 24). Figure 2 depicts how EP works in the body.

Figure 2. How Electroporation Works in the Body

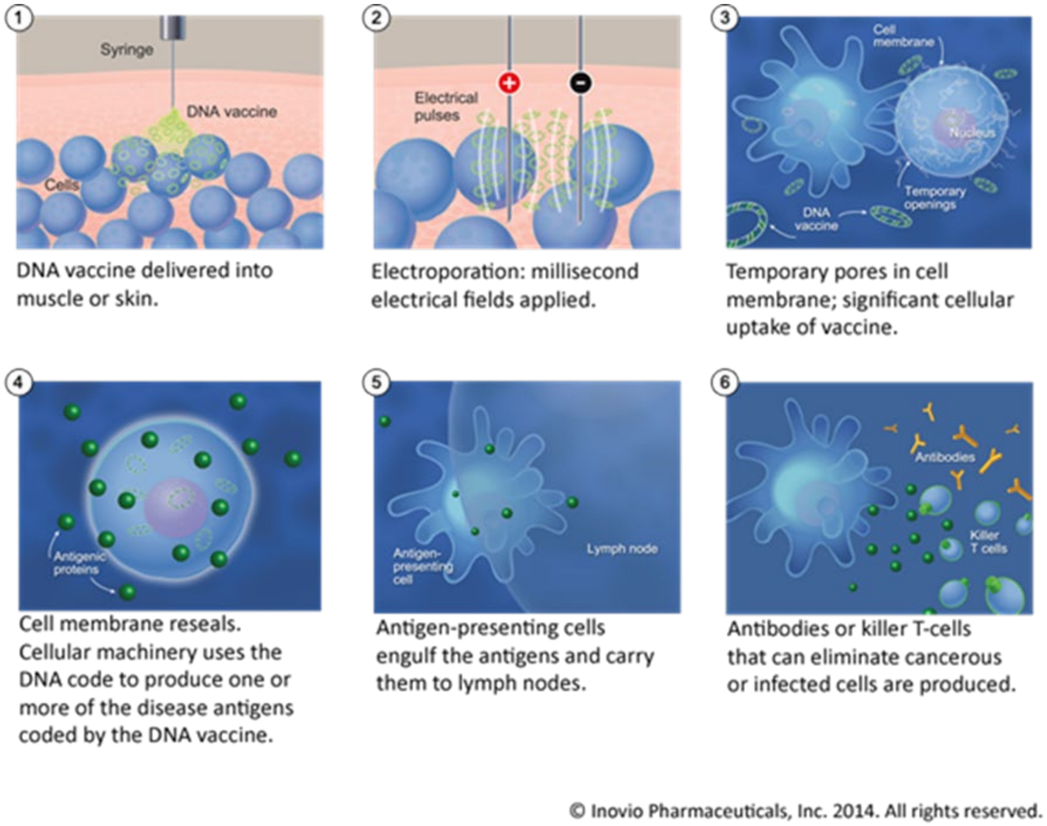

Following successful proof-of-concept studies in animals, we have optimized both pulse pattern and voltage to increase transfection efficiency. More recently, clinical applications of DNA or drug delivery via EP have been tested in the treatment of cancer and in gene therapy(32-34). To date, however, EP remains experimental in humans; it has not been licensed by the FDA for clinical use.

3.2.2.7 Toxicology and Biodistribution

[redacted]

[redacted]

[redacted]

[redacted]

[redacted]

[redacted]

[redacted]

3.2.2.8 Summary of Nonclinical Studies

[redacted]

3.2.3 Intradermal delivery of GNOS-PV02 + INO-9012 using CELLECTRA® 2000 device

[redacted]

3.3. Rationale for Dose Selection/Regimen

The pembrolizumab dose of 200 mg dose IV infusion over 30 minutes Q3W has been chosen based on the approved dose for pembrolizumab in this indication.

The GNOS-PV02 dose of 1 mg and INO-9012 dose of 0.34 mg was chosen because in previous clinical studies of DNA vaccine intradermal injection and electroporation(46, 47) antigen vaccine plasmid + IL12 administration at 0.8-1.6 mg of DNA antigen + 0.2-0.4 mg of IL12 plasmid was safe and generated strong immune responses following 3 dose regimens.

3.4. Benefit/Risk

Any direct benefit for subjects from participating in this clinical study is unknown. The median OS of subjects in the second-line metastatic setting, who progress or are intolerant of TKIs, is less than 12 months. Therefore, this population represents a high unmet medical need, and overall, the clinical benefit potential outweighs the risks associated with GNOS-PV02 + INO-9012. The known risks of GNOS-PV02 + INO-9012 are described in the pre-clinical and clinical background sections in the GNOS-PV02 + INO 9012 IB.

The known risks of pembrolizumab are described in the package insert.

4. PHARMACEUTICAL AND THERAPEUTIC BACKGROUND

4.1. Neoantigen DNA Vaccine, GNOS-PV02

4.1.1 Chemical Name and Structure

The personalized neoantigen DNA vaccines are also known as DNA plasmid vectors expressing tumor specific antigens.

Tumor tissue sequencing is performed under Clinical Laboratory Improvement Program/Clinical Laboratory Improvement Amendment (CLIP/CLIA) conditions. Neoantigen identification and prioritization is performed by Geneos Therapeutics. The neoantigen DNA vaccine is designed by Geneos Therapeutics and manufactured based on the following general steps:

1. Cancer tissue and normal lymphocytes are obtained from subjects who are eligible for the phase I clinical study.
2. Tumor/normal exome sequencing and tumor RNA sequencing are performed to identify candidate neoantigens.
3. Candidate neoantigens are prioritized based on epitope prediction algorithms.
4. Personalized polyepitope inserts integrating the prioritized (up to 40) neoantigens (up to 33 amino acids in each neoantigen) are designed and then synthesized and cloned into the pGX0001 parent vector.
5. The parent vector is transformed into E. coli, and the neoantigen DNA plasmid vaccine is manufactured and vialed.
6. The neoantigen DNA vaccine, GNOS-PV02, undergoes product release tests prior to investigational use.
7. GNOS-PV02 is administered in combination with INO-9012 (plasmid IL-12) using a pharmacy protocol for on-site mixing of the 2 drug products. The 2 plasmid DNA products are combined and administered using the CELLECTRA® 2000 device.

Each neoantigen DNA vaccine drug product is composed of a DNA plasmid purified from E. coli. [redacted]

[redacted]

[redacted]

[redacted]

[redacted]

[redacted]

[redacted]

4.1.2 *Manufacturing facility*

The neoantigen DNA vaccine (GNOS-PV02) is manufactured at [redacted]. The facility is located at [redacted]

The facility adheres to current Good Manufacturing practices cGMP with regard to documentation, facility maintenance, and quality control/quality assurance review. Segregated manufacturing areas are available for clinical grade manufacturing of recombinant DNA plasmid products.

4.1.3 Manufacturing process

[redacted]

[redacted]

[redacted]

[redacted]

[redacted]

[redacted]

[redacted]

[redacted]

[redacted]

[redacted]

[redacted]

[redacted]

[redacted]

[redacted]

[redacted]

[redacted]

5. OBJECTIVES AND ENDPOINTS

**Objectives and Endpoints**	
**Primary Objectives:**	**Associated Primary Endpoints:**
1. To determine the safety and tolerability of GNOS-PV02 + INO-9012 delivered by ID injection followed by EP using the CELLECTRA® 2000 device in combination with pembrolizumab	• Adverse events as graded by CTCAE v5.0
2. To evaluate preliminary immune response to GNOS-PV02 +INO-9012 in combination with pembrolizumab in subjects with previously treated advanced HCC	Neoantigen-specific cellular immune responses that may be assessed by but not limited to:<br>• Interferon-γ secreting T lymphocytes in PBMCs by ELI Spot<br>• T-cell activation and cytolytic cell phenotype in PBMCs by flow cytometry or secretion of immune molecules
**Secondary Objective**	**Associated Secondary Endpoints**
To evaluate the anti-tumor activity of GNOS-PV02 + INO-9012 in combination with pembrolizumab in subjects with previously treated advanced HCC	• ORR by RECIST 1.1 by investigator review<br>• ORR by iRECIST<br>• DOR<br>• DCR<br>• PFS as assessed by RECIST 1.1 and iRECIST<br>• OS
**Exploratory Objective**	**Associated Exploratory Endpoints**
1. To determine the feasibility of generating the personalized neoantigen DNA vaccine (GNOS-PV02) for subjects with advanced HCC	The feasibility assessment will be based on:<br>• Tumor-specific neoantigen identification<br>• Successful neoantigen-based DNA vaccine manufacturing

Objectives and Endpoints	
	• The time period elapsed between tumor tissue collection and availability of the neoantigen vaccine for subject dosing
2. To evaluate tumor and immune biomarkers and their association with treatment outcome (antitumor activity and/or safety) in subjects with previously treated advanced hepatocellular carcinoma	• Assessment of MDSC<br>• TCR sequencing of PBMCs for diversity and putative antigen specificity<br>• Immune gene transcript profiling of PBMCs<br>• Assessment of pro-inflammatory and immunosuppressive elements in neoplastic and adjacent normal tissue, where feasible<br>• Pre- and post-treatment peripheral blood and tumor immune related gene expression -specific oncoproteins in tissue<br>• MicroRNA profiling to predict treatment efficacy evaluating pre- and post-treatment peripheral blood samples<br>• ctDNA analysis and tracking for progression

CTCAE = Common Terminology Criteria for Adverse Events; DCR = disease control rate; DOR = duration of response; HCC = hepatocellular carcinoma; MDSC = myeloid derived suppressor cells; ORR = overall response rate; OS = overall survival; PBMC = peripheral blood mononuclear cell; PFS = progression free survival; TCR = T-cell receptor

6. STUDY DESIGN

6.1. Research Hypotheses

GNOS-PV02 + INO-9012 delivered by ID injection followed by EP using the CELLECTRA® 2000 device in combination with pembrolizumab, will be generally safe, well tolerated, immunogenic, and lead to anti-tumor responses in adult subjects with previously treated advanced HCC.

6.2. Overall Design

This is a single-arm, open-label, multi-site Phase I/IIa study of a personalized neoantigen DNA vaccine (GNOS-PV02) and plasmid encoded IL-12 (INO-9012) delivered ID using the CELLECTRA® 2000 device in combination with pembrolizumab (MK-3475) in subjects with histologically or cytologically confirmed diagnosis of HCC based on pathology report. To be eligible, subjects must be receiving or scheduled to begin first-line therapy with sorafenib or lenvatinib and must provide a tissue sample for personalized neoantigen DNA vaccine development. Subjects will begin therapy with the personalized neoantigen DNA vaccine in combination with pembrolizumab upon discontinuation of first-line therapy at the discretion of the patient's primary oncologist (i.e., objective radiographic progression on sorafenib, lenvatinib, or else intolerance to sorafenib or lenvatinib). Additionally, subjects must also have disease not amenable to a curative treatment approach (e.g., transplant, surgery, or ablation). Subjects must also have at least 1 measurable lesion per RECIST 1.1, Child-Pugh liver score A, an ECOG performance score of 0 or 1 and predicted life expectancy of greater than 6 months. An imaging scan is required at Day 0 up to 21 days prior to pembrolizumab initiation. Subjects are required to provide a tumor tissue sample for whole exome

sequencing and personalized neoantigen vaccine development. Approximately 36 subjects will receive combination therapy with the personalized neoantigen DNA vaccine (1 mg) + INO-9012 (0.34 mg) given ID via CELLECTRA® 2000 EP and pembrolizumab 200 mg IV. The total DNA vaccine dose is split between 2 doses delivered 1 after another and administered by an ID injection via CELLECTRA® 2000 EP into 2 locations (i.e., both arms; see section 8.1.2.1 and pharmacy manual).

A key objective of this study is to determine the ORR of the personalized neoantigen DNA vaccine given in combination with pembrolizumab. Beginning with screening, all imaging assessments will be evaluated using RECIST 1.1. On-study imaging assessments will be performed (every 9 weeks) Q9W, calculated from the date of therapy initiation and independent of treatment delays. RECIST 1.1 will be used by the site for treatment decisions until the first radiologic evidence of PD.

Following the first radiologic evidence of PD by RECIST 1.1, treatment decisions may be made by using Immune Response Evaluation Criteria in Solid Tumors (iRECIST) to accommodate tumor response patterns seen with checkpoint inhibitor therapy including pembrolizumab treatment (e.g., tumor flare). This was first described by Nishino, et al. 2013(48) and is used in immunotherapy clinical studies. For a clinically stable subject with first radiologic evidence of PD, it is at the discretion of the site investigator to continue treating the subject with the personalized vaccine and pembrolizumab until PD is confirmed at least 4 weeks after the date of the first tumor imaging suggesting PD, per the site investigator. If radiologic PD is confirmed by the subsequent tumor imaging, the subject should be discontinued from treatment unless, in the opinion of the investigator, the subject is achieving a clinically meaningful benefit. In this case, an exception for continued treatment may be considered following consultation with the Sponsor.

Subjects may continue to be treated with the personalized neoantigen vaccine and pembrolizumab until PD is confirmed by RECIST 1.1 with a second imaging at least 4 weeks apart, unacceptable AEs, intercurrent illness that prevents further administration of treatment, investigator's decision to withdraw the subject, subject withdrawal of consent, pregnancy of the subject, noncompliance with study drug or procedure requirements, administrative reasons, or the subject has received approximately 2 years therapy with GNOS-PV02 and pembrolizumab. Subjects who discontinue study drug for a reason other than disease progression will move into the follow-up phase and should be assessed Q9W (63±7 days) by radiologic imaging to monitor disease status. Disease status will continue to be monitored until whichever of the following occurs first: the start of new anti-cancer treatment, disease progression, death, or the end of the study. All subjects will be followed every 12 weeks for OS until death, lost to follow-up, withdrawal of consent, or the end of the study.

Subjects who attain a complete response (CR) by 2 tumor imaging assessments at least 4- 6 weeks apart and who have received approximately 6 months of therapy with the personalized neoantigen vaccine and pembrolizumab may discontinue treatment at the discretion of the investigator after receiving at least 2 treatments beyond the initial determination of a CR. Subjects who stop GNOS-PV02 and/or pembrolizumab after receiving approximately 2 years of therapy for reasons other than PD or intolerability, or who stop after attaining a CR, may be eligible for retreatment with up to an additional 1 year of therapy (second course of treatment) after they have experienced radiographic PD. The decision to retreat will be at the discretion of the investigator, only if no other cancer treatment was administered since the last dose of GNOS-PV02 and pembrolizumab, the subject still meets the parameters listed in the inclusion and exclusion criteria, and the study remains open.

AEs will be monitored throughout the study and graded in severity according to the guidelines outlined in the Common Terminology Criteria for Adverse Events (CTCAE) version 5.0. After the end of treatment, each subject will be followed for 30 days for AE monitoring. SAEs will be collected until the patient reaches the end of the study.

In addition to regularly scheduled blood sample collections, tumor tissue samples will be collected whenever clinically feasible and available: pre-treatment, post-4 doses of vaccine (Week 9, Cycle 4, on-treatment) and at time of disease progression. Also, whenever treatment-naïve archival tissue is available, it will be requested. Pre-treatment tissue sample is required and will be prioritized for tumor DNA whole exome and tumor RNA sequencing (for vaccine design & manufacturing). Additionally, the on-treatment tissue sample collection (Week 9) is strongly recommended.

It is anticipated that the personalized DNA vaccine will have been manufactured and available for delivery in combination with pembrolizumab at the time of progression or intolerance to sorafenib or lenvatinib. If patients discontinue first-line therapy and the personalized DNA vaccine is not yet available, participants may wait for the vaccine to be manufactured, or come off study to receive standard of care second-line therapy. For the first dose of GNOS-PV02, pembrolizumab will be administered first followed by dosing of GNOS-PV02. For subsequent doses, pembrolizumab and GNOS-PV02 can be given in any particular order.

Subjects enrolled for whom vaccine manufacturing was not successful or fail to meet eligibility criteria upon progression or intolerance with sorafenib or lenvatinib will be replaced to keep the total number of evaluable subjects of approximately 36.

Subjects who have progressed with evidence of growth of existing target, non-target or development of new lesions while on GNOS-PV02 treatment may have another personalized vaccine manufactured and administered using a new tumor biopsy sample per investigator's discretion and Sponsor's approval. Once the subject receives their personalized vaccine, they will continue on study treatment until 2 years (W99) from the Day 0 visit.

Dose-limiting toxicities (DLTs) are defined in the protocol Section 10.3.8

Because this study is treating subjects with GNOS-PV02 and pembrolizumab for the first time, there will be a waiting period of 1 week between dosing of the first subject and the second subject.

Stopping rules for AEs will be employed for this study (Section 10.3.9).

To ensure subject safety during the study, a Safety Committee will be formed to monitor safety on a periodic basis. Members of the Safety Committee will include a Safety Expert, Principal Investigator(s) and the Sponsor Medical Monitor. The Safety Committee will meet approximately every 6 months to review safety data collected from the point of first patient in to review safety, or as needed. The safety data will include demographic data, AEs, SAEs, and relevant laboratory data. Following each data review, the Safety Committee will provide recommendations as to whether the study should continue, be amended, or whether the study should be stopped on the basis of safety (i.e., evidence of harm). The final decision will rest with the Sponsor. Any outcomes of these safety reviews that affect study conduct will be communicated in a timely manner to the investigators for notification of the institutional review boards/independent ethics committees (IRBs/IECs).

6.3. End of Study Definition

The clinical study will be considered completed when all subjects have had their 3-year follow-up visit, death, lost to follow-up, withdrawal of consent, or when the Sponsor deems the study completed, whichever comes first.

7. STUDY POPULATION

Male and female subjects with advanced HCC currently receiving or scheduled to receive first-line treatment with sorafenib or lenvatinib with no curative option will be enrolled in this study. Combination therapy with GNOS-PV02, INO-9012 and pembrolizumab will begin after discontinuation of first-line therapy at the discretion of the patient's primary oncologist.

7.1. Inclusion Criteria

In order to be eligible for participation in this study, the subject must:

1. Be willing and able to provide written informed consent for the study. The subject may also provide consent for future biomedical research (FBR). However, the subject may participate in the main study without participating in FBR.
2. 18 years of age on day of signing informed consent.
3. Have histologically or cytologically confirmed diagnosis of HCC (not accepted: fibrolamellar, sarcomatoid, mixed cholangiocarcinoma). Radiological diagnosis is valid to initiate screening pending confirmation by pathology.
4. Have BCLC Stage C disease or BCLC Stage B disease not amenable to locoregional therapy, or refractory to locoregional therapy, and not amenable to a curative treatment approach.
5. Have a Child-Pugh Class A liver score.
6. Have a predicted life expectancy of greater than 6 months.
7. Have measurable disease based on RECIST 1.1.
8. Have a performance status of 0 or 1 using the ECOG Performance Scale within 7 days of first dose of study drug.
9. Receiving or eligible for first-line therapy with sorafenib or lenvatinib.
10. Willing to submit a tissue sample for personalized DNA vaccine manufacturing.
11. Patients with chronic or acute HBV infection [as characterized by positive hepatitis B surface antigen (HBsAg) and/or hepatitis B core antibodies (anti-HBcAb) with detectable HBV DNA (≥10 IU/ml)] must be treated with effective antiviral therapy, as per institutional practices, prior to enrollment and for the duration of the study therapy. Patients who test positive for anti-hepatitis B core (HBc) with undetectable HBV DNA (<10 IU/ml) do not require anti-viral therapy prior to enrollment however these subjects will be tested at every cycle to monitor HBV DNA levels and initiate antiviral therapy if HBV DNA is detected (≥10 IU/ml).Subjects with chronic infection by hepatitis C virus (HCV), who are untreated, are allowed on study. In addition, subjects with successful HCV treatment (defined as sustained virologic response 12 or SVR 24) are allowed, as long as 4 weeks have passed between completion of HCV therapy and start of study drug. Subjects receiving antiviral therapy during TKI may be enrolled.
12. Women of childbearing potential must have a negative urine or serum pregnancy test for the patient to be eligible for trial enrolment.
13. Administration of GNOS-PV02 and pembrolizumab may have an adverse effect on pregnancy and poses a risk to the human fetus, including embryo-lethality. Women of childbearing potential must agree to use either two adequate barrier methods or a barrier

method plus a hormonal method of contraception to prevent pregnancy, or to abstain from heterosexual activity (complete abstinence) prior to study entry, for the duration of study participation, and for 5 months (150 days) after the last dose of study agent. Should a woman become pregnant or suspect she is pregnant while she or her partner is participating in this study, she should inform her treating physician immediately. Male patients must agree to use an adequate method of contraception, or to abstain from heterosexual activity (complete abstinence), prior to study entry, for the duration of study participation, and for 5 months (150 days) after the last dose of study agent.

14. Demonstrate adequate organ function as defined in Table 6.

Table 6. Adequate Organ Function Laboratory Values

System	Laboratory Value
**Hematological**	
Absolute neutrophil count	≥1200/µL
Platelets	≥100,000/µL
Hemoglobin	≥8g/dL without transfusion or EPO dependency within 7 days.
**Renal**	
Creatinine **OR**<br>Measured or calculated creatinine clearance<br>(GFR can also be used in place of creatinine or creatinine clearance)	≤1.5×ULN **OR**<br>≥60 mL/min for subject with creatinine levels >1.5× institutional ULN<br>*Note: Creatinine clearance should be calculated per institutional standard*
**Hepatic**	
Total bilirubin	≤2 mg/dL, or direct bilirubin ≤ULN for those with total bilirubin >2 mg/dL
AST/SGOT and alanine ALT/SGPT	≤5×ULN
Albumin	≥3.0 g/dL
**Coagulation**	
International normalized ratio (INR) or prothrombin time/aPTT	≤1.5×ULN unless subject is receiving anticoagulant therapy as long as prothrombin time or aPTT is within therapeutic range of intended use of anticoagulants

ALT = alanine aminotransferase; aPTT = activated partial thromboplastin time; AST = aspartate aminotransferase; EPO = erythropoietin; GFR = glomerular filtration rate; SGOT = serum glutamic-oxaloacetic transaminase; SGPT = serum glutamic-pyruvic transaminase; ULN = upper limit of normal

7.2. Exclusion Criteria

Subjects who meet any of the following criteria will be excluded from study entry:

1. Is currently participating and receiving study drug or has participated in a study of an investigational agent and received study drug or used an investigation device, within 4 weeks of the first dose of treatment. Subjects must also have recovered from associated therapy (i.e., to Grade ≤1 or baseline) and from AEs due to any prior therapy.
2. Has received sorafenib or lenvatinib within 14 days of first dose of study drug.
3. Has had esophageal or gastric variceal bleeding within the last 6 months. If suspected, subjects will be screened for esophageal varices. If varices are present, they should be treated according to institutional standards before starting study treatment.
4. Has clinically apparent ascites on physical examination. Note: only ascites detectable on imaging studies is allowed.
5. Evidence of portal vein invasion based on imaging is allowed pending subjects meet laboratory criteria for enrollment.
6. Has had encephalopathy in the last 6 months. Subjects receiving rifaximin or lactulose to control their encephalopathy are not allowed.
7. Had a solid organ or hematologic transplant.
8. Had prior systemic therapy for HCC other than sorafenib or lenvatinib.
9. Has active autoimmune disease that has required systemic treatment in past 2 years (Appendix A) (i.e., with use of disease-modifying agents, corticosteroids, or immunosuppressive drugs). Replacement therapy (e.g., thyroxine (T4), insulin, or physiologic corticosteroid replacement therapy for adrenal or pituitary insufficiency, etc.) is not considered a form of systemic treatment.
10. Has a diagnosis of immunodeficiency or is receiving systemic steroid therapy or any other form of immunosuppressive therapy within 7 days prior to the first dose of study drug. The use of physiologic doses of corticosteroids may be approved after consultation with the Sponsor.
11. Has received locoregional therapy to liver TACE, transarterial embolization, radiation, radioembolization, or ablation) or major surgery to liver or other site within 3 weeks prior to the first dose of study drug. Minor surgery (e.g., simple excision, tooth extraction) must have occurred at least 7 days prior to the first dose of study drug (Cycle 1, Day 1). Subjects must have recovered adequately (i.e., Grade ≤1 or baseline) from the toxicity and/or complications from any intervention prior to starting therapy.
12. Has a diagnosed additional malignancy within 5 years prior to first dose of study drug, with the exception of: (a) non-invasive carcinomas subject to successful curative treatment in the opinion of the investigator which require no further therapy and (b) other malignancies for which patients have undergone potentially curative therapy and have been considered disease free for at least 3 years prior to screening.
13. Has radiographically detectable (even if asymptomatic and/or previously treated) central nervous system (CNS) metastases and/or carcinomatous meningitis, as assessed by local site investigator.
14. Has a known history of, or any evidence of, interstitial lung disease or active non-infectious pneumonitis.

15. Has an active infection requiring systemic therapy.
16. Has a history or current evidence of any condition, therapy, or laboratory abnormality that might confound the results of the study, interfere with the subject's participation for the full duration of the study, or is not in the best interest of the subject to participate, in the opinion of the treating investigator, including but not limited to active infection requiring systemic therapy, symptomatic congestive heart failure, unstable angina pectoris, cardiac arrhythmia, or dialysis.
17. Has known psychiatric or substance abuse disorders that would interfere with cooperation with the requirements of the study.
18. Is pregnant or breastfeeding or expecting to conceive or father children within the projected duration of the study, starting with the screening visit through 150 days after the last dose of study drug.
19. Has received prior immunotherapy including anti–PD-1, anti–PD-L1, or anti–PD-L2 agents, or personalized therapies such as adoptive cell therapy or neoantigen-based vaccine. Note: oncolytic viruses are not considered immunotherapy.
20. Has a known history of HIV (HIV I/II antibodies).
21. Has untreated active HBV, unless planned antiviral therapy during TKI.

    Note: Patients with HBV infection, characterized by positive HBsAg and/or HBcAb with detectable HBV DNA (≥10 IU/ml or above the limit of detection per local lab standard), must be treated with antiviral therapy as per institutional practice to ensure adequate viral suppression (HBV DNA ≤2000 IU/mL) prior to treatment with the study drug. Patients who test positive for HBcAg with undetectable HBV DNA (<10 IU/ml or under the limit of detection per local lab standard) do not require anti-viral therapy prior to enrollment.
22. Has received a live vaccine within 30 days of planned start of study treatment (Cycle 1, Day 1).

    Note: The killed virus vaccines used for seasonal influenza vaccines for injection are allowed; however intranasal influenza vaccines (e.g., FluMist®) are live attenuated vaccines and are not allowed. Non-live COVID-19 vaccines are allowed.
23. Any contraindication for treatment with the CELLECTRA® 2000 Device:

    Less than two acceptable sites available for ID injection and EP considering the deltoid and anterolateral quadriceps muscles:

    - Tattoos, keloids or hypertrophic scars located within 2 cm of intended administration site;
    - Implantable-Cardioverter-defibrillator (ICD) or pacemaker (to prevent a life-threatening arrhythmia) that is located ipsilateral to the deltoid injection site (unless deemed acceptable by a cardiologist);
    - Any metal implants or implantable medical device within the intended treatment site (i.e. electroporation area).
24. Has no mutations detected after sequencing of the tumor.

7.3. Abbreviated Inclusion and Exclusion criteria (After Completion of Standard of Care TKI Therapy, Prior to Investigational Therapy)

After discontinuing first-line therapy with lenvatinib or sorafenib, participants must be screened using the abbreviated inclusion and exclusion criteria described here. There is a minimum of a 14-day washout from first-line therapy with lenvatinib or sorafenib until patients may initiate study therapy. Participants must meet these criteria in order to receive treatment with GNOS-PV02 and pembrolizumab on protocol. Participants who met initial screening criteria but fail to meet the subsequent abbreviated screening criteria are considered screen failures and should receive standard of care therapy at the discretion of their treating oncologist.

7.3.1 Inclusion

In order to be eligible for participation in this study, following discontinuation of first-line therapy, the subject must:

1. Have measurable disease based on RECIST 1.1.
2. Have a Child-Pugh class A liver score.
3. Have a predicted life expectancy of greater than 3 months.
4. Have a performance status of 0 or 1 using the ECOG Performance Scale.
5. Demonstrate organ function as defined in Table 7.

Table 7. Adequate Organ Function Laboratory Values

System	Laboratory Value
**Hematological**	
Absolute neutrophil count	≥1200/µL
Platelets	≥75,000/µL
Hemoglobin	≥8g/dL without transfusion or EPO dependency within 7 days.
**Renal**	
Creatinine **<u>OR</u>**<br>Measured or calculated creatinine clearance<br>(GFR can also be used in place of creatinine or creatinine clearance)	≤1.5×ULN **<u>OR</u>**<br>≥60 mL/min for subject with creatinine levels >1.5× institutional ULN<br>*Note: Creatinine clearance should be calculated per institutional standard*
**Hepatic**	
Total bilirubin	≤2.5×ULN , or direct bilirubin ≤ULN for those with total bilirubin >2 mg/dL
AST/SGOT and alanine ALT/SGPT	≤5×ULN
Albumin	≥2.8 g/dL
**Coagulation**	

System	Laboratory Value
INR or prothrombin time/aPTT	≤1.5×ULN unless subject is receiving anticoagulant therapy as long as prothrombin time or aPTT is within therapeutic range of intended use of anticoagulants

ALT = alanine aminotransferase; aPTT = activated partial thromboplastin time; AST = aspartate aminotransferase; EPO = erythropoietin; GFR = glomerular filtration rate; SGOT = serum glutamic-oxaloacetic transaminase; SGPT = serum glutamic-pyruvic transaminase; ULN = upper limit of normal;

7.3.2 Exclusion

Subjects who meet any of the following criteria after discontinuation of first-line therapy will be excluded from study entry:

1. Has received locoregional therapy to liver TACE, transarterial embolization, radiation, radioembolization, or ablation) or major surgery to liver or other site within 3 weeks prior to the first dose of study drug. Minor surgery (e.g., simple excision, tooth extraction) must have occurred at least 7 days prior to the first dose of study drug (Cycle 1, Day 1). Subjects must have recovered adequately (i.e., Grade ≤1 or baseline) from the toxicity and/or complications from any intervention prior to starting therapy.
2. Has a diagnosis of immunodeficiency or is receiving systemic steroid therapy or any other form of immunosuppressive therapy within 7 days prior to the first dose of study treatment. The use of physiologic doses of corticosteroids may be approved after consultation with the Sponsor.
3. Has received any systemic therapy for hepatocellular carcinoma within 14 days of study treatment.
4. Prior treatment with any anti-PD-1 or anti-PD-L1 antibody.
5. Has a history or current evidence of any condition, therapy, or laboratory abnormality that might confound the results of the study, interfere with the subject's participation for the full duration of the study, or is not in the best interest of the subject to participate, in the opinion of the treating investigator, including but not limited to active infection requiring systemic therapy, symptomatic congestive heart failure, unstable angina pectoris, cardiac arrhythmia, or dialysis.
6. Is pregnant or breastfeeding or expecting to conceive or father children within the projected duration of the study, starting with the screening visit through 150 days after the last dose of study drug.

7.4. Screen Failures

Subjects will be considered screen failures if the personalized neoantigen DNA vaccine is not able to be designed, manufactured, or pass quality qualifications for administration.

If a subject fails to meet an eligibility criterion during the screening or manufacturing period, then the Investigator will complete the screen failure section in the electronic case report form (eCRF).

Subjects who have signed informed consent, but do not complete screening, and who do not receive treatment on study with GNOS-PV02 + INO-9012 may be replaced for efficacy, toxicity, and translational analyses.

7.5. Strategies for Recruitment and Retention

It is anticipated that up to 4 sites will participate in the study. Patients will be recruited from either standalone outpatient clinics or hospital clinics.

8. STUDY INTERVENTION

8.1. Study Intervention(s) Administration

8.1.1 Study Treatment Description

8.1.1.1 GNOS-PV02 + INO-9012 Treatment

The GNOS-PV02 personalized neoantigen DNA vaccine, is a purified plasmid DNA preparation which is diluted into sterile 20X SSC buffer then adjusted, if necessary, with 1X SSC to the final concentration of 6 mg/mL in SSC buffer (150 mM saline, 15 mM sodium citrate, pH 7.0). The formulated DNA plasmid product undergoes redundant sterile 0.2 µm filtration. Using an automated filling machine, sterile-filtered plasmid product is aseptically added to pre-sterilized 2.0 mL vials and stored refrigerated.

Sufficient plasmid DNA is synthesized for the subject doses, product release testing, and other requirements.

8.1.1.2 Pembrolizumab Treatment

Pembrolizumab is an FDA-approved PD-1-blocking antibody that is indicated for the treatment of patients with different malignancies. It is a humanized monoclonal antibody that blocks the interaction between PD-1 and its ligands, PD-L1 and PD-L2. Pembrolizumab is an IgG4 kappa immunoglobulin with an approximate molecular weight of 149 kDa. Pembrolizumab is produced in recombinant Chinese hamster ovary cells.

KEYTRUDA© may be supplied in 2 different formulations:

- KEYTRUDA© for injection is a sterile, preservative-free, white to off-white lyophilized powder in single-dose vials. Each vial is reconstituted and diluted for intravenous infusion. Each 2 mL of reconstituted solution contains 50 mg of pembrolizumab and is formulated in L-histidine (3.1 mg), polysorbate 80 (0.4 mg), and sucrose (140 mg). May contain hydrochloric acid/sodium hydroxide to adjust pH to 5.5.
- KEYTRUDA© injection is a sterile, preservative-free, clear to slightly opalescent, colorless to slightly yellow solution that requires dilution for intravenous infusion. Each vial contains 100 mg of pembrolizumab in 4 mL of solution. Each 1 mL of solution contains 25 mg of pembrolizumab and is formulated in: L-histidine (1.55 mg), polysorbate 80 (0.2 mg), sucrose (70 mg), and Water for Injection, USP.

For the purpose of this protocol, study treatment is defined as GNOS-PV02 + INO-9012 and pembrolizumab.

8.1.2 Dosing, Administration and Preparation

The study treatment will be administered only to patients included in this study following the procedures set out in this clinical study protocol. Administration of the study treatment will be supervised by the Investigator or sub-Investigator. Details of the exact time of administration of medication (day/month/year, hour:minute) will also be documented in the eCRF.

8.1.2.1 GNOS-PV02 + INO-9012 Dosing

The GNOS-PV02 + INO-9012 vaccine will be delivered by ID injection followed by EP using the CELLECTRA® 2000 device. Subjects will receive one ID injection of GNOS-PV02 + INO-9012 above each of two acceptable muscle locations for injection, each in a volume of ~0.1 mL. The injection site is assessed for investigational product (IP) leakage and followed immediately by EP using the CELLECTRA® 2000 device.

Only if the deltoid muscle is not a suitable location (see exclusion criterion '23'), the ID injection can then be administered above the lateral quadriceps followed immediately by EP. The IP must not be given within 2 cm of a tattoo, scar, keloid or previous injection site in the presence of an active lesion/rash. Also, ID injection followed immediately by EP must not be performed in the muscle of an extremity which has any metal implants or implantable medical device (e.g., surgical rods, pins or joint replacements). If a subject has an implanted cardiac defibrillator or pacemaker, ID injection/EP must not be performed above the deltoid muscle on the same side of the body where the device is located.

Patients will be administered 1 mg GNOS-PV02 + 0.34 mg INO-9012 every dose. Patients will receive GNOS-PV02 + INO-9012 on a staged schedule starting Q3W for 4 doses and thereafter Q9W. Patients who continue to dose past 24 months, may receive GNOS-PV02 + INO-9012 Q12W.

GNOS-PV02 + INO-9012 will be dosed approximately 1 hour after IV infusion of pembrolizumab for the first dose. Subsequent doses of GNOS-PV02 + INO-9012 and pembrolizumab can be dosed in any order.

A 30-minute observation period is recommended after each study treatment.

Additional information on dosing, administration and preparation can be found in the pharmacy manual for GNOS-PV02 + INO-9012.

8.1.2.2 Pembrolizumab Dosing

A fixed dose of 200 mg pembrolizumab will be administered to the subject, as an IV infusion over 30 minutes Q3W. A sterile, non-pyrogenic, low-protein binding 0.2 micron to 5 micron in-line or add-on filter will be utilized. Do not co-administer other drugs through the same infusion line.

A 60-minute observation period is recommended for the first dose on this study and 30 minutes for subsequent doses.

Additional information of Dosing, Administration and Preparation can be found in the package insert for Keytruda© https://www.merck.com/product/usa/pi_circulars/k/keytruda/keytruda_pi.pdf.

8.1.2.3 Study Drug Administration

Subjects will receive their study drug as described in Section 8.1.2.1 and Section 8.1.2.2 until disease progression, unacceptable toxicity, deemed intolerable by investigator, or up to 36 months in subjects without disease progression.

There will be no dose reductions. See Section 8.5 for dose modifications.

8.2. Preparation/Handling/Storage/Accountability

8.2.1 Acquisition and Accountability

The GNOS-PV02 + INO-9012 drug product and the CELLECTRA® 2000 device, will be packaged by the Sponsor, and supplied in an open-label basis. The Investigator or designee is responsible for storing, administering, and accounting for the GNOS-PV02 + INO-9012 according to the instructions provided by the Sponsor. GNOS-PV02 + INO-9012 shall be dispensed in accordance with the investigator's prescription,

and it is the investigator's responsibility to ensure that an accurate record of GNOS-PV02 + INO-9012 received, issued, returned, and destruction is maintained.

GNOS-PV02 + INO-9012 should not be destroyed until the site has contacted the Sponsor to provide disposition details. The CELLECTRA® 2000 device should be returned to [redacted] The person responsible for drug dispensing is required to maintain adequate records of GNOS-PV02 + INO-9012. These records (e.g., product accountability log) include the date the GNOS-PV02 + INO-9012 is received from the Sponsor and administered to the subject.

After review by the Sponsor or designee, all used and unused GNOS-PV02 + INO-9012 should be discarded at the investigational site, in accordance with the site's institutional standard operating procedures and/or polices, and a destruction certificate obtained. The investigator or designee will submit the copy of Product Accountability Log to the Sponsor. If the investigational site is unable to destroy GNOS-PV02 + INO-9012, the Sponsor will provide alternate instructions for destruction.

Under no circumstances will the investigator supply GNOS-PV02 + INO-9012 to a third party, allow GNOS-PV02 + INO-9012 be used other than as directed by this Clinical Study Protocol, or dispose of GNOS-PV02 + INO-9012 in any other manner.

Any quality issue noticed with the receipt or use of GNOS-PV02 + INO-9012, as well as the CELLECTRA® 2000 device (deficiency in condition, appearance, pertaining documentation, labeling, expiry date, etc.) should be promptly notified [redacted] who will initiate a complaint procedure.

A potential defect in the quality of the GNOS-PV02 + INO-9012 vaccine may be subject to a recall procedure by the Sponsor. In this case, the investigator will be responsible for promptly addressing any request made by the Sponsor, to recall GNOS-PV02 + INO-9012 and eliminate potential hazards.

8.2.2 Formulation, Appearance, Packaging and Labeling for GNOS-PV02 + INO-9012

Refer to the IB for formulation, appearance, packaging and labeling and the GNOS-PV02 + INO-9012 Study Pharmacy Manual for a description of the process of preparation for study drug administration.

Pembrolizumab will be labeled as per the manufacturer's specification.

8.2.3 Product Storage and Stability

The individual vials of GNOS-PV02 are stored refrigerated between 2–8°C, while INO-9012 is kept frozen at <-15°C. At the time of administration, a single neoantigen DNA vaccine product vial, GNOS-PV02, is removed from refrigerated storage. In-use procedures for removal of INO-9012 from frozen storage are provided in the pharmacy manual. Within 4 hours of its removal from storage, GNOS-PV02 is mixed with INO-9012 according to the IB and administered to the patient (detailed instructions provided in the IB and Pharmacy Manual). Any temperature or storage deviations should be recorded in the drug accountability records.

The CELLECTRA® 2000 device should be stored in a secure location at room temperature.

Pembrolizumab will be maintained as per the manufacturer's specification.

8.3. Randomization and Blinding

This is an open-label, single-arm study; there will be no randomization or blinding.

All subjects who sign the informed consent form (ICF) will be assigned an 8-digit subject study number which will be retained for the duration of the study. GT-30-2 +1-digit unique site no. + 2-digit subject no. (GT-30-2101).

8.4. Concomitant Therapy

All treatments, including any prescription or over-the-counter medications (e.g., herbals, supplements, vitamins, cannabis, etc.) taken by the subjects 7 days prior to screening, and at any time during the study, are regarded as concomitant treatments and must be documented in the appropriate section of the eCRF. At subsequent visits, changes to current medications or medications used since the last documentation of medications will be recorded. Concomitant medications will be collected until 30 days after the last dose of study medication or until the start of a new anti-cancer treatment, whichever comes first.

8.4.1 Allowed concomitant treatments

The following concomitant treatments are permitted during this study:

- Supportive treatment will be given as medically indicated.
- Prophylactic antiemetic premedication including corticosteroids (low dose ≤10 mg/day prednisone equivalent) and 5 hydroxytryptamine 3 antagonists.
- Supportive treatment with cannabis will be allowed, if medically indicated.
- EMLA (topical lidocaine 2.5% and prilocaine 2.5%) or anxiolytics (e.g., lorazepam [Ativan]) may be used. A pain reliever (e.g., acetaminophen/paracetamol) 30 minutes prior to or after the electroporation procedure may be used.

8.4.2 Prohibited Medications or Treatments During Study

Medications such as those listed below are not permitted in the course of the study:

- Concurrent treatment with other investigational drugs.
- Live vaccines.
- Corticosteroids, with the exception of 10mg/day prednisone or equivalent for acute management of pain (48h or less), discomfort or other clinical symptoms
- Concurrent treatment with any other anticancer therapy including radiotherapy.
- Traditional herbal medicines should not be administered because the ingredients of many herbal medicines are not fully studied, and their use may result in unanticipated drug-drug interactions that may cause or confound assessment of toxicity. However, if the investigator feels herbal medication is warranted, the Sponsor should be consulted.
- Initiation or increased dose of granulocyte colony-stimulating factors (e.g., granulocyte colony stimulating factor, granulocyte/macrophage colony-stimulating factor, and/or pegfilgrastim) is strongly discouraged.

Subjects are not allowed to receive immunostimulatory agents, including but not limited to IFN-α, IFN-γ, or IL-2, during the entire study. These agents, in combination with study treatment, could potentially increase the risk for autoimmune conditions.

8.4.3 Rescue Medication

This is the first time the GNOS-PV02 + INO-9012 is given in combination with pembrolizumab to any subjects. Although most immune-mediated AEs observed with immunomodulatory agents have been mild and self-limiting, such events should be recognized early and treated promptly to avoid potential major complications. Discontinuation of GNOS-PV02 + INO-9012 or pembrolizumab may not have an immediate therapeutic effect due to the long half-life of the drug or longer drug effect, and there is no available antidote for the study treatment. In severe cases, immune-mediated toxicities may be acutely managed with topical corticosteroids, systemic corticosteroids, mycophenolate, or TNF-α inhibitors per investigator's discretion.

For instruction on how to handle pembrolizumab immune response adverse events refer to the pembrolizumab package insert.

Subjects should receive appropriate medical intervention necessary to treat medical conditions as they arise.

8.5. Dose Modification and/or Interruption

For pembrolizumab, no dose modifications are allowed.

For GNOS-PV02 + INO-9012, there will be no dose reductions allowed for this study.

In case of clinically significant AEs, the dosing interval of 3 weeks can be extended to up 42 days to allow the recovery from a related toxicity, and the subject will resume at the same dose. If the subject experiences the same grade or higher toxicity and same grade requiring a dose delay at the subsequent cycle, the subject should be discontinued from study treatment.

Treatment with GNOS-PV02 + INO-9012 may continue if pembrolizumab is discontinued by the investigator, if prior to 36 months. Treatment with pembrolizumab may continue if GNOS-PV02 + INO-9012 is discontinued by the investigator, if prior to 24 months. If both study treatments are stopped for >42 days, then the subject should be discontinued from study treatment and continue to the study follow-up phase.

9. STUDY INTERVENTION DISCONTINUATION AND PARTICIPANT DISCONTINUATION/WITHDRAWAL

9.1. Discontinuation of Study Intervention

Discontinuation for the study treatment does not mean discontinuation from the study, and the remaining study procedures should be completed as per the Schedule of Assessments (Table 3).

9.2. Participant Discontinuation/Withdrawal from the Study

The subjects may withdraw from study treatment if they decide to do so, at any time, and irrespective of the reason. In addition, the Investigator or the Sponsor has the right to withdraw the subject from the study and/or stop the study at any time. All efforts should be made to document the reason for discontinuation, and this should be documented in the eCRF.

Other criteria for possible discontinuation are:

- Disease progression not meeting criteria for dosing of study treatment beyond disease progression (see Section 10.1.1, Tumor Assessment)
- Unacceptable toxicity as judged by the Principal Investigator
- AEs which are DLTs
- Withdrawal of consent

- Subject is lost to follow-up
- Subject non-compliance
- Use of another non-protocol anti-cancer treatment
- Pregnancy
- Completed 36 months of study treatment

Withdrawn subjects will be followed according to the study procedures as specified in this protocol.

The subjects may withdraw from the study follow-up period, before study completion if they decide to do so, at any time and irrespective of the reason. The reason for withdrawal from the study treatment or study follow-up will be documented in the eCRF.

A subject who receives at least 1 study treatment and who discontinues from the study for any reasons will not be replaced.

9.3. Lost to Follow-up

The Investigator should make every effort to re-contact the subject, to identify the reason why he/she failed to attend the visit, and to determine his/her health status, including at least his/her vital status. Attempts to contact such subjects must be documented in the subject's records (e.g., times and dates of attempted telephone contact, receipt for sending a registered letter). It is suggested that the Investigator attempts to contact the subject 3 times before considering the subject lost to follow-up.

10. STUDY ASSESSMENTS AND PROCEDURES

Refer to the Schedule of Assessments in Table 2 for an outline of the procedures required at each visit along with their associated windows. All subjects must sign and date the most current approved ICF before any study-specific procedures are performed. Procedures conducted as per standard of care or routine clinical management that are obtained before signing of the ICF may be utilized for screening/baseline purposes. All screening assessments may be performed within 28 days of Day 0, with the exception of screening laboratory assessments (hematology and chemistry) which may be performed within 10 days of Day 0 or imaging assessments performed within 21 days. Subjects who discontinue will be asked to return to the clinic within 30 days of the last dose for a discontinuation visit. Generally, protocol waivers or exemptions will not be granted without discussion with the Sponsor.

10.1. Efficacy Assessments

10.1.1 Tumor Assessment

Initial (screening) tumor assessments must be performed within 21 days prior to the first dose of study treatment. The investigator/site radiologist must review pre-study images to confirm the subject has measurable disease per RECIST 1.1 (Appendix B). Tumor assessments performed as standard of care prior to obtaining informed consent and within 21 days of the first dose of study treatment may be used rather than repeating tests. Beginning with screening, all imaging assessments will be evaluated using RECIST 1.1. On-study imaging assessments will be performed Q9W until week 99, then Q12W for 5 years and Q26 until end of study treatment for patients without progression calculated from the date of therapy initiation and independent of treatment delays. RECIST 1.1 will be used by the site for treatment decisions until the first radiologic evidence of PD.

Following the first radiologic evidence of PD by RECIST 1.1, treatment decisions may be made by using immune iRECIST (Appendix B) to accommodate tumor response patterns seen with checkpoint inhibitor

therapy including pembrolizumab treatment (e.g., tumor flare). This was described by Nishino, et al. 2016(48) and is used in immunotherapy clinical studies. For a clinically stable subject with first radiologic evidence of PD, it is at the discretion of the site investigator to continue treating the subject with GNOS-PV02 + INO-9012 and pembrolizumab until PD is confirmed at least 4 weeks after the date of the first tumor imaging suggesting PD, per the site investigator. If radiologic PD is confirmed by the subsequent tumor imaging, the subject should be discontinued from treatment unless, in the opinion of the investigator, the subject is achieving a clinically meaningful benefit. In this case, an exception for continued treatment may be considered following consultation with the Sponsor. Additional treatment response evaluation by RECIST 1.1 and iRECIST may be performed at the Sponsor's discretion.

Subjects will undergo tumor assessments approximately Q9W for the first 54 weeks (approximately 12 months) following first dose of study treatment, or earlier if clinically indicated. After 54 weeks, tumor assessments will be required every 12 weeks (±7 days). After 5 years, tumor assessments will be done every 26 weeks, consistent with SOC imaging monitoring. Imaging should continue to be performed until disease progression is assessed by the Investigator, the start of new anti-cancer treatment, withdrawal of consent, death or the end of the study, whichever occurs first for efficacy follow-up. Subjects who start a new anti-cancer therapy will be censored for survival and progression analyses at date of last scan prior to the start of new anti-cancer therapy.

Tumor imaging should be performed by CT, but may be performed by magnetic resonance imaging (MRI), but the same imaging technique should be used in subject throughout the study. CT scans (with oral/IV contrast unless contraindicated) must include chest, abdomen and pelvis. The investigator must review before dosing at the next visit. Per RECIST 1.1, CR and PR responses should be confirmed by a repeat radiographic assessment. The scan for confirmation of response may be performed no earlier than 4 weeks after the first indication of response, but within 6 weeks after the response is detected.

Subjects who have unconfirmed disease progression may continue on-treatment until progression is confirmed. If radiologic imaging by local/site assessment shows first evidence of PD, tumor assessment may be repeated 4 weeks later in order to confirm PD, with the option of continuing treatment while awaiting radiologic confirmation of progression. If repeat imaging shows SD, PR (partial response), or CR, treatment may be continued as per treatment schedule. If repeat imaging still meets the threshold for PD (≥20% increase in tumor burden compared to nadir) but shows a reduction in tumor burden compared to the previous time point, treatment may be continued as per treatment calendar after consultation with Sponsor. If repeat imaging confirms PD without reduction in tumor burden compared to the previous time point, subjects will be discontinued from study treatment.

The decision to continue study treatment after the first evidence of disease progression or after confirmation of PD is at the investigator's discretion based on the clinical status of the subject as described in Table 8. Confirmatory imaging may be performed as early as 28 days later; alternatively, the scan performed at the next scheduled time point may be used as confirmation. Subjects may receive study treatment while waiting for confirmation of PD or after confirmation of PD if they are clinically stable as defined by the following criteria:

- Absence of signs and symptoms (including worsening of laboratory values) indicating disease progression
- No decline in ECOG performance status from baseline
- Absence of rapid progression of disease
- Absence of progressive tumor at critical anatomical sites (e.g., cord compression) requiring urgent alternative medical intervention

- Evidence of clinical benefit (defined as the stabilization or improvement of disease- related symptoms) as assessed by the investigator

Table 8. Imaging and Treatment After First Radiologic Evidence of Progressive Disease

	**Clinically Stable**		**Clinically Unstable**	
	**Imaging**	**Treatment**	**Imaging**	**Treatment**
First radiologic evidence of PD	Repeat imaging at ≥ 4 weeks at site to confirm PD	May continue study treatment at the investigator's discretion while awaiting confirmatory scan by site	Repeat imaging at ≥4 weeks to confirm PD per physician discretion only	Discontinue Treatment
Repeat scan confirms PD (no reduction in tumor burden from prior scan)	No additional imaging required	Discontinue treatment, may continue treatment at investigators discretion and sponsor approval if clinical benefit	No additional imaging required	NA
Repeat scan confirms PD (reduction in tumor burden from prior scan)	Continue regularly scheduled imaging assessments	Continue study treatment after consultation with Sponsor	Continue regularly scheduled imaging assessments	May restart study treatment if condition has improved and/or clinically stable per investigator and Sponsor's discretion
Repeat scan shows SD, PR or CR	Continue regularly scheduled imaging assessments	Continue study treatment at the investigator's discretion	Continue regularly scheduled imaging assessments	May restart study treatment if condition has improved and/or clinically stable per investigator's discretion

CR = complete response; NA = not applicable; PD = progressive disease; PR = partial response

Subjects in whom radiographic disease progression is confirmed at a subsequent tumor assessment may be considered for continued study treatment at the discretion of the investigator if they continue to meet the criteria above and have evidence of the clinical benefit (see Figure 5).

Figure 5. Conditions for Continuing Study Treatment in the Presence of Increased Radiographic Tumor Size

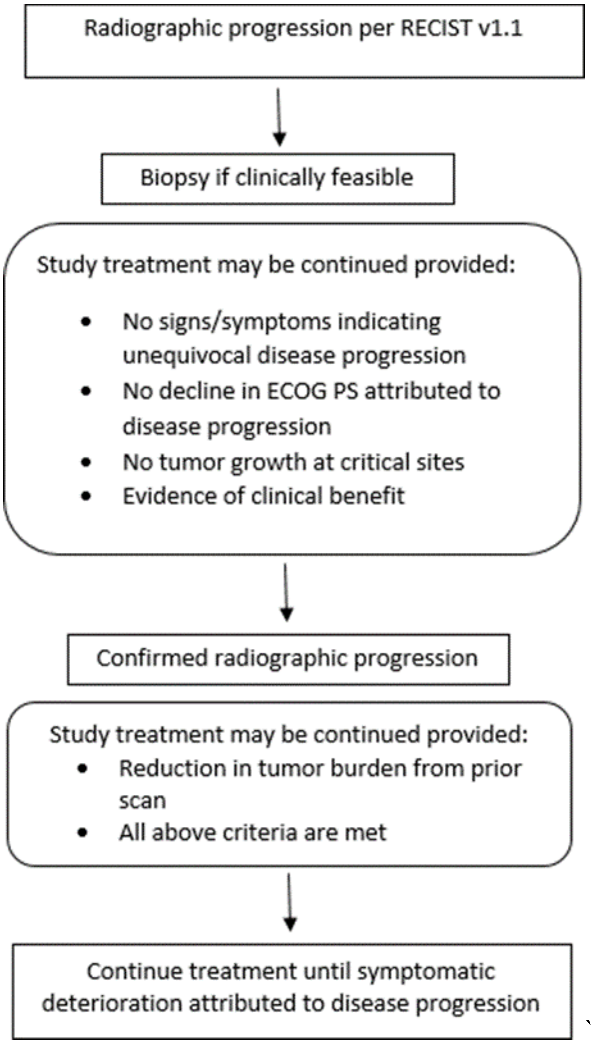

Subjects who discontinue from treatment for reasons other than disease progression (e.g., toxicity) will continue scheduled tumor assessments until disease progression, start of new-anti cancer therapy, withdrawal of consent, or death. Investigators may perform additional scans or more frequent assessments if clinically indicated. Subjects who continue treatment beyond radiographic or clinical disease progression will be monitored with a follow-up scan at the next scheduled tumor assessment.

Imaging timing should follow calendar days and should not be adjusted for delays or changes in treatment administration dates.

10.2. Safety Assessments

10.2.1 Demographics and Medical History

Demographics will include gender, year of birth, race and ethnicity.

Medical history will include details regarding the subjects overall medical and surgical history, as well as detailed information regarding the subject's previous treatment, including systemic treatments, radiation and surgeries, pathology, risk stratification, and other information since their original diagnosis. Reproductive status and smoking/alcohol/IV drug use history will also be captured.

10.2.2 Physical Examinations

A complete physical exam will include, at a minimum head, eyes, ears, nose, throat and cardiovascular, dermatological, musculoskeletal, respiratory, gastrointestinal and neurological systems. Height (screening only) and weight will also be collected. Additionally, any signs and symptoms, other than those associated with a definitive diagnosis, should be collected at Day 0 and during the study.

During the study, a targeted, symptom-directed exam, as clinically indicated will be performed within 72 hours of each dosing visit

10.2.3 Eastern Cooperative Oncology Performance Status

The health, activity and well-being of the subject will be measured by the ECOG performance status and will be assessed on a scale of 0 to 5 with 0 being fully active and 5 being dead. Full details are described in Appendix C. ECOG performance status will be collected within 72 hours of each dosing visit.

10.2.4 Vital Signs

Vital signs will include temperature, blood pressure, pulse rate and respiratory rate. For first infusion of pembrolizumab, the subject's vital signs should be determined within 60 minutes before the infusion. If clinically indicated, vital signs should be recorded at 15, 30, 45, and 60 minutes (± 5 minutes for all time points) after the start of the infusion, and 30 (± 10) minutes after the infusion. For subsequent infusions, vital signs will be collected within 60 minutes before the infusion and at 30 (± 5) minutes after the infusion. Subjects will be informed about the possibility of delayed post-infusion symptoms and instructed to contact their study physician if they develop such symptoms.

10.2.5 Electrocardiograms

A 12-lead ECG will be obtained at screening and when clinically indicated. Subjects should be resting in a supine position for at least 10 minutes prior to ECG collection.

10.2.6 Clinical Safety Laboratory Assessments

Hematological toxicities will be assessed in terms of hemoglobin value, white blood cell, neutrophil, platelet, and lymphocyte count according to CTCAE version 5.0 AE grading.

Laboratory abnormalities (Grade 1 and greater that are listed in the CTCAE version 5.0) should be recorded on the AE page regardless of their causality. Laboratory abnormalities associated with a definitive diagnosis will not be recorded as and AE unless it has become worse since baseline. Test analytes are provided in Table 9.

See the Schedule of Assessments (Table 3) for timing and frequency. Safety laboratory assessments will be performed within 72 hours of each dosing visit.

Table 9. Test Analytes for Laboratory Assessments

Hematology<br>Hematocrit (Hct)<br>Hemoglobin (Hgb)<br>Platelet count<br>Red blood cell (RBC) count<br>White blood cell (WBC) count<br>Neutrophils<br>Lymphocytes<br>Eosinophils<br>Monocytes<br>Basophils<br>Other cells, if any<br>Platelets<br><br>Thyroid<br>TSH, T3 and FT4<br><br>Coagulation<br>International normalized ratio (INR)<br>Activated partial thromboplastin time (PTT)<br>Other anticoagulant monitoring (if required)<br><br>HIV screen (at screening, if indicated)<br>Hepatitis screen (at screening, if indicated)<br>Pregnancy test	Serum chemistry<br>Albumin<br>Alanine aminotransferase (ALT)<br>Aspartate aminotransferase (AST)<br>Alkaline phosphatase (ALP)<br>Blood Urea Nitrogen (BUN) or Urea<br>Bicarbonate or Carbon dioxide (CO2)<br>Creatinine<br>Electrolytes (Na, K, Mg, Cl, Ca, P)<br>Glucose (either fasting or non-fasting)<br>Lactate dehydrogenase (LDH)<br>Total bilirubin (direct bilirubin if elevated)<br>Total protein

10.2.7 Hepatitis and HIV Screening

Subjects should be tested for HIV locally prior to the inclusion into the study only based on investigator's clinical suspicion for HIV infection and HIV-positive subjects will be excluded from the clinical study. HBsAg, anti-HBc antibody, anti-HBs antibody, and Hepatitis C antibody immunoassays should be tested only per investigator's clinical suspicion during screening and tested locally. In subjects who have positive serology for the anti-HBc antibody, HBV DNA should be tested prior to Day 0.

10.2.8 Pregnancy Test

A pregnancy test (for women of childbearing potential, including women who have had a tubal ligation) must be performed and documented as negative within 72 hours prior to each dose. Urine test is valid if negative or positive result. Serum test is required if urine is indeterminate.

10.2.9 TSH, T3, and FT4

Thyroid function tests will be performed at screening and every 3 weeks from Day 0 thereafter. Free T3 can be used as alternative if T3 is not available.

10.2.10 Immunogenicity Assessments

Detailed instructions on the collection and processing of immunogenicity samples will be located in the Laboratory Manual. See the Schedule of Assessments (Table 3) for a collection timeline for each sample.

10.2.10.1 Blood Assays

Blood assays include those measured in serum, plasma and whole blood/peripheral blood mononuclear cells.

10.2.10.1.1. Serum and plasma

Serum and plasma are collected for the direct measure of various tumor biomarkers.

10.2.10.1.2. Tumor and immune biomarkers

Levels of various cancer biomarkers and cytokines may also be tested in serum and/or plasma and may also be used to monitor cancer status and response to treatment. MicroRNA profiling of pre- and post-treatment serum and/or plasma samples may also be performed to predict treatment efficacy.

10.2.10.1.3. Circulating tumor DNA (ctDNA)

Blood samples will be collected in Streck tubes for isolation of ctDNA. ctDNA analysis may be used as a tool to monitor for treatment efficacy and resistance and for predicting the likelihood of relapse.

10.2.10.1.4. Whole blood/peripheral blood mononuclear cells (PBMCs)

PBMCs are collected to monitor overall and specific immune responses.

10.2.10.1.5. Immunophenotyping

Immunophenotyping will be performed by flow cytometry to monitor the levels of all immune cells including B-cells, CD4+ T-cells, CD8+ T-cells, natural killer cells, monocytes, neutrophils, eosinophils, and MDSCs. In subjects mounting an active immune response it is expected for the percentages of certain cell types to increase.

Gene transcript signatures from PBMCs to assess the profile of immune-related gene transcripts may be performed on PBMCs with or without prior *in vitro* stimulation.

10.2.10.1.6. T-cells

T-cells form the cellular arm of the immune response. Vaccination with GNOS-PV02 + INO-9012 is expected to result in maturation and activation of antigen specific T-cells.

10.2.10.1.7. T-cell profiling

The cellular immune response can generally be characterized as having two primary arms, CD4+ helper T-cell responses and CD8+ cytotoxic T-cell responses. In preclinical studies of neoantigen vaccines, activation of both T-cell subsets was noted. Furthermore, immune responses are often hampered by the presence of regulatory T-cells which may downregulate T-cell responses. Multi-parameter flow cytometry will be used to characterize the various subsets of T-cells in peripheral blood during the entire course of the study. Flow cytometric assays will also be utilized to assess the presence of cells that are known to play a role in immune suppression and may include an examination of the influence of these cells on the induction or expansion of an immune response after immunotherapy. Markers that may be used for this purpose include CD3, CD16, CD19, CD20, CD56, CD11b, CD14, CD15, CD33 and HLA-DR. These markers may change relative to new data becoming available that is informative for this assessment.

10.2.10.1.8. Neoantigen-specific T-cells

T-cell responses will be assessed using antigen-specific IFN-γ Enzyme Linked Immunosorbent Spot-forming assay using antigen presenting cells loaded with overlapping peptide libraries covering the neoantigens included in GNOS-PV02. Antigen specific T-cell responses will also be assessed via flow cytometry. Flow cytometric assays may include an examination of the influence of immunotherapy on the ability of subject T-cells to exhibit phenotypic markers associated with cytolytic potential, activation or exhaustion after stimulation by peptides corresponding to GNOS-PV02 encoded neoantigens. Markers that may be used for this purpose include CD3, CD4, CD8, CD137, CD69, CD38, PD1, Granzyme A, Granzyme B, and Perforin. These markers may change relative to new data becoming available that is informative for this assessment. Additionally, T-cell responses to general immune stimulators may be evaluated in order to track general cellular immune competence during the study.

Additionally, neoantigen-specific T-cells may be isolated, cloned and expanded ex vivo. For expansion antigen presenting cells loaded with either full-length recombinant proteins or overlapping peptide libraries covering the neoantigens in GNOS-PV02 would be employed. These T-cells may be characterized by sequencing of their T-cell receptors (TCR) to assess diversity and putative antigen specificity.

10.2.10.2 Tissue

A fresh or archival tumor specimen should be obtained at screening. In subjects with recurrent disease, an archival tumor specimen, if available, should also be submitted. After signing of the ICF, tumor tissue should be submitted to the Sponsor in a timely manner along with 3 mL of whole blood collected in an EDTA tube. All subjects will undergo a mandatory tumor biopsy sample collection, if clinically feasible as determined by the study investigator in consenting subjects, at Week 9 (dose 4 [±3 days]) and at the time of first evidence of radiographic or clinical disease progression. For subjects who respond and subsequently progress, an optional biopsy may be obtained at the time of disease progression. Tumor tissue should be of good quality based on total and viable tumor content. Acceptable samples include core needle biopsies for deep tumor tissue or lymph nodes or excisional, incisional, punch, or forceps biopsies for cutaneous, subcutaneous, or mucosal lesions. Fine-needle aspiration may be acceptable pending Sponsor approval; however, brushing, cell pellets from pleural effusion, and lavage samples are not acceptable. For core needle biopsy specimens, at least 3 cores should be submitted for evaluation, either fresh-frozen, frozen in RNAlater or FFPE. Subjects who are unable to undergo biopsy sample collection, but otherwise meet criteria outlined in protocol, may continue to receive study treatment.

If a tumor biopsy is to be obtained from an intended target lesion during eligibility assessment, the biopsy should be performed prior to obtaining the baseline scan. Otherwise, a new baseline scan should be obtained.

Archival and fresh tumor tissue samples should be representative tumor specimens and submitted fresh-frozen, in FFPE blocks or at least 15 unstained slides, with an associated pathology report, should be submitted for intra-tumoral immunology assessments. Tissue slices of 4-5 µm are mounted on positively charged glass slides. Slides should be unbaked and stored cold or frozen.

10.2.10.2.1. Tissue Assays

Available tumor tissue collected at the time of enrollment (pre-treatment sample) and on-treatment sample after multiple doses of GNOS-PV02 + INO-9012 + pembrolizumab may be assessed for the presence of immune cells using immunohistochemistry or immunofluorescence. The presence of immune signatures may also be analyzed through the assessment of various transcripts suggestive of an inflammatory or an immunosuppressive tissue microenvironment.

Tumor tissue will be collected for immunology assessments, including but not limited to, markers related to inflammation, suppression, T-cell infiltration, and associated tumor microenvironment characteristics. Tumor infiltrating lymphocytes may be isolated and subjected to single cell expression profiling and/or TCR sequencing.

In addition, exploratory biomarkers may be evaluated.

10.2.10.2.2. Future Biomedical Research

The following samples are obtained as part of the study. If any leftover samples remain, they may be used for FBR either during the course of the study or after the study has completed.

- Leftover tumor tissue
- Leftover RNA or DNA isolated from biological samples (blood, tumor)
- Leftover biomarker samples (serum, plasma, and peripheral blood mononuclear cells [PBMCs])

10.2.10.2.3. Withdrawal from Future Biomedical Research

Subjects may withdraw their consent for FBR and have their samples destroyed. Subjects may withdraw consent at any time by contacting the principal investigator in writing. In turn, the Investigator will contact the Sponsor in writing. Subsequently, the subject's samples will be removed from the biorepository and be destroyed. No future data will be collected, but any data already collected from analysis of samples will remain with the Sponsor. A letter will be sent from the Sponsor to the investigator confirming the destruction. It is the responsibility of the investigator to inform the subject of completion of destruction. Any analyses in progress at the time of request for destruction or already performed prior to the request being received by the Sponsor will continue to be used as part of the overall research study data and results. No new analyses would be generated after the request is received.

In the event that the medical records for the study are no longer available (e.g., if the investigator is no longer required by regulatory authorities to retain the main study records) or the samples have been completely anonymized, there will no longer be a link between the subject's personal information and their samples. In this situation, the request for sample destruction cannot be processed.

10.2.10.3 Concomitant Medications

Concomitant medications include any prescription medications or over-the-counter medications. At screening, any medications the subject has used within the 7 days prior to the screening visit should be documented. At subsequent visits, changes to current medications or medications used since the last documentation of medications will be recorded. See Section 8.4.2 for a list of prohibited medications.

10.2.10.4 Adverse Events

AEs will be collected from the time of first dose of study treatment until the patient reaches the end of the study. See Section 10.3 for additional details on AEs and SAEs.

10.2.10.5 Follow-Up

Survival follow-up information will be collected via telephone calls, subject medical records, and/or clinical visits approximately every 3 months for up to 4 years, until death, lost to follow-up, withdrawal of consent, or study termination by Sponsor. All subjects will be followed for survival and new anticancer therapy information unless the subject requests to be withdrawn from follow-up; this request must be documented in the source documents and signed by the investigator. If the subject discontinues study treatment without

documented clinical disease progression, every effort should be made to follow up regarding survival, progression (if not already progressed), and new anti-cancer therapy.

10.3. Adverse Events and Serious Adverse Events

10.3.1 Definition of Adverse Event

An AE is defined as any untoward medical occurrence associated with the use of a drug in humans, whether or not considered drug related and occurs after the subject is given the first dose of study drug. Any AE that occurs prior to the first dose is part of the medical history.

Abnormal laboratory values should not be listed as separate AEs if they are considered to be part of the clinical syndrome that is being reported as an AE, unless worsened on study treatment. It is the responsibility of the investigator to review all laboratory findings in all subjects and determine if they constitute an AE. Medical and scientific judgment should be exercised in deciding whether an isolated laboratory abnormality should be classified as an AE. Any laboratory abnormality (Grade 1 and greater that are listed in the CTCAE version 5.0) considered to constitute an AE should be reported on the AE case report form (CRF).

Pre-planned procedures (surgeries or therapies) that were scheduled prior to the start of study drug exposure are not considered AEs. However, if a pre-planned procedure is performed earlier than anticipated (e.g., as an emergency) due to a worsening of the pre-existing condition, the worsening of the condition should be captured as an AE

Progression of the cancer under study is not considered an AE unless it is considered to be drug related by the investigator. Subjects will be encouraged to spontaneously report any AE. Subjects will be questioned and/or examined by the investigator and his/her medically qualified designee for evidence of AEs. The questioning of study subjects with regard to the possible occurrence of AEs will be generalized, such as, "How have you been feeling since your last visit?" Information gathering for AEs should generally not begin with direct solicitation from subjects regarding the presence or absence of specific AEs. Study personnel will ask open-ended questions to obtain information about AEs at every visit. Date and time of onset and resolution (if applicable) of the AE will be documented in the subject's clinical notes.

A suspected adverse reaction means any AE for which there is a "reasonable possibility" that the drug caused the AE. For the purpose of reporting under this protocol, "reasonable possibility" means there is evidence to suggest a causal relationship between the drug and the AE.

An AE is considered unexpected if the AE is not listed in the current IB or is not listed in the IB at the specificity or severity observed.

10.3.2 Definition of Serious Adverse Event

A serious adverse event (SAE) is an AE that:

- Is fatal or,
- Is life-threatening, meaning the subject was, in the view of the investigator, at immediate risk of death from the reaction as it occurred (i.e., it does not include a reaction that, had it occurred in a more serious form or progressed, might have caused death) or,
- Is a persistent or significant disability or incapacity or substantial disruption of the ability to conduct normal life functions or,
- Requires or prolongs inpatient hospitalization or,
- Is a congenital anomaly or birth defect or,

- Other important medical events may be considered SAEs when, based upon appropriate medical judgment, they may jeopardize the subject and may require medical or surgical intervention to prevent one of the outcomes as listed above in this definition. Examples of such medical events include allergic bronchospasm requiring intensive treatment in an emergency room or at home, blood dyscrasias or convulsions that do not result in inpatient hospitalization, or the development of drug dependency or drug abuse.

The Medical Monitor will advise the investigator regarding the nature of any further information or documentation that is required. The investigator should provide the following documentation at the time of notification if available:

- SAE Form
- AE (CRF) page
- Concomitant and support medication pages
- Relevant diagnostic reports
- Relevant laboratory reports
- Admission notes and hospital discharge summary (when available)

Clarification of Serious Adverse Events:

- Death in itself is not an AE. Death is an outcome of an AE.
- Progression of the cancer under study is not considered an AE unless it is considered to be drug related by the investigator.
- The subject may not have been receiving an investigational medicinal product at the occurrence of the event.
- Dosing may have been given as treatment cycles or interrupted temporarily before the onset of the SAE but may have contributed to the event.
- Complications that occur during hospitalizations are AEs. If a complication prolongs the hospitalization, it is an SAE.
- Inpatient hospitalization means that the subject has been formally admitted to a hospital for medical reasons, for any length of time. This may or may not be overnight. It does not include presentation and care within an emergency department, nor does it include full-day or overnight stays in observation status.

    The following hospitalization scenarios are not considered to meet the criteria for SAEs:

    - Hospitalization for respite care
    - Hospitalization to perform an efficacy measurement for the study
    - Hospitalization for an elective surgery for a pre-existing condition

The investigator will attempt to establish a diagnosis of the event on the basis of signs, symptoms, and/or other clinical information. In such cases, the diagnosis will be documented as the AE and/or SAE and not the individual signs/symptoms.

10.3.3 Classification of an Adverse Event

10.3.3.1 Severity of Event

AEs will be graded by the investigator using the CTCAE version 5.0, graded 1-5. Grade refers to the severity of the AE. For events not described in the CTCAE, the investigator will assign grades as 1=mild, 2=moderate, 3=severe, 4=life-threatening, and 5=fatal based on the general guideline in Table 10.

Table 10. Guidelines for Adverse Event Severity

**Grade 1**	Mild; asymptomatic or mild symptoms; clinical or diagnostic observations only; intervention not indicated.
**Grade 2**	Moderate; minimal, local or noninvasive intervention indicated; limiting age-appropriate instrumental ADL[a]
**Grade 3**	Severe or medically significant but not immediately life-threatening; hospitalization or prolongation of hospitalization indicated; disabling; limiting self-care ADL[b]
**Grade 4**	Life-threatening consequences; urgent intervention indicated.
**Grade 5**	Death related to AE. (Grade 5 [Death] may not appropriate for some AEs and therefore may not an option.)

ADL = activities of daily living; AE = adverse event

[a] Instrumental ADL refer to preparing meals, shopping for groceries or clothes, using the telephone, managing money, etc.

[b] Self-care ADL refer to bathing, dressing and undressing, feeding self, using the toilet, taking medications, and not bedridden.

10.3.3.2 Relationship to Study Medication

An AE relationship will be assessed as based on GNOS PV02 + INO-9012, CELLECTRA® 2000 administration and/or pembrolizumab. Association of events to the study treatment will be made using the following definitions:

> *All AEs are to be assessed for causal relationship with the one or more of IPs and/or the investigational device. An AE may be assessed as related or not related to the IP and/or the investigational device. Because the Investigator is knowledgeable about the subject (e.g., medical history, concomitant medications), administers the IP, and monitors the subject's response to the IP, the Investigator is responsible for reporting AEs and judging the relationship between the administration of the IP and EP and a subsequent AE. The Sponsor will assess the overall safety of the IP delivered by EP and determine whether to report expeditiously to the regulatory agencies.*
>
> *Investigators should use their knowledge of the Trial Subject, the circumstances surrounding the event, and an evaluation of any potential alternative causes to determine whether or not an AE is considered to be related to the IP and/or the investigational device indicating "yes" or "no" accordingly, and should provide justification of causality as "yes, related" or "no, unrelated" by the following criteria:*
>
> *Yes – there are facts, evidence or arguments that administration of the Trial Treatment (drug or device or both drug and device) contributed to the event;*

No – there are no facts, evidence or arguments that administration of the Trial Treatment contributed to the event.

The following should also be taken into consideration:

- Temporal relationship of event to administration of IP and/or EP;
- Course of the event, considering especially the effects of dose reduction, discontinuation of IP, or reintroduction of IP (where applicable);
- Known association of the event with the IP, EP or with similar treatments;
- Known association of the event with the disease under trial or any co-morbidities;
- Presence of risk factors in the Trial Subject or use of concomitant medications known to increase the occurrence of the event.

The assessment of relationship of AEs to the administration of study drug is a clinical decision based on all available information at the time of the completion of the CRF. The following categories will be used to define the causality of the AE. The highest level of relatedness attained for each AE will be recorded in the CRFs.

10.3.4 Adverse Event Reporting

The reporting period for non-immune related AEs (without regard to causality or relationship) is comprised of the period following the first dose of the study treatment (Day 0) until the patient reaches the end of the study. Each AE will be assessed to determine whether it meets seriousness criteria. If the AE is considered serious, the Investigator should report this event to the Sponsor within 24 hours of becoming aware of the event.

All AEs will be collected and recorded in the Electronic Data Capture (EDC) system. The Study Report will analyze and summarize all AEs throughout the trial.

10.3.5 Serious Adverse Event Reporting

All SAEs occurring during the course of the clinical study from Day 0 of study therapy through the end of the study will be collected and reported (e-mail) by the investigator to the Pharmacovigilance Team assigned (see contact information below) by completing a SAE Report Form within 24 hours or next business day, whichever is shorter, from the point in time when the Investigative site becomes aware of the SAE. All SAEs must be reported, whether or not considered causally related to the study treatment. The information collected will include subject number, a narrative description of the event and an assessment by the investigator as to the severity of the event, and relatedness to study drug. Include copies of relevant source documents. (e.g., progress notes, autopsy reports, laboratory and diagnostic test results, discharge summaries). Follow-up information on the SAE may be requested by the Sponsor.

All safety information should be reported to: [redacted]

Contact info: email: [redacted]

The minimum required information for an initial report of an SAE is:

- Reporter name and contact number,
- Protocol number,
- Site and subject ID information, and
- The SAE term with a brief summary of the event including the causality assessment, if possible.

Contact the Sponsor-designated Medical Monitor or designee using the contact information and instructions provided supplemental to this protocol.

The Sponsor must notify the regulatory authorities and all participating investigators (i.e., all investigators to whom the Sponsor is providing drug under its INDs or under any investigator's IND) in an IND safety report of potential serious risks, from clinical studies or any other source, as soon as possible, but in no case later than 15 calendar days after the Sponsor determines that the information qualifies for reporting under paragraph (c)(1)(i), (c)(1)(ii), (c)(1)(iii), or (c)(1)(iv) of 21CFR 312.32. The Sponsor must identify all IND safety reports previously submitted to the regulatory authorities concerning a similar suspected adverse reaction and must analyze the significance of the suspected adverse reaction in light of previous, similar reports or any other relevant information.

In addition, The Sponsor must also notify the regulatory authorities of any unexpected fatal or life-threatening suspected adverse reaction as soon as possible but in no case later than 7 calendar days after the Sponsor's initial receipt of the information.

10.3.6 Serious and unexpected suspected adverse reaction

The Sponsor must report any suspected adverse reaction that is both serious and unexpected. The Sponsor must report an adverse event as a suspected adverse reaction only if there is evidence to suggest a causal relationship between the drug and the AE, such as:

1. A single occurrence of an event that is uncommon and known to be strongly associated with drug exposure (e.g., angioedema, hepatic injury, Stevens-Johnson Syndrome)
2. One or more occurrences of an event that is not commonly associated with drug exposure, but is otherwise uncommon in the population exposed to the drug (e.g., tendon rupture)

An aggregate analysis of specific events observed in a clinical study (such as known consequences of the underlying disease or condition under investigation or other events that commonly occur in the study population independent of drug therapy) that indicates those events occur more frequently in the drug treatment group than in a concurrent or historical control group.

Reports will be made as soon as possible, and in no event later than 7 calendar days if the event is a death or is life-threatening and 15 calendar days for all other reportable events after the Sponsor's initial receipt of the information. Each written notification may be submitted on a CIOMS-I form, a FDA Form 3500A, or in a tabular or narrative format in accordance with regulatory requirements. In each report, the Sponsor will identify all safety reports previously filed concerning a similar suspected adverse reaction and will analyze the significance of the suspected adverse reaction in light of the previous, similar reports.

Follow-up information to a safety report will be submitted as soon as the relevant information is available. If the results of a Sponsor's investigation show that an AE not initially determined to be reportable is, in fact, reportable, the Sponsor will report the suspected AE in a written safety report as soon as possible, but in no event later than 15 calendar days after the determination is made. Results of investigations of other safety information will be submitted, as appropriate, in an information amendment or annual report.

If an investigator receives an IND safety report or other specific safety information (e.g., suspected unexpected serious adverse reaction, summary or listing of SAEs) from the sponsor, the investigator will review and file along with the IB and will notify the IRB/IEC, if appropriate according to local regulations. In these instances, the ICF may need to be revised to inform the subject of any new safety concern.

10.3.7 Unanticipated (Serious) Adverse Device Effect

A unanticipated (serious) adverse device effect (UADE) is any serious adverse effect on health or safety or any life-threatening problem or death caused by, or associated with, a device, if that effect, problem, or death was not previously identified in nature, severity, or degree of incidence in the investigational plan or application (including a supplementary plan or application), or any other unanticipated serious problem associated with a device that relates to the rights, safety, or welfare of subjects.

Per the definition above, a UADE is a type of SAE that requires expedited reporting on the part of the Sponsor. As a reminder, all SAEs, regardless of relationship to device, drug, or procedure are to be reported to Sponsor by the study investigator within 24 hours. Sponsor will assess each device related SAE to determine if anticipated based on prior identification within the investigational plan. The Sponsor may notify a regulatory authority within the time frame specified by local requirements but no later than 10 business days for UADE.

10.3.8 Dose-Limiting Toxicities

DLTs are events (toxicities) specifically identified in this protocol that must be reported in an expeditious manner. Information regarding DLTs should be recorded on a SAE form and faxed or emailed within 24 hours of site personnel becoming aware of the event. DLTs may or may not be serious adverse events as an unexpected adverse event.

Any of the following, if judged to be associated with GNOS-PV02+INO-9012 will be considered a DLT which are based on the CTCAE version 5.0 criteria:

1. Grade 4 non-hematological toxicities (excluding alopecia) of any duration
2. Grade 3 non-hematologic (non-laboratory) toxicity lasting > 3 days despite optimal supportive care
3. Any Grade 3 or Grade 4 non-hematologic laboratory value if: a) medical intervention is required to treat the subject; b) the abnormality leads to hospitalization; c) the abnormality persists for >1 week
4. Grade 4 hematologic toxicity, other than those specified in criteria 5 and 6 below, lasting >7 days
5. Grade 3 or Grade 4 febrile neutropenia of any duration
6. Grade 3 thrombocytopenia in combination with a Grade 3 or greater blood and lymphatic system disorder
7. Grade 3 AST or ALT that is associated with a Grade 2 rise in bilirubin

Additional information regarding reporting DLTs may be required by the Sponsor.

10.3.9 Stopping Rules

If at any point during the course of this clinical study a higher number of subjects than expected by Bayesian analysis (Section 11.7.1) experience a dose-limiting toxicity as defined in Section 10.3.8 deemed related to investigational trial drug(s), enrollment will stop, and the Sponsor Medical Monitor, in addition to the Principal Investigator (PI) and Investigator(s) at the study site(s) will discuss the safety profile of the trial drug(s), and a decision will be made whether to modify trial drug administration schedule or to cease further enrollment. If a change in the protocol is required, enrollment will only be reinitiated after amendment of the protocol and approval of the amended protocol by the IRB.

10.3.10 Adverse Events of Special Interest

10.3.11 There are no defined Adverse Events of Special Interest for this study. Reporting of Pregnancy

If pregnancy occurs in a female subject, or female partner of a male subject while the subject is on treatment or until 5 months after the last dose, the sponsor will be notified within 24 hours of learning of the pregnancy. The pregnancy will be followed until birth or termination. Abnormal pregnancy outcomes (e.g., spontaneous abortion, fetal death, stillborn, congenital anomalies, ectopic pregnancy) are considered SAEs.

10.3.12 Time Period and Frequency for Event Assessment and Follow-Up

All SAEs, including death due to any cause, that occur after the first dose of the study treatment (Day 0) until the patient reaches the end of the study, must be reported to the Medical Monitor immediately upon discovery of the event, using an SAE Form.

All AEs will be collected starting on the first dose of the study treatment (Day 0) until the patient reaches the end of the study.

Any medical condition that begins before the start of study intervention but after obtaining informed consent will be recorded on the Medical History section of the case report form, not the AE section. However, if the subject's condition worsens during the study, the event will be recorded as an AE.

All AEs/SAEs will be captured on the appropriate case report form. Information to be collected includes event description, date of onset, severity, relationship to study intervention and date of resolution.

10.3.13 Safety Committee

To ensure subject safety during the study, a Safety Committee will be formed to monitor safety on a periodic basis. Members of the Safety Committee will include a Safety Expert, Principal Investigator(s) and the Sponsor Medical Monitor. The Safety Committee will meet approximately every 6 months to review safety data collected from the point of first patient in to review safety, or as needed. The safety data will include demographic data, AEs, SAEs, and relevant laboratory data. Following each data review, the Safety Committee will provide recommendations as to whether the study should continue, be amended, or whether the study should be stopped on the basis of safety (i.e., evidence of harm). The final decision will rest with the Sponsor. Any outcomes of these safety reviews that affect study conduct will be communicated in a timely manner to the investigators for notification of the IRBs/IECs.

11. STATISTICAL CONSIDERATIONS

The formal Statistical Analysis Plan (SAP) is contained in the sections that follow. The statistical analysis of the data will be performed by Geneos Therapeutics or its representative.

This is a single-arm, open-label, multi-site I/IIa study of a personalized neoantigen DNA vaccine (GNOS-PV02) and plasmid encoded IL-12 (INO-9012) delivered by ID injection followed by EP using the CELLECTRA® 2000 device in combination with pembrolizumab (MK-3475) in subjects with histologically or cytologically confirmed diagnosis of HCC and who have documented objective radiographic progression or intolerance to sorafenib or lenvatinib. The trial's primary analyses regard the safety and immunogenicity of GNOS-PV02 + INO-9012 in combination with pembrolizumab assessed by CTCAE graded adverse events, PBMC Interferon-γ secreting T lymphocytes and PBMC T-cell activation and cytolytic cell phenotype. The secondary analyses pertain to anti-tumor activity as assessed by ORR by RECIST version 1.1, ORR by iRECIST, Duration of Response (DoR), DCR, PFS as assessed by RECIST version 1.1 and iRECIST, and OS. Exploratory analyses will explore the potential relationship between

anti-tumor activity and biomarkers. In addition, exploratory analyses will consider the feasibility of generating personalized neoantigen DNA vaccines for subjects with advanced HCC.

11.1. Statistical Hypotheses

The statistical hypothesis for this trial will evaluate the key secondary endpoint of ORR as defined as the best overall complete or partial response by RECIST version 1.1 by investigator review. Define the true treatment effect for ORR as p, where p denotes the true population of ORR. Then, the hypothesis of superiority to the historical control is $H0: p \leq 0.169$ vs $H1: p > 0.169$.

11.2. Sample Size Determination

Adverse events will be continuously monitored. Among the subjects enrolled in the study, for AEs that occur at an incidence of 20% the probability of observing at least one event is greater than 90%.

To evaluate the secondary endpoint of ORR per RECIST 1.1 by investigator review with a null hypothesis of an ORR of 16.9% and an alternative hypothesis of an ORR of 33.1%, 36 subjects will be enrolled using an exact 1-sided binomial test of a single proportion. The overall power for this test with 36 subjects is 80% assuming a one-sided α=0.10. A statistically significant difference at the one-sided 5% level will be declared for an observed ORR of at least 30.5% for 36 subjects (11/36). The operating characteristics of this design are calculated using the exact binomial distribution.

11.3. Populations for Analyses

The following analysis populations will be used for presentation of the data:

Safety Analysis Set: The safety analysis will be based on the Safety Analysis Set, which comprises all enrolled subjects who receive at least 1 dose of the study treatment or component of the study treatment.

The modified intention to treat (mITT) population includes all subjects who receive at least one dose of the trial treatment and have evaluable baseline scan with measurable disease. The mITT population will be used for analysis of the immunogenicity and secondary efficacy endpoints.

The per-protocol (PP) population is comprised of subjects who receive at least the first 4 doses of the trial treatment, have an evaluable baseline scan with measurable disease and who have no important protocol violations. Analyses on the PP population will be considered supportive of the corresponding mITT population for the analysis of efficacy. Subjects excluded from the PP population will be identified and documented prior to locking the trial database.

11.4. General Statistical Methods

For continuous variables, descriptive statistics (number [n], mean, median, standard deviation, minimum, and maximum) will be presented. For categorical variables, frequencies and percentages will be presented. For time-to-event variables, percentages of subjects experiencing that event will be presented and median time-to-event will be estimated using the Kaplan-Meier method. As appropriate, a 95% CI will be presented. Graphical displays will be presented, as appropriate.

Subjects' demographic characteristics, including age, gender, and race, will be analyzed, with categorical variables summarized in frequency tables while continuous variables summarized using mean (standard deviation) and median (range).

All data collected will be presented in by-subject data listings.

11.5. Primary Safety Analyses

The primary analyses for this trial are safety analyses of TEAEs. TEAEs are defined for this trial as any AEs that occur starting on the first dose of the study treatment (Day 0) until the patient reaches the end of the study. Toxicities due to first-line standard of care therapy while awaiting study therapy will not be collected or reported.

All TEAEs will be summarized for the subjects in the safety analysis set by frequency, percentage and 95% Coppler-Pearson confidence intervals. These frequencies will be presented overall and separately by dose number, and will depict overall, by system organ class and by preferred term, the percentage of subjects affected. Multiple occurrences of the same AE in a single subject will be counted only once following a worst-case approach with respect to severity and relationship to trial treatment. Any AEs with missing or partial onset/stop dates will be included in the overall AE summaries but excluded from the calculation of AE duration. AE duration will be calculated as AE stop date - AE start date + 1 day.

All of the safety analyses will be conducted on the subjects in the safety analysis set.

11.6. Primary Immunogenicity Analyses

Neoantigen-specific cellular immune response assessed by, but not limited to, INF-γ secreting T lymphocytes will be summarized by visit using descriptive statistics and changes from baseline. T-cell activation and cytolytic cell phenotype in PBMCs by flow cytometry will also be analyzed in the same manner.

The primary immunogenicity analyses will be conducted on the mITT and Per Protocol populations.

11.7. Secondary Efficacy Analyses

iORR is defined as the proportion of subjects with a confirmed best response of complete response (iCR) or partial response (iPR) by iRECIST. iORR will be summarized using frequency, percentage and 95% Clopper-Pearson confidence interval, including number and percent of subjects in each overall response category. ORR is defined as the proportion of subjects with a confirmed best response of CR or PR by RECIST 1.1. ORR will be analyzed using the same statistical methods that are planned for iORR. iORR and ORR are based on the best objective response for each subject during the entire study.

iRECIST and RECIST responses at each scan visit will also be presented using frequency, percentage, and 95% Clopper-Pearson confidence interval.

All RECIST assessments, whether scheduled or unscheduled, will be included in the calculations. At each scan visit, subjects will be assigned a RECIST version 1.1 visit response of CR, PR, or PD depending on the status of their disease compared to baseline and previous assessments by the investigator. iRECIST will also be assessed at each scan visit and categorized as iCR, iPR, iSD, iUPD (Unconfirmed Progressive Disease), or iCPD (Confirmed Progressive Disease) by the investigator. The baseline scan will be assessed within the 21 days prior to first administration of study treatment. If a subject has had a tumor assessment that cannot be evaluated, the subject will then be assigned a visit response of not evaluable (unless there is evidence of progression in which case the response will be assigned as PD). Additionally, confirmatory scans are requested in the 4-6 weeks timeframe after the first radiographic evidence of PR/CR. After the confirmatory scan is obtained, standard imaging schedule will resume (i.e Q9W)

See Appendix B for the iRECIST and RECIST 1.1 definitions.

RECIST 1.1 assessments/scans contributing towards a particular visit may be performed on different dates. When this happens, the following rule will be applied:

For investigator assessments, the date of progression will be determined based on the earliest of the RECIST 1.1 assessment/scan dates of the component that indicates progression.

Note: For target lesions, only the latest scan date is recorded out of all scans performed at that assessment for the target lesions, and similarly for non-target lesions, only the latest scan date is recorded out of all scans performed at that assessment for the non-target lesions.

Duration of response (DOR), DCR, PFS and OS will also be calculated, defined as follows:

- DOR: time from date of first confirmed response (CR or PR) to date of progression (PD), where subjects without progression are censored at date of last valid disease assessment scan.
- DCR: proportion of subjects with SD or better (PR and CR), that is, no PD.
- PFS: time from date of start of first treatment administration to date of progression or death, whichever occurs first, where subjects without progression are censored at date of last valid disease assessment scan.
- OS: time from date of start of first treatment administration to date of death or censored at date of last contact.

For calculating iDOR, iDCR and iPFS, iCR, iPR, and iCPD will be used.

PFS and OS will be summarized with Kaplan-Meier statistical methods for both iRECIST and RECIST 1.1. Subjects will be censored for PFS at withdrawal of consent, date of new anti-cancer medication, date of withdrawal from the study or the last progression assessment date where the subject was considered to have not progressed. Subjects who are not recorded as having died will be censored for OS at withdrawal of consent or the last data the subject was known to be alive. Censoring rules for DOR will be the same as for PFS.

The Secondary Efficacy Analyses will be performed on subjects in both the mITT and per protocol population.

11.7.1 Safety Stopping Rule

The Stopping Rule will be determined following the proportion of subjects with a particular DLT resulting from study therapy that exceeds a Bayesian stopping guideline. A toxicity level 45% would be considered the upper boundary. We expect the actual toxicity level to be 25%(49). A Beta (2.5, 5.5) prior, representing a toxicity rate of 31% (slightly above our expected rate), will be used to be conservative. After the first 6 patients have been treated, safety will be monitored continuously. If the probability that the proportion of unacceptable toxicities exceeds 45%, we will halt accrual and re-evaluate the trial. The following table shows the number of toxicities that would need to be observed in order to trigger the stopping guidelines throughout the course of the trial. The calculated number of subjects with DLT sufficient to trigger the stopping rule is the following:

Number of Patients	Number of Toxicities Needed to Trigger Re-Evaluation
6	4
7-8	5
9-10	6
11-13	7

14-15	8

11.8. Exploratory Analyses

Tumor and immune biomarkers, which include assessment of myeloid derived suppressor cells (MDSC), T-cell receptor sequencing of PBMCs, immune gene transcript profiling of PBMCs, assessment of pro-inflammatory and immunosuppressive elements in neoplastic and adjacent normal tissue, pre- and post-treatment peripheral blood tumor tissue, tumor-specific oncoproteins, microRNA, cytokine and chemokine profiles, and circulating tumor DNA, will be descriptively summarized by visit.

iORR, ORR, iPFS, PFS and OS will be modeled using logistic regression models and Cox PH models against the exploratory responses to examine associations. Baseline variables such as patient demographics, patient disease characteristics or immune responses will be included in the models as potential confounders.

All these exploratory analyses will be conducted on the mITT and/or Per Protocol populations as appropriate.

11.9. Analysis of Other Safety Data

Laboratory response variables will be descriptively summarized per time point and as changes from baseline including 95% confidence intervals. Laboratory values considered clinically significant will be presented in listings.

The percentage of subjects with abnormal medical history findings will be summarized by body system and preferred term for subjects in the safety population. Prior medications are those that were used before the start of the trial (within 28 days prior to Day 0). Concomitant medications are those used during trial (on or after Day 0). Partial start dates of prior and concomitant medications will be assumed to be the earliest possible date consistent with the partial date. Partial stop dates of prior and concomitant medications will be assumed to be the latest possible date consistent with the partial date. Data for all prior and concomitant medications will be summarized with percentages for each cohort for subjects in the safety population.

Measurements for vital signs as well as changes from baseline will be descriptively summarized by visit and time point for subjects in the safety analysis set. The percentage of subjects with abnormal physical examination findings at each time point will be descriptively summarized overall by body system for subjects in the safety analysis set.

Electrocardiogram (ECG) and viral serology at screening, and pregnancy at each time point will be descriptively summarized.

11.10. Disposition

Subject disposition will be summarized for all enrolled subjects and will include the number and percentage enrolled, the number and percentage who received each planned dose, and the number who completed the trial. The number and percentage of subjects who discontinued treatment will be summarized by reason. The number in each analysis population will also be presented.

11.11. Demographic and Other Baseline Characteristics

Demographic and baseline characteristic data will be descriptively summarized for subjects in the safety and mITT analysis sets.

11.12. Interim Analyses

There is no formal interim analysis built-in to the study. However, safety endpoints will be assessed on an ongoing basis in order to assess Dose Limiting Toxicities.

11.13. Multiplicity

Not applicable; there is one hypothesis that will be tested.

11.14. Missing Data

Missing data will not be imputed or replaced, and calculations will be done on reported values.

11.15. Randomization and Blinding

Because this is an open-label trial, site personnel, individual subjects, and Geneos or its representative trial personnel will be aware of the treatment allocations for this trial. Because this is a single-arm trial, randomization does not apply.

12. SUPPORTING DOCUMENTATION & OPERATIONAL CONSIDERATIONS

12.1. Regulatory, Ethical, and Study Oversight Considerations

12.1.1 Regulatory and Ethical Issues

The study will be conducted in accordance with the ethical principles of the current version of the Declaration of Helsinki. The Sponsor and investigator will comply with their responsibilities as defined in 21 CFR 312.50-312.70, International Conference on Harmonization Guidance for Industry: E6 (R2) Good Clinical Practice (March 2018) and the local regulatory/legal regulations.

The protocol, protocol amendments, ICF, IB, and other relevant documents (e.g., advertisements) must be submitted to an IRB/IEC by the investigator and reviewed and approved by the IRB/IEC before the study is initiated. Any amendments to the protocol will require IRB/IEC approval prior to implementation, except when necessary to eliminate immediate hazard to subjects.

The investigator will be responsible for the following:

- Providing written summaries of the status of the study to the IRB/IEC annually or more frequently in accordance with the requirements, policies, and procedures established by the IRB/IEC
- Notifying the IRB/IEC of SAEs or other significant safety findings as required by IRB/IEC procedures
- Providing oversight of the conduct of the study at the site and adherence to requirements of 21 CFR, International Council for Harmonisation (ICH) guidelines, the IRB/IEC, European regulation 536/2014 for clinical studies (if applicable), and all other applicable local regulations
- While every effort should be made to avoid protocol deviations, should an important deviation be discovered, Sponsor must be informed immediately. Any protocol deviation impacting subject safety must be reported to the Medical Monitor immediately and to the IRB/IEC, as required per institution

12.1.2 Financial Disclosure

Investigators and sub-investigators will provide the sponsor with sufficient, accurate financial information as requested to allow the sponsor to submit complete and accurate financial certification or disclosure

statements to the appropriate regulatory authorities. Investigators are responsible for providing information on financial interests during the course of the study and for 1 year after completion of the study.

12.1.3 Informed Consent Process

The investigator will obtain informed consent from each subject enrolled in the study, in accordance with requirements of 21 CFR 50, local regulations, ICH guidelines, Health Insurance Portability and Accountability Act, Data Protection regulations, where applicable, IRB/IEC, and any local laws and regulations in which the investigation is being conducted.

The IRB must approve the ICF to be used by the investigator. It is the responsibility of the investigator to ensure that informed consent is obtained from the subject or his/her guardian or legal representative before any activity or treatment is undertaken which is not part of routine care. This includes, but is not limited to, the performance of diagnostic, screening, or therapeutic procedures and the administration of the first dose of study treatment. The draft version of the ICF will be modified by each site and reviewed and approved in writing by Geneos Therapeutics prior to submission to the IRB.

The investigator or his/her representative will explain the nature of the study to the subject or his/her legally authorized representative and answer all questions regarding the study. The medical record must include a statement that written informed consent was obtained before the subject was enrolled in the study and the date the written consent was obtained. The authorized person obtaining the informed consent must also sign the ICF. A copy of the ICF(s) must be provided to the subject or the subject's legally authorized representative.

Should a protocol amendment be made, the subject ICF may be revised to reflect the changes of the protocol. If the ICF is revised, it is the responsibility of the investigator to ensure that an amended ICF is reviewed and approved by the IRB and signed by all subjects subsequently, as applicable to their treatment and/or follow-up status in the study.

12.1.4 Data Protection

All local legal requirements regarding data protection will be enforced. All study findings and documents will be regarded as confidential. The investigator and members of his/her research team must not disclose such information without prior written approval from Geneos Therapeutics. The anonymity of participating subjects must be maintained. Throughout documentation and evaluation, the subjects will be identified on CRFs and other documents submitted to Geneos Therapeutics by a unique identifier. Documents that are not to be submitted to Geneos Therapeutics, and that identify the subject (e.g., the signed ICF), must be maintained in confidence by the investigator. The subjects will be told that all study findings will be stored and handled in strictest confidence, according to local requirements. Subjects will be informed that authorized research investigators, IRB/IEC, agents of the FDA or other recognized regulatory authorities, and authorized representatives of the Sponsor, Geneos Therapeutics, have the right to inspect their medical records.

12.1.5 Committee Structure

This is a Phase I/IIa study involving up to 4 study centers located in the United States. The investigator(s) responsible for the conduct of the study at their site, in compliance with this Protocol, are identified on the Signature of Agreement Page (Appendix D).

All questions regarding the enrollment of subjects, regulatory requirements for the conduct of the study, safety reporting, or study conduct should be addressed to the Medical Monitor.

12.1.6 Data Quality Assurance

The following requirements are necessary to ensure quality data from the study:

- All subject data relating to the study will be recorded on electronic case report form (CRF) unless transmitted to the sponsor or designee electronically (e.g., laboratory data). The investigator is responsible for verifying that data entries are accurate and correct by physically or electronically signing the CRF.
- The investigator must maintain accurate documentation (source data) that supports the information entered in the CRF.
- The investigator must permit study-related monitoring, audits, IRB/IEC review, and regulatory agency inspections and provide direct access to source data documents.
- The sponsor or designee is responsible for the data management of this study, including quality checking of the data. Programmed computer edit checks will be run against the database to check for discrepancies and plausibility of the data. All issues resulting from the computer-generated checks will be resolved according to the Sponsor's standard data management practices in conjunction with the medical monitor, clinical study personnel, and the study investigators.
- The sponsor assumes accountability for actions delegated to other individuals (e.g., Contract Research Organizations).
- Study monitors will perform ongoing source data verification to confirm that data entered into the CRF by authorized site personnel are accurate, complete, and verifiable from source documents; that the safety and rights of participants are being protected; and that the study is being conducted in accordance with the currently approved protocol and any other study agreements, ICH Good Clinical Practice, and all applicable regulatory requirements.
- Records and documents, including signed ICFs, pertaining to the conduct of this study must be retained by the investigator for the following timeframes:
    - for a minimum of 2 years following the date the marketing application (New Drug Application/BLA) is approved for the indication for which the drug was investigated; or,
    - for a minimum of 2 years following the release date of the final report, if no marketing application is to be filed by Geneos Therapeutics, or if the marketing application is not approved for the indication for which the drug was investigated or is discontinued, and the FDA has been notified; or,
    - for a minimum of 15 years after the completion or discontinuation of the study to be filed in support of the registration in the European Union.
- No records may be destroyed without the written approval of the sponsor. No records may be transferred to another location or party without written notification to the sponsor.

12.1.7 Source Documents

Source documents provide evidence for the existence of the subject and substantiate the integrity of the data collected. Source documents are filed at the investigator’s site.

Data reported on the CRF or entered in the eCRF that are transcribed from source documents must be consistent with the source documents or the discrepancies must be explained. The investigator may need to

request previous medical records or transfer records, depending on the study. Also, current medical records must be available.

12.1.8 Study and Site Closure

The sponsor designee reserves the right to close the study site or terminate the study at any time for any reason at the sole discretion of the sponsor. Study sites will be closed upon study completion. A study site is considered closed when all required documents and study supplies have been collected and a study-site closure visit has been performed.

The investigator may initiate study-site closure at any time, provided there is reasonable cause and sufficient notice is given in advance of the intended termination.

Reasons for the early closure of a study site by the sponsor or investigator may include but are not limited to:

- Failure of the investigator to comply with the protocol, the requirements of the IRB/IEC or local health authorities, the sponsor's procedures, or ICH Good Clinical Practice guidelines
- Inadequate recruitment of subjects by the investigator
- Discontinuation of further study intervention development

Upon terminating the study, the Investigator will submit a final report to the IRB in keeping with local IRB regulations. This report should include any deviations from the protocol, the number and types of subjects evaluated, the number of subjects who discontinued, including reasons, results of the study, AEs, and a conclusion summarizing the results.

If requested by the Investigator, at the completion of the study and following analysis of the data, Geneos Therapeutics will supply a tabulated listing of data and a final clinical statistical report. A copy of the final study report and final CRFs, will be provided to each Investigator.

12.1.9 Publication Policy

By signing the study protocol, the Investigator agrees with the use of results of the study for the purposes of national and international registration, publication, and information for medical and pharmaceutical professionals. If necessary, the authorities will be notified of the investigator's name, address, qualifications, and extent of involvement. The results of the study may appear on public websites, such as www.clinicaltrials.gov, in compliance with clinical study reporting requirements. Geneos Therapeutics will prepare an Integrated Clinical/Statistical Report. Any publication/presentation of data must include the entire study population. Submission of data for publication/presentation will be reviewed, coordinated, and approved by Geneos Therapeutics in collaboration with the Investigator. Geneos Therapeutics will determine authorship of any publication by enrollment or by contributing to the protocol, in consultation with the principal Investigator. If the Investigator wishes to publish on his/her own, written permission from Geneos Therapeutics must be granted in advance.

13. REFERENCE LIST

1. Ferlay J, Colombet M, Soerjomataram I, Mathers C, Parkin DM, Pineros M, et al. Estimating the global cancer incidence and mortality in 2018: GLOBOCAN sources and methods. Int J Cancer. 2019;144(8):1941-53.
2. Llovet JM, Ricci S, Mazzaferro V, Hilgard P, Gane E, Blanc JF, et al. Sorafenib in advanced hepatocellular carcinoma. N Engl J Med. 2008;359(4):378-90.

3. Cheng AL, Kang YK, Chen Z, Tsao CJ, Qin S, Kim JS, et al. Efficacy and safety of sorafenib in patients in the Asia-Pacific region with advanced hepatocellular carcinoma: a phase III randomised, double-blind, placebo-controlled trial. Lancet Oncol. 2009;10(1):25-34.
4. Zhu AX, Finn RS, Edeline J, Cattan S, Ogasawara S, Palmer D, et al. Pembrolizumab in patients with advanced hepatocellular carcinoma previously treated with sorafenib (KEYNOTE-224): a non-randomised, open-label phase 2 trial. Lancet Oncol. 2018;19(7):940-52.
5. Kudo M. Pembrolizumab for the Treatment of Hepatocellular Carcinoma. Liver Cancer. 2019;8(3):143-54.
6. Kudo M. Extremely High Objective Response Rate of Lenvatinib: Its Clinical Relevance and Changing the Treatment Paradigm in Hepatocellular Carcinoma. Liver Cancer. 2018;7(3):215-24.
7. Parmiani G, De Filippo A, Novellino L, Castelli C. Unique human tumor antigens: immunobiology and use in clinical trials. J Immunol. 2007;178(4):1975-9.
8. Wolfel T, Hauer M, Schneider J, Serrano M, Wolfel C, Klehmann-Hieb E, et al. A p16INK4a-insensitive CDK4 mutant targeted by cytolytic T lymphocytes in a human melanoma. Science. 1995;269(5228):1281-4.
9. Matsushita H, Vesely MD, Koboldt DC, Rickert CG, Uppaluri R, Magrini VJ, et al. Cancer exome analysis reveals a T-cell-dependent mechanism of cancer immunoediting. Nature. 2012;482(7385):400-4.
10. Ulmer JB, Donnelly JJ, Parker SE, Rhodes GH, Felgner PL, Dwarki VJ, et al. Heterologous protection against influenza by injection of DNA encoding a viral protein. Science. 1993;259(5102):1745-9.
11. Donnelly JJ, Ulmer JB, Shiver JW, Liu MA. DNA vaccines. Annu Rev Immunol. 1997;15:617-48.
12. Gurunathan S, Klinman DM, Seder RA. DNA vaccines: immunology, application, and optimization*. Annu Rev Immunol. 2000;18:927-74.
13. Duperret EK, Perales-Puchalt A, Stoltz R, G HH, Mandloi N, Barlow J, et al. A Synthetic DNA, Multi-Neoantigen Vaccine Drives Predominately MHC Class I CD8(+) T-cell Responses, Impacting Tumor Challenge. Cancer Immunol Res. 2019;7(2):174-82.
14. Chattergoon MA, Saulino V, Shames JP, Stein J, Montaner LJ, Weiner DB. Co-immunization with plasmid IL-12 generates a strong T-cell memory response in mice. Vaccine. 2004;22(13-14):1744-50.
15. Kim JJ, Ayyavoo V, Bagarazzi ML, Chattergoon MA, Dang K, Wang B, et al. In vivo engineering of a cellular immune response by coadministration of IL-12 expression vector with a DNA immunogen. Journal of Immunology. 1997;158(2):816-26.
16. Kim JJ, Maguire HC, Jr., Nottingham LK, Morrison LD, Tsai A, Sin JI, et al. Coadministration of IL-12 or IL-10 expression cassettes drives immune responses toward a Th1 phenotype. JInterferon Cytokine Res. 1998;18(7):537-47.
17. Kim JJ, Trivedi NN, Nottingham LK, Morrison L, Tsai A, Hu Y, et al. Modulation of amplitude and direction of in vivo immune responses by co-administration of cytokine gene expression cassettes with DNA immunogens. EurJImmunol. 1998;28(3):1089-103.
18. Sartori A, Ma X, Gri G, Showe L, Benjamin D, Trinchieri G. Interleukin-12: an immunoregulatory cytokine produced by B cells and antigen-presenting cells. Methods. 1997;11(1):116-27.
19. Boyer JD, Cohen AD, Ugen KE, Edgeworth RL, Bennett M, Shah A, et al. Therapeutic immunization of HIV-infected chimpanzees using HIV-1 plasmid antigens and interleukin-12 expressing plasmids. AIDS. 2000;14(11):1515-22.
20. Boyer JD, Robinson TM, Kutzler MA, Parkinson R, Calarota SA, Sidhu MK, et al. SIV DNA vaccine co-administered with IL-12 expression plasmid enhances CD8 SIV cellular immune responses in cynomolgus macaques. JMedPrimatol. 2005;34(5-6):262-70.

21. Schadeck EB, Sidhu M, Egan MA, Chong SY, Piacente P, Masood A, et al. A dose sparing effect by plasmid encoded IL-12 adjuvant on a SIVgag-plasmid DNA vaccine in rhesus macaques. Vaccine. 2006;24(21):4677-87.
22. Chong SY, Egan MA, Kutzler MA, Megati S, Masood A, Roopchard V, et al. Comparative ability of plasmid IL-12 and IL-15 to enhance cellular and humoral immune responses elicited by a SIVgag plasmid DNA vaccine and alter disease progression following SHIV(89.6P) challenge in rhesus macaques. Vaccine. 2007;25(26):4967-82.
23. Kalams SA, Parker S, Jin X, Elizaga M, Metch B, Wang M, et al. Safety and immunogenicity of an HIV-1 gag DNA vaccine with or without IL-12 and/or IL-15 plasmid cytokine adjuvant in healthy, HIV-1 uninfected adults. PLoSONE. 2012;7(1):e29231.
24. Kalams SA, Parker SD, Elizaga M, Metch B, Edupuganti S, Hural J, et al. Safety and comparative immunogenicity of an HIV-1 DNA vaccine in combination with plasmid interleukin 12 and impact of intramuscular electroporation for delivery. The Journal of infectious diseases. 2013;208(5):818-29.
25. Kutzler MA, Weiner DB. DNA vaccines: ready for prime time? Nat Rev Genet. 2008;9(10):776-88.
26. Aihara H, Miyazaki J. Gene transfer into muscle by electroporation in vivo. NatBiotechnol. 1998;16(9):867-70.
27. Draghia-Akli R, Khan AS, Brown PA, Pope MA, Wu L, Hirao L, et al. Parameters for DNA vaccination using adaptive constant-current electroporation in mouse and pig models. Vaccine. 2008;26(40):5230-7.
28. Hirao LA, Wu L, Khan AS, Satishchandran A, Draghia-Akli R, Weiner DB. Intradermal/subcutaneous immunization by electroporation improves plasmid vaccine delivery and potency in pigs and rhesus macaques. Vaccine. 2008;26(3):440-8.
29. Schoenly KA, Weiner DB. Human immunodeficiency virus type 1 vaccine development: recent advances in the cytotoxic T-lymphocyte platform "spotty business". Journal of virology. 2008;82(7):3166-80.
30. Liu J, Kjeken R, Mathiesen I, Barouch DH. Recruitment of antigen-presenting cells to the site of inoculation and augmentation of human immunodeficiency virus type 1 DNA vaccine immunogenicity by in vivo electroporation. J Virol. 2008;82(11):5643-9.
31. Luckay A, Sidhu MK, Kjeken R, Megati S, Chong SY, Roopchand V, et al. Effect of plasmid DNA vaccine design and in vivo electroporation on the resulting vaccine-specific immune responses in rhesus macaques. JVirol. 2007;81(10):5257-69.
32. Bagarazzi ML, Yan J, Morrow MP, Shen X, Parker RL, Lee JC, et al. Immunotherapy against HPV16/18 generates potent TH1 and cytotoxic cellular immune responses. SciTranslMed. 2012;4(155):155ra38.
33. Favard C, Dean DS, Rols MP. Electrotransfer as a non viral method of gene delivery. CurrGene Ther. 2007;7(1):67-77.
34. Gothelf A, Mir LM, Gehl J. Electrochemotherapy: results of cancer treatment using enhanced delivery of bleomycin by electroporation. Cancer TreatRev. 2003;29(5):371-87.
35. Frederickson RM, Carter BJ, Pilaro AM. Nonclinical toxicology in support of licensure of gene therapies. Arlington, VA, USA, March 13-14, 2003. Molecular therapy : the journal of the American Society of Gene Therapy. 2003;8(1):8-10.
36. Sheets RL, Stein J, Manetz TS, Andrews C, Bailer R, Rathmann J, et al. Toxicological safety evaluation of DNA plasmid vaccines against HIV-1, Ebola, Severe Acute Respiratory Syndrome, or West Nile virus is similar despite differing plasmid backbones or gene-inserts. ToxicolSci. 2006;91(2):620-30.

37. Sheets RL, Stein J, Manetz TS, Duffy C, Nason M, Andrews C, et al. Biodistribution of DNA plasmid vaccines against HIV-1, Ebola, Severe Acute Respiratory Syndrome, or West Nile virus is similar, without integration, despite differing plasmid backbones or gene inserts. ToxicolSci. 2006;91(2):610-9.
38. Sheets RL, Stein J, Bailer RT, Koup RA, Andrews C, Nason M, et al. Biodistribution and toxicological safety of adenovirus type 5 and type 35 vectored vaccines against human immunodeficiency virus-1 (HIV-1), Ebola, or Marburg are similar despite differing adenovirus serotype vector, manufacturer's construct, or gene inserts. JImmunotoxicol. 2008;5(3):315-35.
39. Dolter KE, Evans CF, Ellefsen B, Song J, Boente-Carrera M, Vittorino R, et al. Immunogenicity, safety, biodistribution and persistence of ADVAX, a prophylactic DNA vaccine for HIV-1, delivered by in vivo electroporation. Vaccine. 2011;29(4):795-803.
40. Ondondo B, Brennan C, Nicosia A, Crome SJ, Hanke T. Absence of systemic toxicity changes following intramuscular administration of novel pSG2.HIVconsv DNA, ChAdV63.HIVconsv and MVA.HIVconsv vaccines to BALB/c mice. Vaccine. 2013(13):10.
41. Plog MS, Guyre CA, Roberts BL, Goldberg M, St George JA, Perricone MA. Preclinical safety and biodistribution of adenovirus-based cancer vaccines after intradermal delivery. HumGene Ther. 2006;17(7):705-16.
42. Peng JL, Zhao YG, Mai JH, Pang WK, Guo W, Chen GM, et al. Non-cytolytic antigen clearance in DNA-vaccinated mice with electroporation. Acta PharmacolSin. 2007;28(7):1024-30.
43. Pilling AM, Harman RM, Jones SA, McCormack NA, Lavender D, Haworth R. The assessment of local tolerance, acute toxicity, and DNA biodistribution following particle-mediated delivery of a DNA vaccine to minipigs. ToxicolPathol. 2002;30(3):298-305.
44. Ledgerwood JE, Pierson TC, Hubka SA, Desai N, Rucker S, Gordon IJ, et al. A West Nile virus DNA vaccine utilizing a modified promoter induces neutralizing antibody in younger and older healthy adults in a phase I clinical trial. JInfectDis. 2011;203(10):1396-404.
45. Martin JE, Louder MK, Holman LA, Gordon IJ, Enama ME, Larkin BD, et al. A SARS DNA vaccine induces neutralizing antibody and cellular immune responses in healthy adults in a Phase I clinical trial. Vaccine. 2008;26(50):6338-43.
46. Tebas P, Kraynyak KA, Patel A, Maslow JN, Morrow MP, Sylvester AJ, et al. Intradermal SynCon(R) Ebola GP DNA Vaccine is Temperature Stable and Safely Demonstrates Cellular and Humoral Immunogenicity Advantages in Healthy Volunteers. The Journal of infectious diseases. 2019.
47. Tebas P, Roberts CC, Muthumani K, Reuschel EL, Kudchodkar SB, Zaidi FI, et al. Safety and Immunogenicity of an Anti-Zika Virus DNA Vaccine - Preliminary Report. N Engl J Med. 2017.
48. Nishino M. Immune-related response evaluations during immune-checkpoint inhibitor therapy: establishing a "common language" for the new arena of cancer treatment. J Immunother Cancer. 2016;4:30.
49. El-Khoueiry AB, Sangro B, Yau T, Crocenzi TS, Kudo M, Hsu C, et al. Nivolumab in patients with advanced hepatocellular carcinoma (CheckMate 040): an open-label, non-comparative, phase 1/2 dose escalation and expansion trial. Lancet. 2017;389(10088):2492-502.

APPENDIX A: PREEXISTING AUTOIMMUNE DISEASE

Patients should be carefully questioned regarding their history of acquired or congenital immune deficiencies or autoimmune disease. Patients with any history of immune deficiencies or autoimmune disease listed in the table below are excluded from participating in the study. Possible exceptions to this exclusion could be patients with a medical history of such entities as atopic disease or childhood arthralgias where the clinical suspicion of autoimmune disease is low. Patients with a history of autoimmune-related hypothyroidism on a stable dose of thyroid replacement hormone may be eligible for this study. In addition, transient autoimmune manifestations of an acute infectious disease that resolved upon treatment of the infectious agent are not excluded (e.g., acute Lyme arthritis). Please contact the Sponsor regarding any uncertainty over autoimmune exclusions.

Acute disseminated encephalomyelitis	Interstitial cystitis
Addison's disease	Kawasaki's disease
Ankylosing spondylitis	Lambert-Eaton myasthenia syndrome
Antiphospholipid antibody syndrome	Lupus erythematosus
Aplastic anemia	Lyme disease – chronic
Autoimmune hemolytic anemia	Mooren's ulcer
Autoimmune hepatitis	Morphea
Autoimmune hypoparathyroidism	Multiple sclerosis
Autoimmune myocarditis	Myasthenia gravis
Autoimmune oophoritis	Neuromyotonia
Autoimmune orchitis	Opsoclonus myoclonus syndrome
Autoimmune thrombocytopenic purpura	Optic neuritis
Behcet's disease	Ord's thyroiditis
Bullous pemphigold	Pemphigus
Chronic inflammatory demyelinating polyneuropathy	Pernicious anemia
Chung-Strauss syndrome	Polyarteritis nodosa
Crohn's disease	Polyarthritis
Dermatomyositis	Polyglandular autoimmune syndrome
Diabetes mellitus Type I	Primary biliary cirrhosis
Dysautonomia	Psoriasis
Epidermolysis bullosa acquisita	Reiter's syndrome
Gestational pemphigoid	Rheumatoid arthritis
Giant cell arteritis	Sarcoidosis
Goodpasture's syndrome	Scleroderma
Granulomatosis with polyangiitis	Sjögren's syndrome
Grave's disease	Takayasu's arteritis
Guillain-Barré syndrome	Ulcerative colitis
Hashimoto's disease	Vogt-Kovanagi-Harada disease
IgA nephropathy	
Inflammatory bowel disease	

APPENDIX B: TUMOR ASSESSMENT CRITERIA

RECIST 1.1

Definitions as per Eisenhauer EA, Therasse P, Bogaerts J, et al. New response evaluation criteria in solid tumors: Revised RECIST guideline (Version 1.1). Eur J Cancer 2009; 45:228–47.

At baseline, tumor lesions/lymph nodes will be categorized measurable or non-measurable as follows:

Measurable Tumor Lesions

Tumor lesions must be accurately measured in at least one dimension

(longest diameter in the plane of measurement is to be recorded) with a minimum size of:

- 10 mm by CT or MRI scan (CT/MRI scan slice thickness/interval no greater than 5 mm)
- 10-mm caliper measurement by clinical examination (lesions that cannot be accurately measured with calipers should be recorded as non-measurable)
- 20 mm by chest X-ray

Malignant Lymph Nodes

To be considered pathologically enlarged and measurable, a lymph node must be ≥15 mm in the short axis when assessed by CT scan (CT scan slice thickness recommended to be no greater than 5 mm). At baseline and in follow-up, only the short axis will be measured and followed. See also notes below on "Baseline Documentation of Target and Nontarget Lesions" for information on lymph node measurement.

Non-Measurable Tumor Lesions

Non-measurable tumor lesions encompass small lesions (longest diameter <10 mm or pathological lymph nodes with ≥10 to <15 mm short axis), as well as truly non-measurable lesions. Lesions considered truly non-measurable include leptomeningeal disease, ascites, pleural or pericardial effusion, inflammatory breast disease, lymphangitic involvement of skin or lung, peritoneal spread, and abdominal masses/abdominal organomegaly identified by physical examination that is not measurable by reproducible imaging techniques.

Special Considerations Regarding Lesion Measurability

Bone lesions, cystic lesions, and lesions previously treated with local therapy require particular comment, as outlined below.

Bone lesions:

- Bone scan, positron emission tomography (PET) scan, or plain films are not considered adequate imaging techniques to measure bone lesions. However, these techniques can be used to confirm the presence or disappearance of bone lesions.
- Lytic bone lesions or mixed lytic-blastic lesions, with identifiable soft tissue components, that can be evaluated by cross-sectional imaging techniques such as CT or MRI can be considered measurable lesions if the soft tissue component meets the definition of measurability described above.
- Blastic bone lesions are non-measurable.

Cystic lesions:

- Lesions that meet the criteria for radiographically defined simple cysts should not be considered malignant lesions (neither measurable nor non-measurable) since they are, by definition, simple cysts.
- Cystic lesions thought to represent cystic metastases can be considered measurable lesions if they meet the definition of measurability described above. However, if noncystic lesions are present in the same patient, these are preferred for selection as target lesions.

Lesions with prior local treatment:

- Tumor lesions situated in a previously irradiated area, or in an area subjected to other loco-regional therapy, are usually not considered measurable unless there has been demonstrated progression in the lesion. Study protocols should detail the conditions under which such lesions would be considered measurable.

SPECIFICATIONS BY METHODS OF MEASUREMENTS

Measurement of Lesions

All measurements should be recorded in metric notation, using calipers if clinically assessed. All baseline evaluations should be performed as close as possible to the treatment start and never more than 4 weeks before the beginning of the treatment.

Method of Assessment

The same method of assessment and the same technique should be used to characterize each identified and reported lesion at baseline and during study. Imaging-based evaluation should always be done rather than clinical examination unless the lesion(s) being followed cannot be imaged but are assessable by clinical exam.

Clinical Lesions

Clinical lesions will only be considered measurable when they are superficial and ≥10 mm in diameter as assessed using calipers (e.g., skin nodules). For the case of skin lesions, documentation by color photography, including a ruler to estimate the size of the lesion, is suggested.

Chest X-Ray

Chest CT is preferred over chest X-ray, particularly when progression is an important endpoint, since CT is more sensitive than X-ray, particularly in identifying new lesions. However, lesions on chest X-ray may be considered measurable if they are clearly defined and surrounded by aerated lung.

CT, MRI

CT is the best currently available and reproducible method to measure lesions selected for response assessment. This guideline has defined measurability of lesions on CT scan based on the assumption that CT slice thickness is 5 mm or less. When CT scans have slice thickness greater than 5 mm, the minimum size for a measurable lesion should be twice the slice thickness. MRI is also acceptable.

Ultrasound

Ultrasound is not useful in the assessment of lesion size and should not be used as a method of measurement.

Endoscopy, Laparoscopy, Tumor Markers, Cytology, Histology

The utilization of these techniques for objective tumor evaluation is not advised.

TUMOR RESPONSE EVALUATION

Assessment of Overall Tumor Burden and Measurable Disease

To assess objective response or future progression, it is necessary to estimate the overall tumor burden at baseline and to use this as a comparator for subsequent measurements. Measurable disease is defined by the presence of at least one measurable lesion.

Baseline Documentation of Target and Nontarget Lesions

When more than one measurable lesion is present at baseline, all lesions up to a maximum of 5 lesions total (and a maximum of 2 lesions per organ) representative of all involved organs should be identified as target lesions and will be recorded and measured at baseline (this means in instances where patients have only 1 or 2 organ sites involved, a maximum of 2 and 4 lesions, respectively, will be recorded).

Target lesions should be selected on the basis of their size (lesions with the longest diameter) and be representative of all involved organs, but in addition should be those that lend themselves to reproducible repeated measurements. It may be the case that, on occasion, the largest lesion does not lend itself to reproducible measurement, in which circumstance the next largest lesion that can be measured reproducibly should be selected.

Lymph nodes merit special mention since they are normal anatomical structures that may be visible by imaging even if not involved by tumor. As noted above, pathological nodes that are defined as measurable and may be identified as target lesions must meet the criterion of a short axis of ≥15 mm by CT scan. Only the short axis of these nodes will contribute to the baseline sum. The short axis of the node is the diameter normally used by radiologists to judge if a node is involved by solid tumor. Nodal size is normally reported as 2 dimensions in the plane in which the image is obtained (for CT scan, this is almost always the axial plane; for MRI the plane of acquisition may be axial, sagittal, or coronal). The smaller of these measures is the short axis. For example, an abdominal node that is reported as being 20 mm x 30 mm has a short axis of 20 mm and qualifies as a malignant, measurable node. In this example, 20 mm should be recorded as the node measurement. All other pathological nodes (those with short axis ≥10 mm but <15 mm) should be considered nontarget lesions. Nodes that have a short axis <10 mm are considered nonpathological and should not be recorded or followed.

A sum of the diameters (longest for non-nodal lesions, short axis for nodal lesions) for all target lesions will be calculated and reported as the baseline sum of diameters. If lymph nodes are to be included in the sum, then, as noted above, only the short axis is added into the sum. The baseline sum of diameters will be used as a reference to further characterize any objective tumor regression in the measurable dimension of the disease.

All other lesions (or sites of disease), including pathological lymph nodes, should be identified as nontarget lesions and should also be recorded at baseline. Measurements are not required, and these lesions should be followed as “present,” “absent,” or in rare cases “unequivocal progression.”

In addition, it is possible to record multiple nontarget lesions involving the same organ as a single item on the Case Report Form (CRF) (e.g., “multiple enlarged pelvic lymph nodes” or “multiple liver metastases”).

RESPONSE CRITERIA

Evaluation of Target Lesions

This section provides the definitions of the criteria used to determine objective tumor response for target lesions.

- **Complete response (CR)**: Disappearance of all target lesions. Any pathological lymph nodes (whether target or nontarget) must have reduction in short axis to <10 mm.
- **Partial response (PR)**: At least a 30% decrease in the sum of diameters of target lesions, taking as reference the baseline sum of diameters
- **Progressive disease (PD)**: At least a 20% increase in the sum of diameters of the target lesions, taking as reference the smallest sum on study (nadir), including baseline. In addition to the relative increase of 20%, the sum must also demonstrate an absolute increase of at least 5 mm. Note: the appearance of one or more new lesions is also considered progression.
- **Stable disease (SD)**: Neither sufficient shrinkage to qualify for PR nor sufficient increase to qualify for PD, taking as reference the smallest sum on study

Special Notes on the Assessment of Target Lesions

Lymph Nodes. Lymph nodes identified as target lesions should always have the actual short axis measurement recorded (measured in the same anatomical plane as the baseline examination), even if the nodes regress to below10 mm on study. This means that when lymph nodes are included as target lesions, the sum of lesions may not be zero even if CR criteria are met since a normal lymph node is defined as having a short axis <10 mm.

Target Lesions That Become Too Small to Measure

While on study, all lesions (nodal and non-nodal) recorded at baseline should have their actual measurements recorded at each subsequent evaluation, even when very small (e.g., 2 mm). However, sometimes lesions or lymph nodes that are recorded as target lesions at baseline become so faint on the CT scan that the radiologist may not feel comfortable assigning an exact measure and may report them as being too small to measure. When this occurs, it is important that a value be recorded on the CRF as follows:

- If it is the opinion of the radiologist that the lesion has likely disappeared, the measurement should be recorded as 0 mm.
- If the lesion is believed to be present and is faintly seen but too small to measure, a default value of 5 mm should be assigned and below measurable limit (BML) should be ticked. (Note: It is less likely that this rule will be used for lymph nodes since they usually have a definable size when normal and are frequently surrounded by fat such as in the retroperitoneum; however, if a lymph node is believed to be present and is faintly seen but too small to measure, a default value of 5 mm should be assigned in this circumstance as well). To reiterate, however, if the radiologist is able to provide an actual measure, that should be recorded, even if it is <5 mm.

Lesions That Split or Coalesce on Treatment

When non-nodal lesions fragment, the longest diameters of the fragmented portions should be added together to calculate the target lesion sum. Similarly, as lesions coalesce, a plane between them may be maintained that would aid in obtaining maximal diameter measurements of each individual lesion. If the lesions have truly coalesced such that they are no longer separable, the vector of the longest diameter in this instance should be the maximal longest diameter for the coalesced lesion.

Evaluation of Nontarget Lesions

This section provides the definitions of the criteria used to determine the tumor response for the group of nontarget lesions. While some nontarget lesions may actually be measurable, they need not be measured and, instead, should be assessed only qualitatively at the time points specified in the protocol.

- **CR**: Disappearance of all nontarget lesions and normalization of tumor marker level. All lymph nodes must be non-pathological in size (<10 mm short axis).
- **Non-CR/Non-PD**: Persistence of one or more nontarget lesion(s) and/or maintenance of tumor marker level above the normal limits.
- **PD**: Unequivocal progression of existing nontarget lesions. The appearance of one or more new lesions is also considered progression.

Special Notes on Assessment of Progression of Nontarget Disease When the Patient Also Has Measurable Disease

In this setting, to achieve "unequivocal progression" on the basis of the nontarget disease, there must be an overall level of substantial worsening in nontarget disease in a magnitude that, even in the presence of SD or PR in target disease, the overall tumor burden has increased sufficiently to merit discontinuation of therapy. A modest increase in the size of one or more nontarget lesions is usually not sufficient to qualify for unequivocal progression status. The designation of overall progression solely on the basis of change in nontarget disease in the face of SD or PR of target disease will therefore be extremely rare.

When the Patient Has Only Non-Measurable Disease

This circumstance arises in some phase III studies when it is not a criterion of study entry to have measurable disease. The same general concepts apply here as noted above; however, in this instance, there is no measurable disease assessment to factor into the interpretation of an increase in non-measurable disease burden. Because worsening in nontarget disease cannot be easily quantified (by definition: if all lesions are truly non-measurable), a useful test that can be applied when assessing patients for unequivocal progression is to consider if the increase in overall disease burden based on the change in non-measurable disease is comparable in magnitude to the increase that would be required to declare PD for measurable disease, that is, an increase in tumor burden representing an additional 73% increase in volume (which is equivalent to a 20% increase in diameter in a measurable lesion). Examples include an increase in a pleural effusion from "trace" to "large," an increase in lymphangitic disease from localized to widespread or may be described in protocols as "sufficient to require a change in therapy." If unequivocal progression is seen, the patient should be considered to have had overall PD at that point. While it would be ideal to have objective criteria to apply to non-measurable disease, the very nature of that disease makes it impossible to do so; therefore, the increase must be substantial.

New Lesions

The appearance of new malignant lesions denotes disease progression; therefore, some comments on detection of new lesions are important. There are no specific criteria for the identification of new radiographic lesions; however, the finding of a new lesion should be unequivocal, i.e., not attributable to differences in scanning technique, change in imaging modality, or findings thought to represent something other than tumor (for example, some "new" bone lesions may be simply healing or flare of preexisting lesions). This is particularly important when the patient's baseline lesions show partial or complete response. For example, necrosis of a liver lesion may be reported on a CT scan report as a "new" cystic lesion, which it is not.

A lesion identified on a follow-up study in an anatomical location that was not scanned at baseline is considered a new lesion and will indicate disease progression.

If a new lesion is equivocal, for example, because of its small size, continued therapy and follow-up evaluation will clarify if it represents truly new disease. If repeat scans confirm there is definitely a new lesion, then progression should be declared using the date of the initial scan.

EVALUATION OF RESPONSE

Table 11 provides a summary of the overall response status calculation at each timepoint for patients who have measurable disease at baseline.

Table 12 provides a summary of non-measurable (therefore nontarget) disease only

Table 13 provides the BOR when confirmation of CR and PR required

Table 11. Time Point Response: Patients with Target (± Non-target Disease)

Target Lesions	Non-Target Lesions	New Lesions	Overall Response
CR	CR	No	CR
CR	Non-CR/Non-PD	No	PR
CR	Not evaluated	No	PR
PR	Non-PD or not all evaluated	No	PR
SD	Non-PD or not all evaluated	No	SD
Not all evaluated	Non-PD	No	NE
PD	Any	Yes or No	PD
Any	PD	Yes or No	PD
Any	Any	Yes	PD

Table 12. Time Point Response: Patients with Non-target Disease Only

Non-target lesions	New lesions	Overall response
CR	No	CR
Non-CR/non-PD	No	Non-CR/non-PDa
Not all evaluated	No	NE
Unequivocal PD	Yes or No	PD
Any	Yes	PD

CR = complete response; PD = progressive disease; NE = not evaluable.

[a] Non-CR/non-PD is preferred over stable disease for non-target disease since SD is increasingly used as endpoint for assessment of efficacy in some studies so to assign this category when no lesions can be measured is not advised.

Table 13. Best Overall Response when Confirmation of Complete Response and Partial Response required

Overall response<br>First time point	Overall response<br>Subsequent time point	Best overall response
CR	CR	CR
CR	PR	SD, PD or PR[a]
CR	SD	SD provided minimum criteria for SD duration met, otherwise PD
CR	PD	SD provided minimum criteria for SD duration met, otherwise PD
CR	NE	SD provided minimum criteria for SD duration met, otherwise NE
PR	CR	PR
PR	PR	PR
PR	SD	SD
PR	PD	SD provided minimum criteria for SD duration met, otherwise PD
PR	NE	SD provided minimum criteria for SD duration met, otherwise NE
NE	NE	NE

CR = complete response; PR = partial response; SD = stable disease; PD = progressive disease; NE = not evaluable

[a] If a CR is truly met at first time point, then any disease seen at a subsequent time point, even disease meeting PR criteria relative to baseline, makes the disease PD at that point (since disease must have reappeared after CR). Best response would depend on whether minimum duration for SD was met. However, sometimes CR may be claimed when subsequent scans suggest small lesions were likely still present and in fact the patient had PR, not CR, at the first time point. Under these circumstances, the original CR should be changed to PR and the best response is PR.

iRECIST

Definitions as per Seymour, L, et al. iRECIST: guidelines for response criteria for use in trials testing immunotherapeutics. Lancet 2017; PE143-E152.

iRECIST Response Assessment

Overall response will also be assessed using iRECIST. Immunotherapeutics may result in infiltration of immune cells leading to transient increase in the size in malignant lesions, or undetectable lesions becoming detectable. The criteria are identical to those of RECIST 1.1 in many respects but have been adapted to account for instances where an increase in tumor burden, or the appearance of new lesions, does not reflect true tumor progression.

Key differences are described below. All responses defined using iRECIST criteria are designated with a prefix. iRECIST time point and BORs will be recorded separately.

Confirming Disease Progression

Unlike RECIST 1.1, iRECIST requires the confirmation of progression and uses the terms iUPD (unconfirmed progression) and iCPD (confirmed progression). Confirmatory scans should be performed at least 4 weeks, but no longer than 6weeks after iUPD.

iCPD is confirmed if further increase in tumor burden, compared to the last assessment, is seen as evidenced by one or more of the following:

- Continued increase in tumor burden (from iUPD) where RECIST 1.1 definitions of progression had been met (from nadir) in target, non-target disease or new lesions
- Progression in target disease worsens with an increase of at least 5 mm in the absolute value of the sum
- Continued unequivocal progression in non-target disease with an increase in tumor burden
- Increase in size of previously identified new lesion (s) (an increase of at least 5 mm in the absolute value of the sum of those considered to be target new lesions) or additional new lesions
- RECIST 1.1 criteria are met in lesions types (target or non-target or new lesions) where progression was not previously identified, including the appearance of additional new lesions

If iUPD is not confirmed at the next assessment, then the appropriate response will be assigned (iUPD if the criteria are still met, but no worsening, or iSD, iPR or iCR if those criteria are met compared to baseline). As can be seen in Table 12, the prior documentation of iUPD does not preclude assigning iCR, iPR, or iSD in subsequent time-point assessments or as BOR providing that iCPD is not documented at the next assessment after iUPD.

New lesions

New lesions should be assessed and measured as they appear using RECIST 1.1 criteria (maximum of 5 lesions, no more than 2 per site, at least 10 mm in long axis (or 15 mm in short axis for nodal lesions) and recorded as New Lesions-Target (NLT) and New Lesion-Non-Target (NLNT) to allow clear differentiation from baseline target and non-target lesions.

New lesions may either meet the criteria of NLT or NLNT to drive iUPD (or iCPD). However, the measurements of target lesions should NOT be included in the sum of measures of original target lesions identified at baseline. Rather, these measurements will be collected on a separate table in the case record form.

PD is confirmed in the New Lesion category if the next imaging assessment, conducted at least 4 weeks (but not more than 8 weeks) after iUPD confirms further progression from iUPD with either an increase of at least 5 mm in the absolute value of the sum of NLT or an increase (but not necessarily unequivocal increase) in the size of NLNT lesions OR the appearance of additional new lesions

Response and Stable Disease Duration (RECIST 1.1 and iRECIST)

Response duration will be measured from the time measurement criteria for CR/PR or iCR/iPR (whichever is first recorded) are first met until the first date that recurrent or progressive disease is objectively documented, taking as reference the smallest measurements recorded on study (including baseline).

Stable disease duration will be measured from the time of start of treatment until the criteria for progression are met, taking as reference the smallest sum on study (including baseline).

Methods of Measurement

The same method of assessment and the same technique should be used to characterize each identified and reported lesion at baseline and during follow-up. Assessments should be identified on a calendar schedule and should not be affected by delays in therapy. While on study, all lesions recorded at baseline should have their actual measurements recorded at each subsequent evaluation, even when very small (e.g. 2 mm). If it is the opinion of the radiologist that the lesion has likely disappeared, the measurement should be recorded as 0 mm. If the lesion is believed to be present and is faintly seen but too small to measure, a default value of 5 mm should be assigned. For lesions which fragment/split, add together the longest diameters of the fragmented portions; for lesions which coalesce, measure the maximal longest diameter for the "merged lesion".

Clinical Lesions: Clinical lesions will only be considered measurable when they are superficial and ≥10 mm as assessed using calipers (e.g. skin nodules). For the case of skin lesions, documentation by color photography including a ruler to estimate the size of the lesion is recommended. If feasible, imaging is preferred.

Chest X-ray: Chest CT is preferred over chest X-ray, particularly when progression is an important endpoint, since CT is more sensitive than X-ray, particularly in identifying new lesions. However, lesions ≥20 mm on chest X-ray may be considered measurable if they are clearly defined and surrounded by aerated lung.

CT, MRI: CT is the best currently available and reproducible method to measure lesions selected for response assessment. This guideline has defined measurability of lesions on CT scan based on the assumption that CT slice thickness is 5 mm or less. When CT scans have slice thickness greater than 5 mm, the minimum size for a measurable lesion should be twice the slice thickness. MRI is also acceptable in certain situations (e.g. for body scans). Other specialized imaging or other techniques may also be appropriate for individual case. For example, while PET scans are not considered adequate to measure lesions, PET-CT scans may be used providing that the measures are obtained from the CT scan and the CT scan is of identical diagnostic quality to a diagnostic CT (with IV and oral contrast).

Ultrasound: Ultrasound is not useful in assessment of lesion size and should not be used as a method of measurement. If new lesions are identified by ultrasound in the course of the study, confirmation by CT is advised.

Endoscopy, Laparoscopy: The utilization of these techniques for objective tumor evaluation is not advised. However, they can be useful to confirm complete pathological response when biopsies are obtained or to determine relapse in studies where recurrence following complete response or surgical resection is an endpoint.

Tumor markers alone cannot be used to assess objective tumor response. If markers are initially above the upper normal limit, however, they must normalize for a patient to be considered in complete response.

Cytology, Histology: These techniques can be used to differentiate between PR and CR in rare cases if required by protocol (for example, residual lesions in tumor types such as germ cell tumors, where known residual benign tumors can remain). When effusions are known to be a potential adverse effect of treatment (e.g. with certain taxane compounds or angiogenesis inhibitors), the cytological confirmation of the neoplastic origin of any effusion that appears or worsens during treatment when the measurable tumor has met criteria for response or stable disease is advised to differentiate between response or stable disease and progressive disease

Table 14. Assigning Time Point Response for iRECIST

Target Lesions*	Non-Target Lesions*	New Lesions *	Time Point Response	
			No prior iUPD**	Prior iUPD**; ***
iCR	iCR	No	iCR	iCR
iCR	Non-iCR/Non-iUPD	No	iPR	iPR
iPR	Non-iCR/Non-iUPD	No	iPR	iPR
iSD	Non-iCR/Non-iUPD	No	iSD	iSD
iUPD with no change OR decrease from last TP	iUPD with no change OR decrease from last TP	Yes	NA	NLs confirms iCPD if NLs were previously identified and increase in size (≥5 mm in SOM for NLT or any increase for NLNT) or number. If no change in NLs (size or number) from last TP, remains iUPD
iSD	iUPD	No	iUPD	Remains iUPD unless iCPD confirmed based in further increase in size of NT disease (need not meet RECIST 1.1 criteria for unequivocal PD)
iUPD	Non-iCR/Non-iUPD	No	iUPD	Remains iUPD unless iCPD confirmed based on:<br>• further increase in SOM of at least 5 mm, otherwise remains iUPD

Target Lesions*	Non-Target Lesions*	New Lesions *	Time Point Response	
			No prior iUPD**	Prior iUPD**; ***
iUPD	iUPD	No	iUPD	Remains iUPD unless iCPD confirmed based on further increase in:<br>• previously identified T lesion iUPD SOM ≥5 mm and/or<br>• NT lesion iUPD (prior assessment - need not be unequivocal PD)
iUPD	iUPD	Yes	iUPD	Remains iUPD unless iCPD confirmed based on further increase in:<br>• previously identified T lesion iUPD ≥5 mm and/or<br>• previously identified NT lesion iUPD (need not be unequivocal) and /or<br>• size or number of new lesions previously identified
Non-iUPD/PD	Non-iUPD/PD	Yes	iUPD	Remains iUPD unless iCPD confirmed based on<br>• increase in size or number of new lesions previously identified

iCR = immune complete response; iPR = immune partial response; iSD = immune stable disease; iUPD = immune unconfirmed progression; iCPD = immune confirmed progression; NL = new lesions; NLNT = new lesion non target; T = target; TP = time point; NA = not applicable; NE = not evaluable/evaluated

* Using RECIST 1.1 principles. If no PSPD occurs, RECIST 1.1 and iRECIST categories for CR, PR and SD would be the same. ** in any lesion category. *** previously identified in assessment immediately prior to this TP.

All patients will have their Best Overall Response by iRECIST (iBOR) from the start of study treatment until the end of treatment classified as outlined below.

Table 15. Assigning Best Overall Response for iRECIST

TPR1	TPR2	TPR3	TPR4	TPR5	iBOR
iCR	iCR, iPR, iUPD, NE	iCR, iPR, iUPD, NE	iUPD	iCPD	iCR
iUPD	iPR, iSD, NE	iCR	iCR, iPR, iSD, iUPD, NE	iCR, iPR, iSD, iUPD, iCPD, NE	iCR
iUPD	iPR	iPR, iSD, iUPD, NE	iPR, iSD, iUPD, NE, iCPD	iPR, iSD, iUPD, NE, iCPD	iPR
iUPD	iSD, NE	PR	iPR, iSD, iUPD, NE	iPR, iSD, iUPD, iCPD, NE	iPR

TPR1	TPR2	TPR3	TPR4	TPR5	iBOR
iUPD	iSD	iSD, iUPD, NE	iSD, iUPD, iCPD, NE	iSD, iUPD, ICPD, NE	iSD
iUPD	iCPD	Anything	Anything	Anything	iCPD
iUPD	iUPD	iCPD	Anything	Anything	iCPD
iUPD	NE	NE	NE	NE	iUPD

iCR = immune complete response; iPR = immune partial response; iSD = immune stable disease; iUPD = immune unconfirmed progression; iCPD = immune confirmed progression; iBOR = Best Overall Response by iRECIST; NE = not evaluable that cycle; TPR = time point response

Table assumes a randomized study where confirmation of CR or PR is not required.

Designation "I" for BOR can be used to indicate prior iUPD to aid in data interpretation.

For patients with non-target disease only at baseline, only CR or non-CR/non-PD can be assigned at each TPR but is not shown in the table for ease of presentation.

APPENDIX C: ECOG PERFORMANCE STATUS

ECOG PERFORMANCE STATUS*	
**GRADE**	**ECOG**
0	Fully active, able to carry on all pre-disease performance without restriction
1	Restricted in physically strenuous activity but ambulatory and able to carry out work of a light or sedentary nature, e.g., light housework, office work
2	Ambulatory and capable of all self-care but unable to carry out any work activities. Up and about more than 50% of waking hours
3	Capable of only limited self-care, confined to bed or chair more than 50% of waking hours
4	Completely disabled. Cannot carry on any self-care. Totally confined to bed or chair
5	Dead

*As published in: Am. J. Clin. Oncol.: Oken, M.M., Creech, R.H., Tormey, D.C., Horton, J., Davis, T.E., McFadden, E.T., Carbone, P.P.: Toxicity and Response Criteria of The Eastern Cooperative Oncology Group. Am J Clin Oncol 5:649-655, 1982.

APPENDIX D: PRINCIPAL INVESTIGATOR SIGNATURE OF AGREEMENT PAGE

INVESTIGATORS AGREEMENT

This document is a confidential communication of Geneos Therapeutics. The recipient agrees that no unpublished information contained herein will be published or disclosed without prior written permission of Geneos Therapeutics. However, this document may be disclosed to appropriate Institutional Review Boards, ethics review committees, or authorized representatives of the Investigator or regulatory agencies under the condition that they are requested to respect the confidentiality of the document.

The signature of the Principal Investigator below constitutes his/her agreement to comply with the contents of this Protocol.

____

Principal Investigator’s Name (Printed)

____

Principal Investigator’s Institution

____

Principal Investigator’s Signature Date
