# Supplementary material for: Personalized neoantigen vaccine and pembrolizumab in advanced hepatocellular carcinoma: a phase 1/2 trial
Source: Nat Med. 2024 Apr 7;30(4):1044–53. doi: 10.1038/s41591-024-02894-y (PMC11031401; doi:10.1038/s41591-024-02894-y)
Supplement: Supplementary file 2 — Reporting Summary [file 41591_2024_2894_MOESM2_ESM.pdf]

nature portfolio

Corresponding author(s): NMED-A123912D-Z

Last updated by author(s): Feb 16, 2024

Reporting Summary

Nature Portfolio wishes to improve the reproducibility of the work that we publish. This form provides structure for consistency and transparency in reporting. For further information on Nature Portfolio policies, see our Editorial Policies and the Editorial Policy Checklist.

Statistics

For all statistical analyses, confirm that the following items are present in the figure legend, table legend, main text, or Methods section.

n/a	Confirmed	
☐	☒	The exact sample size (*n*) for each experimental group/condition, given as a discrete number and unit of measurement
☐	☒	A statement on whether measurements were taken from distinct samples or whether the same sample was measured repeatedly
☐	☒	The statistical test(s) used AND whether they are one- or two-sided<br>*Only common tests should be described solely by name; describe more complex techniques in the Methods section.*
☒	☐	A description of all covariates tested
☒	☐	A description of any assumptions or corrections, such as tests of normality and adjustment for multiple comparisons
☐	☒	A full description of the statistical parameters including central tendency (e.g. means) or other basic estimates (e.g. regression coefficient) AND variation (e.g. standard deviation) or associated estimates of uncertainty (e.g. confidence intervals)
☐	☒	For null hypothesis testing, the test statistic (e.g. *F*, *t*, *r*) with confidence intervals, effect sizes, degrees of freedom and *P* value noted<br>*Give P values as exact values whenever suitable.*
☒	☐	For Bayesian analysis, information on the choice of priors and Markov chain Monte Carlo settings
☒	☐	For hierarchical and complex designs, identification of the appropriate level for tests and full reporting of outcomes
☒	☐	Estimates of effect sizes (e.g. Cohen's *d*, Pearson's *r*), indicating how they were calculated

Our web collection on statistics for biologists contains articles on many of the points above.

Software and code

Policy information about availability of computer code

Data collection	CTL ImmunoSpot S6 Ultimate V analyzer<br>LSRFortessa (BD Biosciences)<br>FACSDiva software (version 8.0.1)<br>NovaSeq 6000 sequencer (Illumina)<br>HiSeq Software Suite (Illumina; version 2.2.68)
Data analysis	GraphPad Prism (GraphPad Software LLC, version 9.0.1)<br>ImmunoSpot software (version 5.1)<br>FlowJo (BD, version 10.4)<br>ImmunoSEQ Analyzer toolset (Adaptive, version 3.0)<br>Cellranger pipeline (10X Genomics, version 7.1)<br>R (version 4.2.2)<br>Seurat package (version 4.1.1)<br>scRepertoire package (version 1.8.0)<br>GOAL version 7.0 (gene optimization algorithm licensed from Inovio Pharmaceuticals)

For manuscripts utilizing custom algorithms or software that are central to the research but not yet described in published literature, software must be made available to editors and reviewers. We strongly encourage code deposition in a community repository (e.g. GitHub). See the Nature Portfolio guidelines for submitting code & software for further information.

Data

Policy information about availability of data

All manuscripts must include a data availability statement. This statement should provide the following information, where applicable:

- Accession codes, unique identifiers, or web links for publicly available datasets
- A description of any restrictions on data availability
- For clinical datasets or third party data, please ensure that the statement adheres to our policy

Single-cell RNA sequencing data are deposited in the Gene Expression Omnibus (GEO) under accession number GSE GSE255830. TCRβ sequencing data can be found at the open access immuneACCESS® database under the DOI 10.21417/RP2024NM. The TCR constructs used to evaluate T cell vaccine specificity are deposited in GenBank under accession numbers PP316119 (Pt#8_TCR1), PP316120 (Pt#8_TCR2), PP316121 (Pt#8_TCR3), PP316116 (Pt#5_c3-1), PP316117 (Pt#5_c3-2), and PP316118 (Pt#5_c6). Deidentified individual participant clinical data that underlie the results reported in this article are available for transfer. Interested investigators can obtain and certify the data transfer agreement and submit requests to the corresponding author (NYS). Investigators and institutions who consent to the terms of the data transfer agreement form, including, but not limited to, the use of these data for the purpose of a specific project and only for research purposes, and to protect the confidentiality of the data and limit the possibility of identification of participants in any way whatsoever for the duration of the agreement, will be granted access. Geneos will then facilitate the transfer of the requested deidentified data. This mechanism is expected to be via a Geneos Secure File Transfer Service, but Geneos reserves the right to change the specific transfer method at any time, provided appropriate levels of access authorization and control can be maintained. Source data are provided with this paper.

Human research participants

Policy information about studies involving human research participants and Sex and Gender in Research.

Reporting on sex and gender	Patient's sex was captured in the clinical database for all 36 patients. Data findings apply to a single sex, male or female. Patient's sex was considered in the study design, however patients were not stratified based on sex or gender, nor included or excluded from the study based on sex and gender. Gender was not collected in the clinical database.<br>Consent has been provided for all patients to share data regarding sex. The 36 patients enrolled included 11 females and 25 males.
Population characteristics	Eligible patients were ≥ 18 with advanced HCC who progressed during or were intolerant to 1st line TKI (sorafenib or lenvatinib) treatment. Eligibility criteria included age of ≥18 years; a confirmed diagnosis of HCC; BCLC Stage C disease or BCLC Stage B disease; Child-Pugh Class A; a predicted life expectancy of greater than 6 months; a performance status of 0 or 1 using the ECOG Performance Scale; and measurable disease based on RECIST 1.1. Key exclusion criteria were the use of prior systemic therapy for HCC other than sorafenib or lenvatinib, or active autoimmune disease.<br><br>Patients enrolled: 36<br>Age: mean 66.5 years, range 40-83<br>Female: 30.6%<br>Male: 69.4%<br>ECOG - Performance status 0: 69.4%<br>Previous 1L TKI treatment: Lenvatinib (91.6%), Sorafenib (5.6%), both Lenvatinib and Sorafenib (2.8%) as 1L.<br>PVI: 19.4%
Recruitment	Patients were recruited from Johns Hopkins Hospital, Icahn School of Medicine at Mount Sinai and New Zealand Clinical Research by our clinical study investigators who treat patients with advanced HCC. Patients were identified by the investigators by reviewing the site's patient database, discussions at tumor board and ensuring that patients met inclusion/exclusion criteria as stated in the protocol. No biases identified or impact to results.
Ethics oversight	The protocol of GT-30 clinical study was approved by the appropriate institutional review board (IRB) or ethics committees at each participating including Johns Hopkins Medicine Review Boards (CR00039002 / IRB00227771), Icahn School of Medicine-Program for the Protection of Human Subjects (20-00076 GCO#1), and Northern A Health and Disability Ethics committee (Ethics ref: 20/NTA). Written informed consent was obtained from each patient prior to the patient participating in the trial. The study was registered at https://clinicaltrials.gov/ under the identifier NCT04251117.

Note that full information on the approval of the study protocol must also be provided in the manuscript.

Field-specific reporting

Please select the one below that is the best fit for your research. If you are not sure, read the appropriate sections before making your selection.

☒ Life sciences ☐ Behavioural & social sciences ☐ Ecological, evolutionary & environmental sciences

For a reference copy of the document with all sections, see nature.com/documents/nr-reporting-summary-flat.pdf

Life sciences study design

All studies must disclose on these points even when the disclosure is negative.

Sample size	36 patients. To evaluate the secondary endpoint of objective response rate (ORR) per response evaluation criteria in solid tumors version 1.1 (RECIST v1.1) by investigator review with a null hypothesis of an ORR of 16.9% and an alternative hypothesis of an ORR of 33.1%, 36 subjects are enrolled using an exact 1-sided binomial test of a single proportion. The overall power for this test with approximately 36 subjects is 80% assuming an one-sided alpha (α)=0.10. A statistically significant difference at the one-sided 5% level will be declared for an observed ORR of at least 30.5% for 36 subjects (11/36). The operating characteristics of this design are calculated using the exact binomial distribution.
Data exclusions	At the time of data cutoff, two patients discontinued therapy due to unrelated SAEs after the first dose and third dose of therapy, respectively, and were deemed unevaluable but both patients were included in the mITT analysis as non-responders. The vaccine-induced immune response was evaluated by IFNg ELISpot as planned in 22 of the first 24 enrolled patients. Two patients were not evaluated in this analysis due to PBMC sample availability.
Replication	All the data included in the manuscript are replicable. ELISpot and flow cytometry assays performed at FlowMetric are described in detail in Methods section with references and we have documented the source material used. All ELISpot and flow cytometry assays were performed with 3 technical replicates. Proper controls were used to ensure clear interpretation of the experimental results. TCR sequencing was performed by Adaptive biotechnologies once due to clinical sample availability. Single cell RNA/TCR sequencing was performed once due to clinical sample availability at the Center for Applied Genomics, Children's Hospital of Philadelpha. ctDNA analysis was performed by Personalis once due to clinical sample availability.
Randomization	No randomization was performed for this single-arm Phase 1/2 clinical study.
Blinding	All patients received the same treatment (GNOS-PV02+pIL12+Pembrolizumab), therefore investigators were not blinded to study treatment.

Reporting for specific materials, systems and methods

We require information from authors about some types of materials, experimental systems and methods used in many studies. Here, indicate whether each material, system or method listed is relevant to your study. If you are not sure if a list item applies to your research, read the appropriate section before selecting a response.

Materials & experimental systems

n/a	Involved in the study	
☐	☒	Antibodies
☐	☒	Eukaryotic cell lines
☒	☐	Palaeontology and archaeology
☒	☐	Animals and other organisms
☐	☒	Clinical data
☒	☐	Dual use research of concern

Methods

n/a	Involved in the study	
☒	☐	ChIP-seq
☐	☒	Flow cytometry
☒	☐	MRI-based neuroimaging

Antibodies

Antibodies used	Anti-CD107a–APC (clone H4A3, BioLegend #328619)<br>Anti-CD3-BV711 (clone UCHT1, BD Biosciences #563725)<br>Anti-CD4-BUV395 (clone RPA-T4, BD Biosciences #563550)<br>Anti-CD8-BUV805 (clone RPA-T8, BD Biosciences #612889)<br>Anti-CD69-BV421 (clone FN50, BD Biosciences #562884)<br>Dump markers including anti-CD14/-CD16/-CD19-APC-H7 (clone MΦP9 cat.#560180, 3G8 cat.#560715, SJ25CI cat.#560177, respectively) (all from BD Biosciences)<br>Anti-IFN-γ-BV786 (clone 4S.B3, BD Biosciences #563731)<br>Anti-Ki67-AF700 (clone B56, BD Biosciences #561277)<br>Anti-CD137-BV605 (clone 4B4-1, BioLegend #309822)<br>Anti-IL-2-FITC (clone MQ1-17H12, BD Biosciences #554565)<br>Anti-GranzymeA-PerCP/Cy5.5 (clone CB9, BioLegend #507216)<br>Anti-Perforin-PE/Dazzle594 (clone dG9, BioLegend #308132)<br>Anti-TNF-α-PE/Cy7 (clone MAb11, BD Biosciences #557647)
Validation	All antibodies used in the flow cytometry experiments reported in the manuscript were purchased from commercial vendors and were validated by them. We did not perform independent validation of these antibodies.<br>Manufacturer's validation statements:<br>BD antibodies: Our manufacturing process adheres to standard operating procedures (SOPs) and guidelines, which are based on ISO requirements and are strictly followed, helping ensure that reagents provide consistent results to help give you assurance of experimental success and confidence in your research. Quality control testing of newly manufactured lots is performed side-by-side

with a previously accepted lot as a control, helping to assure that performance of the new lot is both reliable and consistent. The specificity is confirmed using multiple methodologies that may include a combination of flow cytometry, immunofluorescence, immunohistochemistry or western blot to test staining on a combination of primary cells, cell lines or transfectant models. All flow cytometry reagents are titrated on the relevant positive or negative cells. To save time and cell samples for researchers, test size reagents are bottled at an optimal concentration with the best signal-to-noise ratio on relevant models during the product development. To ensure consistent performance lot-to-lot, each reagent is bottled to match the previous lot MFI.
BioLegend antibodies: All products sold by BioLegend Inc. comply with the requirements of ISO 13485:2016. This includes products labeled as Research Use Only (RUO) or GMP Research Use Only (GMP RUO), Analyte Specific Reagents (ASRs), and In Vitro Diagnostics (IVDs) Including CE-Marked and registered products in selected countries.

Eukaryotic cell lines

Policy information about cell lines and Sex and Gender in Research

Cell line source(s)	Phoenix-AMPHO cells (ATCC, VA)
Authentication	No authentication was performed
Mycoplasma contamination	Cells were confirmed negative for mycoplasma
Commonly misidentified lines (See ICLAC register)	No commonly misidentified lines were used in the study

Clinical data

Policy information about clinical studies

All manuscripts should comply with the ICMJE guidelines for publication of clinical research and a completed CONSORT checklist must be included with all submissions.

Clinical trial registration	NCT04251117
Study protocol	Study protocol is available as part of the supplemental information submitted together with the manuscript.
Data collection	Clinical data were collected in an electronic data capture (EDC) system database, Prism. The EDC is managed by ICON (formally PRA) CRO. Data reported in the EDC mirrors source data collected at the investigative sites located in the USA and New Zealand. Blood and tumor samples were collected during the scheduled clinical visits. The ELISPOT, flow cytometry, TCR and single cell sequencing data were generated and analyzed by FlowMetric, Adaptive Biotechnologies and CHOP, respectively, using the patient's PMBCs. ctDNA data were generated by Personalis using blood and tumor samples. The subject recruitment period was 18Feb2020-14Jun2024. Data collection is still ongoing and survival follow-up information will be collected via telephone calls, subject medical records, and/or clinical visits approximately Q12W for up to 3 years, until death, lost to follow-up, withdrawal of consent, or study termination by Sponsor.
Outcomes	The primary endpoints were safety/tolerability and immunogenicity. Safety and tolerability were monitored closely with changes in physical examination and clinical laboratory analyses. Adverse events were assessed according to CTCAE v5.0. Immunologic analyses of vaccine-specific cellular responses and T cell infiltration into tumors were detected by ELISpot, intracellular cytokine staining and TCR sequencing.<br>Secondary outcomes included efficacy evaluated by RECIST1.1.

Flow Cytometry

Plots

Confirm that:

☒ The axis labels state the marker and fluorochrome used (e.g. CD4-FITC).

☒ The axis scales are clearly visible. Include numbers along axes only for bottom left plot of group (a 'group' is an analysis of identical markers).

☒ All plots are contour plots with outliers or pseudocolor plots.

☒ A numerical value for number of cells or percentage (with statistics) is provided.

Methodology

Sample preparation	In Vitro Stimulation (IVS) and Intracellular Cell Staining. The patient's PBMCs (2.5x10^5 cells) were cultured in growth media (RPMI 10%-FBS), supplemented with a cocktail of IL-2 (20 IU/mL), IL-4 (10 ng/mL), and IL-7 (10 ng/mL) cytokines, and enriched for neoantigen-specific T cells using 10 µg/mL of epitope stimuli. Three days later, cells were washed, and supplemented growth media was replaced. On day 4, epitope stimuli (10 µg/mL) or controls were added and incubated for 1 hour. Then, anti-CD107a–APC (clone H4A3, Biolegend) antibody and Protein Transport Inhibitor Cocktail (1:500 dilution, Invitrogen) were added. After a 5h incubation, cells were stained using fluorescently labeled surface marker antibodies: anti-CD3-BV711 (clone UCHT1, BD Biosciences), anti-CD4-BUV395 (clone RPA-T4, BD Biosciences), anti-CD8-BUV805 (clone RPA-T8, BD Biosciences), anti-CD69-BV421 (clone FN50, BD Biosciences), anti-CD137-BV605 (clone 4B4-1, Biolegend), and dump markers including anti-CD14/-CD16/-CD19-APC-H7 (clone MΦP9, 3G8, SJ25CI, respectively) (all from BD Biosciences). Dead cells were stained using Live Dead Blue solution (1:1000 dilution, Thermo Fisher), followed by overnight fixation and

permeabilization using Fixation/Permeabilization buffers (00-5123-43, 00-5223-56, eBioscience) according to the manufacturer's instructions. Cells were then stained intracellularly in eBioscience permeabilization buffer (00-8333-56) with anti-IFN-γ-BV786 (clone 4S.B3, BD Biosciences), anti-IL-2-FITC (clone MQ1-17H12, BD Biosciences), anti-Ki67-AF700 (clone B56, BD Biosciences), anti-GranzymeA-PerCP/Cy5.5 (clone CB9, Biolegend), anti-Perforin-PE/Dazzle594 (clone dG9, Biolegend), anti-TNF-α-PE/Cy7 (clone MAb11, BD Biosciences) antibodies. Samples were acquired on the LSRFortessa (BD Biosciences) using FACSDiva software version 8.0.1, and FlowJo v.10.4 or later for data analysis.

TCR-engineered constructs: High-frequency T cell clones were identified in patient-derived PBMC post-vaccination by TCR sequencing and single-cell RNA sequencing of analyses. Patient-specific clonal TCR sequences were gene-optimized and inserted into the pMXs-IRES-GFP retroviral plasmid vector containing viral packaging signal, transcriptional and processing elements, and the GFP reporter gene (Genscript, NJ). Both, TRB and TRA were positioned in sequence separated by a P2A (2A peptide derived from the porcine teschovirus-1) cleavage site (TRB-P2A-TRA). Retroviral particles encoding TCR constructs were generated by transfecting Phoenix-AMPHO cells (ATCC, VA) using Lipofectamine 3000 (Thermo Fisher Scientific, MA) and following the manufacturer's instructions. Unvaccinated (pre-treatment) patient-derived PBMC (1x10^6 cells) were retrovirally transduced to express the selected TCRs as previously described47. Cells were cultured in RPMI media supplemented with 10%-FBS, 50 U/mL IL-2, and 1 ng/ml of IL-7 (Peprotech, NJ) in a 5% CO2 humidified incubator for 10 days. Cell culture media was refreshed every 2-3 days. TCR-engineered T cells (GFP positive) from unvaccinated PBMC were stimulated for 6 hours with indicated concentrations of epitope pools, and the expression of CD69 was evaluated by flow cytometry.

Instrument

Samples were acquired on the LSRFortessa (BD Biosciences) using FACSDiva software version 8.0.1

Software

FlowJo (BD, version 10.4)

Cell population abundance

IVS and ICS: cell input, 2.5x10^5 PBMC cells. On the evaluation day, dead cells were stained using Live Dead Blue solution (1:1000 dilution, Thermo Fisher), and excluded at the beginning of the gating process. The average of live cells across samples was 86.5%. Samples with less than 30.0% viability were excluded from the analysis.

TCR-engineered constructs: 0.3x10^6 cells were plated in a 96-well plate at the beginning of the assay. Six hours post-stimulation, dead cells were stained using Live Dead Blue solution (1:1000 dilution, Thermo Fisher), and excluded at the beginning of the gating process. Only viable and GFP-positive (~30.0% of parent) T cells were included in the analysis.

Gating strategy

IVS and ICS: Cells were gated on PBMCs (forward scatter (FSC)-A versus side scatter (SSC)-A), singlets (FSC-A versus FSC-H), live cells (FSC-A versus Live/Dead), lymphocytes (CD3+ versus dump markers), CD8 or CD4 T cells (CD8 versus CD4) and cytokine+ cells. Representative density plots (patient 22) of individual T cell activity markers CD69+, Ki67+, CD107a+, IFNγ+, and TNFα+ upon stimulation with patient-specific PTCV epitope pools.
Polyfunctionality was assessed via Boolean gating of CD4+ or CD8+cytokine+ populations. T cell activation (CD69 and CD107a) and proliferation (Ki67) were assessed together with the double positive expression of granzyme A (GrzA) and perforin (Prf) to evaluate the cytolytic potential of neoantigen-reactive T cells. Results are represented as % positive cell populations (frequency of parent).

TCR-engineered constructs: Cells were gated on PBMCs (forward scatter (FSC)-A versus side scatter (SSC)-A), singlets (FSC-A versus FSC-H), live cells (FSC-A versus Live/Dead), lymphocytes (CD3), CD8 or CD4 T cells (CD8 versus CD4), GFP positive (CD8 or CD4 versus GFP) and CD69+ cells.

☒ Tick this box to confirm that a figure exemplifying the gating strategy is provided in the Supplementary Information.
